# Paddlewheel Dicobalt Complexes with Metal Centers Having Various Oxidation States

**DOI:** 10.1021/acs.inorgchem.5c02468

**Published:** 2025-09-24

**Authors:** Bo-An Liao, Cian-Wei Yang, Anokh K. Nair, Yi-Chou Tsai

**Affiliations:** † Department of Chemistry, 34881 National Tsing Hua University, Hsinchu 30013, Taiwan; ‡ Department of Chemistry and Frontier Research Center on Fundamental and Applied Sciences of Matters, National Tsing Hua University, Hsinchu 300313, Taiwan

## Abstract

Herein, we report the characterization of a family of two bidentate silyldiamide-stabilized digonal lantern dinuclear cobalt complexes [Co_2_{μ-κ^2^-Ph_2_Si­(N-2,6-^*i*^Pr_2_C_6_H_3_)_2_}]^*x*−^ (*x* = 0 (**1**), 1 (**2**), and 2 (**3**)) in order to study the interaction between two Co atoms. Complexes **1**–**3** can be interconverted by redox reactions. The Co_2_-containing fragments in **1**–**3** are almost isostructural due to the steric congestion of the supporting ligands, leading to their high rigidity. In contrast to the reported shortest Co^I^–Co^I^ bonds in similar digonal dicobalt complexes, the homounivalent dicobalt compound **3** instead has the longest Co–Co bond length among **1**–**3**. The calculated Co–Co bond orders are in agreement with the experimental data, with the order decreasing from 2 (**1**) to 1 (**3**) as the valence of the central Co_2_ unit decreases.

## Graphical abstract

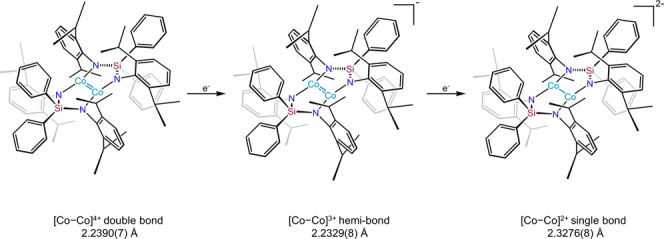

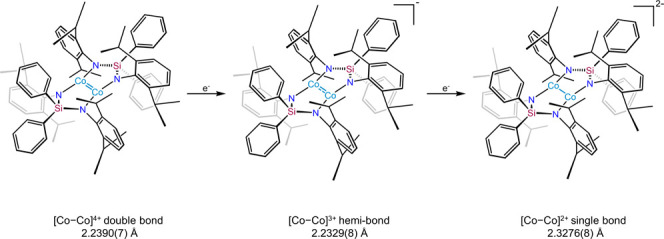

## Introduction

Dinuclear paddlewheel (or lantern) complexes of the general formula M_2_(μ-L)_m_ (M = transition metal; L = bidentate ligand; *m* = 2–4) are found throughout the transition series and display M–M interactions with formal bond orders (fBOs) spanning from 5 down to 0.^1^ The quintuply bonded dichromium and dimolybdenum derivatives adopt digonal (*m* = 2) and trigonal (*m* = 3) lantern geometries,^2^ whereas the isostructural tetragonal (*m* = 4) paddlewheel motif is exemplified by dinickel complexes featuring an fBO of zero.^3^ Although most late-transition metals are confined to the ubiquitous M_2_(μ-L)_4_ paddlewheel architecture, early group 6 metals are unique in giving stable *m* = 2–4 species with M–M bond orders between 3 and 5.^4,5^ Remarkably, cobalt is one of the few late metals that likewise forms all three nuclearities.^6,7^ In dicobalt lantern complexes, the shortest Co^I^–Co^I^ separations coincide with the digonal (*m* = 2) structure, which also exhibits the highest Co–Co fBO of 2; the tetragonal and trigonal cobalt species display progressively lower fBOs of 1 and 0.5, respectively (Figure 1a).^7^

**Figure 1:**
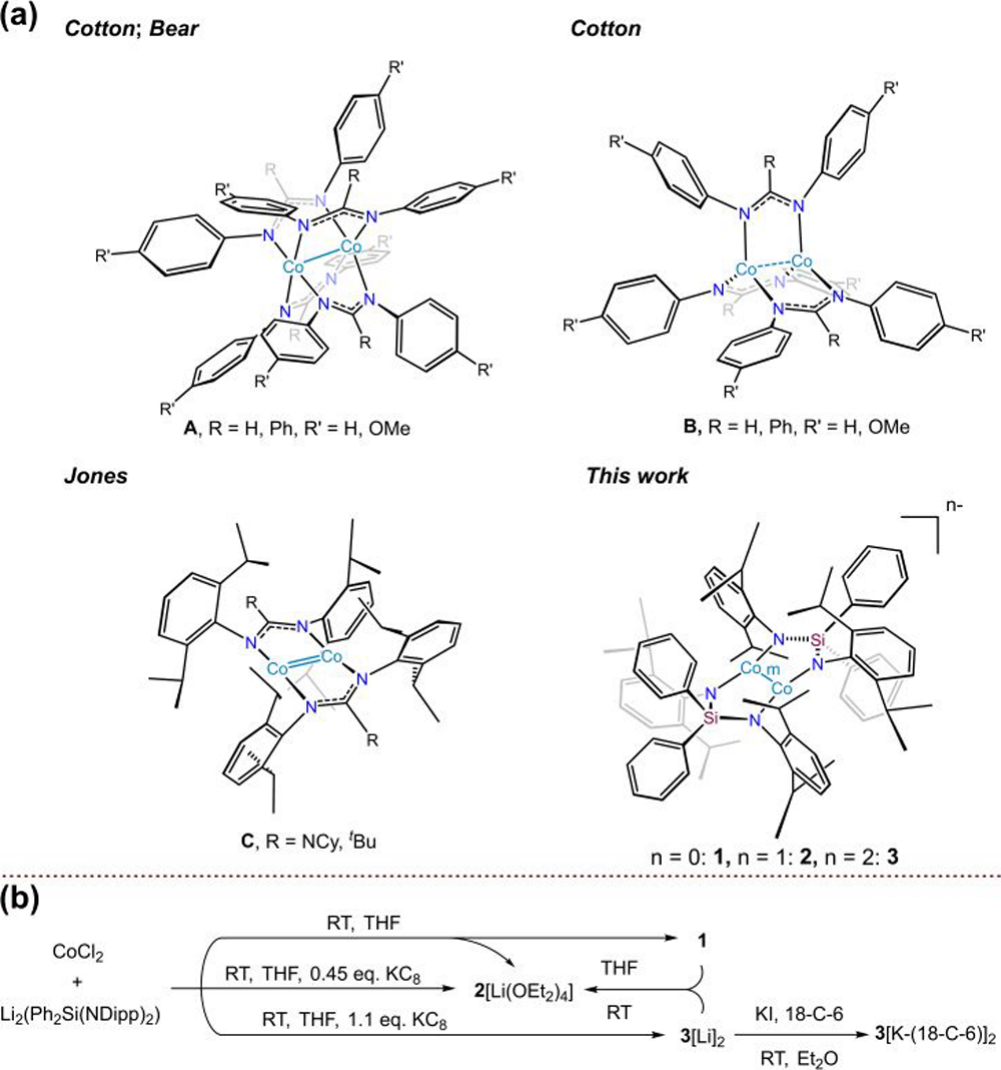
(a) Selected structures of (**A**) tetragonal, (**B**) trigonal, and (**C**) digonal paddlewheel-type dicobalt complexes. (b) Synthesis of complexes **1**–**3** and **3′**.

Dinuclear lantern complexes M_2_(μ-L)_*m*_ adopt geometries that correlate closely with the metals’ formal oxidation state.^1,6^ Stabilized by four monoanionic bidentate bridging ligands, the tetragonal (*m* = 4) motif typically harbors two M^2+^ centers; the digonal (*m* = 2) lantern encloses two M^+^ atoms; and the trigonal (*m* = 3) species most often feature mixed-valence (II/I) bimetallic units. Using the bulky dianionic boraamidinate ligand [PhB­(N-2,6-^*i*^Pr_2_C_6_H_3_)_2_]^2–^, we have characterized a series of digonal lantern complexes [Mo_2_{μ-κ^2^-PhB­(N-2,6-^*i*^Pr_2_C_6_H_3_)_2_}_2_]^*x*−^ (*x* = 0–2), which correspond to Mo_2_^4+^, Mo_2_^3+^, and Mo_2_^2+^ cores.^5d^ As the overall charge increases (or equivalently, as each Mo_2_ unit is reduced by one electron), the Mo–Mo bond length shortens markedly, and the formal bond order rises from 4 to 5; concurrently, the dihedral angle between the two Mo_2_N_2_B five-member rings widens from 139° to 180°.

Two-coordinate mononuclear first-row transition metal-amido complexes in the +2 and +1 oxidation states have been reported,^8^ but their reliance on monodentate bulky ligands precludes any direct M–M interaction. By contrast, a sterically demanding bidentate diamido bridge can enforce the proximity of two metal centers. In this context—and inspired by the rich chemistry of Co–Co bonds^6,7^ —we wish to disclose a series of digonal paddlewheel-type dicobalt complexes, [Co_2_{μ-κ^2^-Ph_2_Si­(N-2,6-^*i*^Pr_2_C_6_H_3_)_2_}_2_]^*x*−^ (*x* = 0–2), supported by the dianionic bidentate amido ligand [Ph_2_Si­(N-2,6-^*i*^Pr_2_C_6_H_3_)_2_]^2–^. This platform allows for systematic interrogation of Co–Co bonding in both homovalent and mixed-valence settings.

## Result and Discussion

Building on our characterization of the first digonal paddlewheel-type quadruply bonded dimolybdenum complex [Mo_2_(μ-κ^2^-Me_2_Si­(N-2,6-^*i*^Pr_2_C_6_H_3_)_2_)_2_],^5a^ we investigated the reaction of CoCl_2_ with Li_2_[Ph_2_Si­(N-2,6-^*i*^Pr_2_C_6_H_3_)_2_] in Et_2_O at room temperature. Although the sterically demanding diamide was expected to furnish the homovalent dicobalt­(II/II) species Co_2_(μ-κ^2^-Ph_2_Si­(N-2,6-^*i*^Pr_2_C_6_H_3_)_2_)_2_ (**1**) in high yield, the reaction instead produced only 8% of **1** alongside 15% of the purple mixed-valent (II/I) complex [Li­(OEt_2_)_4_]­[Co_2_(μ-κ^2^-Ph_2_Si­(N-2,6-^*i*^Pr_2_C_6_H_3_)_2_)_2_] ([Li­(OEt_2_)_4_]­[**2**]) (Figure 1b). The appearance of protonated diamine and amidoamine byproducts in the NMR spectra implicates the diamido ligand’s reducing character in converting Co­(II) to Co­(I). Prereduction of CoCl_2_ with 0.5 equiv of KC_8_ prior to ligand addition boosts the yield of [Li­(OEt_2_)_4_]­[**2**] to 60%, and quantitative conversion of **1** into [Li­(OEt_2_)_4_]­[**2**] is achieved by KC_8_ reduction, confirming the facile Co­(II)/Co­(I) redox interconversion.

Single-crystal X-ray diffraction (Figures 2a and S2) shows that **1** and the anionic Co_2_-containing fragment **2** are essentially isostructural digonal paddlewhees. In both, two Co centers are bridged by two diamido ligands and lie in a nearly perfect plane defined by four N donors, with N(1)–Co(1)–Co(1′)–N(2) dihedral angles of 0.33(13)° for **1** and 0.25(11)° for **2**. Each Co_2_N_2_Si five-membered ring, however, adopts a puckered conformation, and together they form a chairlike Co_2_N_4_Si_2_ eight-membered core, possessing *C*_2h_ symmetry (Figure 2b). By contrast, the analogous dinuclear zinc and cadmium complexes [M_2_(μ-κ^2^-Me_2_Si­(N-2,6-^*i*^Pr_2_C_6_H_3_)_2_)_2_] (M = Zn, Cd) feature four roughly planar M_2_N_2_Si rings,^9,10^ indicating that the increased steric demand around Si in **1** and **2** enforces puckering.

**Figure 2:**
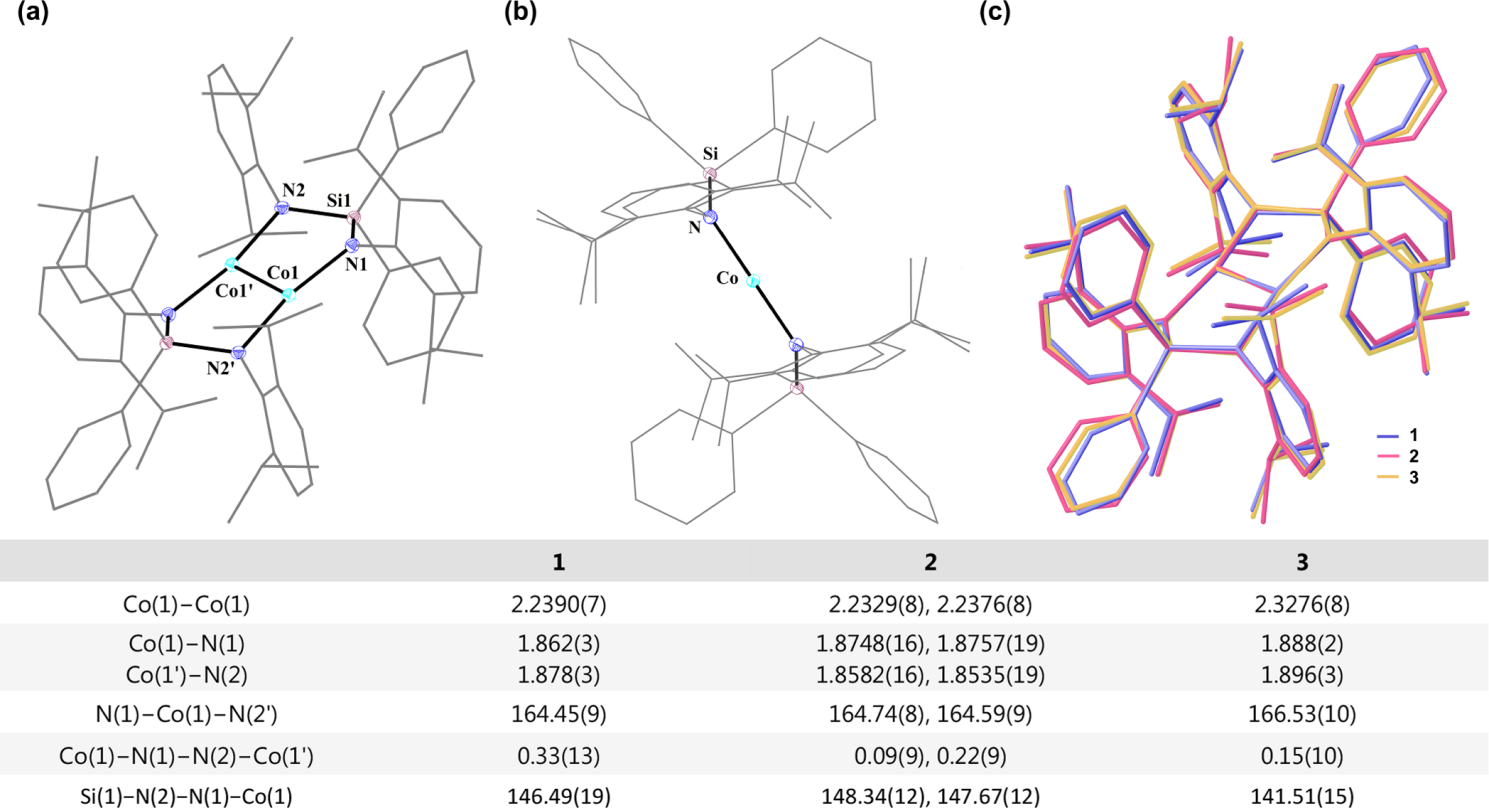
(a) Solid structure of **1**. Symmetry-generated atoms are labeled by the ′ sign.^*a*^ (b) Side view of **1**.^*a*^ (c) Overlay of **1**, **2**, and **3**. Structures are drawn in wireframes for clarity. Selected bond lengths (Å) and bond angles (°) are listed below.

The molecular shapes of **1** and **2** reveal the bonding interactions between the two Co atoms. The two-coordinate Co­(II) and Co­(I) amido complexes Co­[N­(SiR_3_)-2,6-^*i*^Pr_2_C_6_H_3_]_2_ (R = Me, ^*i*^Pr)^8a,8b^ and [Co­{N­(SiMe_3_)-2,6-^*i*^Pr_2_C_6_H_3_}_2_]^−^ ^8c^ show a perfectly linear geometry around the Co centers, but both planar core N_4_Co_2_ units of **1** and **2** are instead concave, as reflected in N–Co–N bond angles of 164.45(9)° and 164.74(8)°, respectively. The Co–Co separation in **1** is 2.2390(7) Å, markedly shorter than the 2.26–2.37 Å range observed in amidinate-stabilized tetragonal Co^II^ paddlewheels (**A**),^7b–7e^ and within the known Co–Co double-bond interval (2.26 ± 0.13 Å).^6^ Although Co–Co double bonds have previously been confined to homovalent Co^I^ dimers,^7a,7i,11^ the mixed-valent **2** exhibits an even shorter Co–Co bond length of 2.2329(7) Å, underscoring the exceptional bond contraction achieved in our system.

Linear mononuclear amides M­[N­(SiMe_3_)­Ar]_2_ (M = Fe, Co, Ni; Ar = 2,6-^*i*^Pr_2_C_6_H_3_) exhibit M–N bond lengths substantially shorter than those in their one-electron-reduced anions [M­{N­(SiMe_3_)-2,6-^*i*^Pr_2_C_6_H_3_}_2_]^−^, a consequence of diminished electrostatic attraction upon reduction.^8a,8c^ In contrast, complexes **1** and **2** display nearly identical Co–N bond lengths, averaging 1.870(3) Å for **1** and 1.8665(16) Å for **2**, which are appreciably shorter than the 1.91–1.99 Å range typical of dicobalt amidinato and guanidinato complexes.^7^ This bond contraction reflects the superior π-donor strength of the dianionic bridge [Ph_2_Si­(N-2,6-^*i*^Pr_2_C_6_H_3_)_2_]^2–^, which enhances Co–N π-bonding relative to amidinate or guanidinate ligands.

The cyclic voltammogram of **1** (0.1 M [^*n*^Bu_4_N]­PF_6_ in THF; Figure 3a) displays two reversible cathodic waves at −0.40 V and – 2.81 V vs Fc+/Fc. Relative to the open-circuit potential (*E*_ocp_ = −0.24 V) (Figure S23), these processes can be assigned to the (II/II) → (II/I) and (II/I) → (I/I) reductions, respectively. The difficulty to reduce the mixed-valent **2** to the homounivalent dicobalt species is caused by the π-donating N donors,^14^ which destabilize the Co–Co π* manifold and thus result in a far more negative potential to reduce **2.**

**Figure 3:**
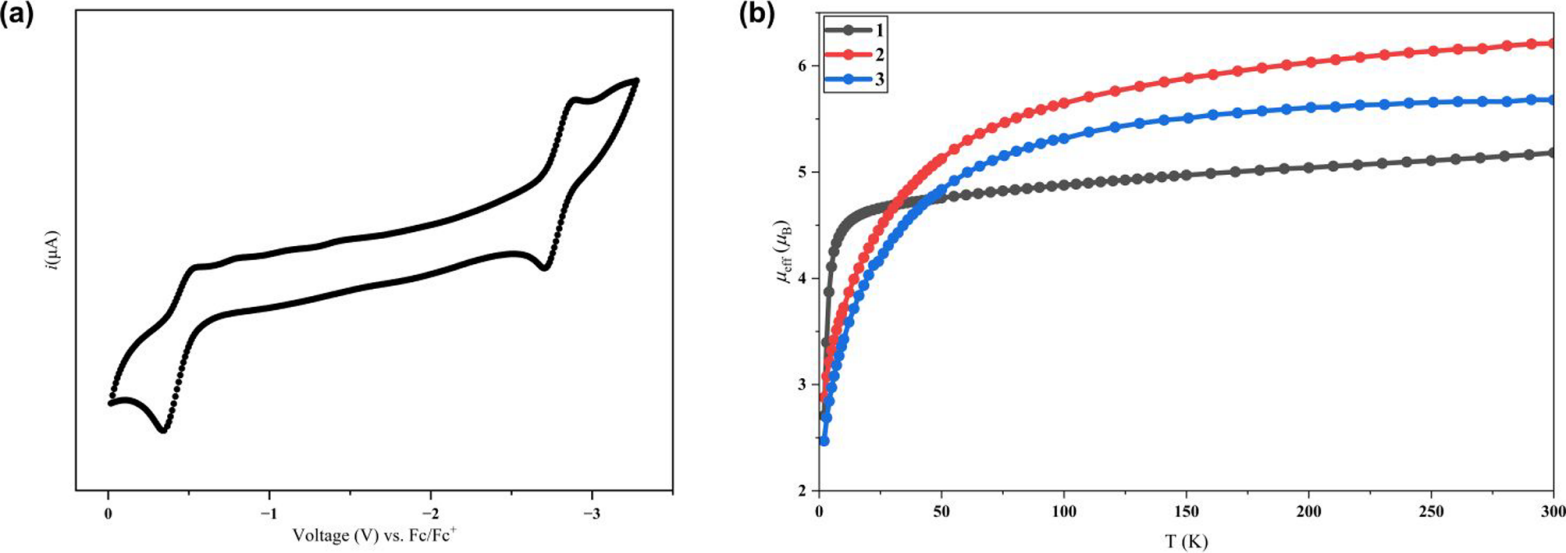
(a) Cyclic voltammogram of **1** (0.1 M [^*n*^Bu_4_N]­PF_6_ in THF). (b) Temperature-dependent effective magnetic moment plot of **1**–**3** from 2 to 300 K.

Guided by these data, we targeted the homounivalent dicobalt species, the one-electron-reduced analogue of **2**. Experimentally, the reaction of CoCl_2_ with one equivalent of Li_2_[Ph_2_Si­(N-2,6-^*i*^Pr_2_C_6_H_3_)_2_] and 1.1 equiv of KC_8_ in THF cleanly delivered Li_2_[Co_2_(μ-κ^2^-Ph_2_Si­(N-2,6-^*i*^Pr_2_C_6_H_3_)_2_)_2_] ([Li]_2_[**3]**) in 93% isolated yield (Figure 1b). Although suitable single crystals of **3** could not be obtained directly, ion-exchange of its potassium salt with KI/18-crown-6 in Et_2_O afforded crystalline [(THF)_2_K⊂(18-crown-6)]_2_[Co_2_(μ-κ^2^-Ph_2_Si­(N-2,6-^*i*^Pr_2_C_6_H_3_)_2_)_2_] ([K-18-C-6]_2_[**3**]), whose solid-state structure was confirmed by X-ray diffraction. The Co_2_-containing dianionic fragment **3** closely parallels the geometries observed in **1** and in the monoreduced form of **2** (Figure 2c), yet the Co–Co separation in **3** is significantly elongated (2.3676(8) Å), consistent with attenuation of metal–metal bonding upon reduction. Likewise, the Co–N distances (1.888(3) and 1.896(2) Å) are modestly longer than those in **1**, reflecting increased electron density at the metal centers and weakened electrostatic interactions with the silyl-bridged diamide donors. Compared to the analogous homounivalent reference complex **C**, where Co–Co bonds measure 2.1345(7) and 2.1404(10) Å and Co–N distances range from 1.9185(14) to 1.9276(14) Å, the elongation of Co–Co and contraction of Co–N bonds in **3** suggest that the Ph_2_Si­(N-2,6-^*i*^Pr_2_C_6_H_3_)_2_ ligand imposes a particularly strong π-donating influence.

Variable-temperature magnetic susceptibility measurements of **1**–**3** were carried out by SQUID in the 2–300 K range. **1** exhibits an effective magnetic moment of μ_eff_ = 5.18 μ_B_ at 300 K (Figures 3b, S16 and S17), consistent with an *S* = 2 quintet state; its room-temperature solution moment by the Evans method^12^ (μ_eff_ = 4.71 μ_B_) agrees well with the solid-state data. Complex **2** shows μ_eff_ = 6.21 μ_B_ as determined by SQUID (Figures 3b, S18 and S19) and 5.84 μ_B_ in solution, consistent with an *S* = 5/2 spin-only system. Lastly, complex **3** behaves as high-spin states (Figures 3b, S20 and S21), with its solid-state (μ_eff_ = 5.68 μ_B_) and solution (μ_eff_ = 4.89 μ_B_) moments corresponding to an *S* = 2 spin state. Although their spin counts differ, these three compounds exhibit similar paramagnetic behavior, mirroring that reported for earlier digonal^7a^ and trigonal^7h^ paddlewheel dicobalt systems. Below about 100 K, the moments drop, a decrease ascribed to zero-field splitting. Fitting the data yields axial (*D*) and rhombic (*E*) parameters of *D* = 6.26 cm^–1^, *E* = 2.08 cm^–1^ for **1**; *D* = 66.26 cm^–1^, *E* = −1.17 cm^–1^ for **2**; and *D* = −77.51 cm^–1^, *E* = −0.12 cm^–1^ for **3**.^13^

DFT calculations at the BP86 level^15^ on simplified [Co_2_{μ-κ^2^-Me_2_Si­(NMe)_2_}_2_]^*x*−^ models reproduce the experimentally observed spin states of *S* = 2, 5/2, and 2 for complexes **1**, **2**, and **3**, respectively (Tables S11–S13). Geometry optimizations of the full cobalt fragments (neutral **1**, monoanion **2**, and dianion **3**; Tables S14–S16) closely match the crystallographic metrics and reveal that the minimal Me_2_Si­(NMe)_2_ models converge to planar Co_2_N_4_Si_2_ cores, whereas the bulky Ph_2_Si­(N-2,6-^*i*^Pr_2_C_6_H_3_)_2_ ligands enforce chairlike Co_2_N_4_Si_2_ frameworks (Figure S24).

Mulliken spin-density analysis (Figure S25) shows nearly equal metal spin populations: 1.745/1.686 in **1**, 2.111/2.117 in **2**, and 1.851/1.857 in **3**, indicating highly covalent Co–Co bonding. The perpendicular spin density on the N donors (average per-*N* values of 0.135, 0.144, and 0.050 for **1**, **2**, and **3**, respectively) reports on Co–N π-interaction: the N/Co spin-population ratios (0.157 for **1** and 0.136 for **2**) are comparable but drop to 0.054 in **3**, demonstrating significantly weaker π-bonding in the dianionic species.

Analysis of the bonding in **1** (Figure 4a) was carried out by inspecting its frontier molecular orbitals (fMOs; Figures S26 and S27) and natural bond orbitals (NBOs; Figures S32–S34). The α-spin HOMO (*d*_*xy*_ – *d*_*xy*_) and HOMO–6 (*d*_x2–y2_ – *d*_x2–y2_) define the two Co–Co δ* antibonding orbitals, while α-HOMO–1 (*d*_*xy*_ + *d*_*xy*_) and HOMO–16 (*d*_x2–y2_ + *d*_x2–y2_) correspond to the two δ bonding components. Co–N π* interactions are also evident in both α-HOMO and α-HOMO–1. Further down in energy, α-HOMO–17 (*d*_*xz*_ – *d*_*xz*_) and HOMO–23 (*d*_*yz*_ – *d*_*yz*_) represent Co–Co π* antibonding orbitals, whereas α-HOMO–25 (*d*_*yz*_ + *d*_*yz*_) and HOMO–28 (*d*_*xz*_ + *d*_*xz*_) are the π bonding counterparts. The σ-bonding combination of the dz^2^ orbitals appears as α-HOMO–39 (*d*_z2_ + *d*_z2_).

**Figure 4:**
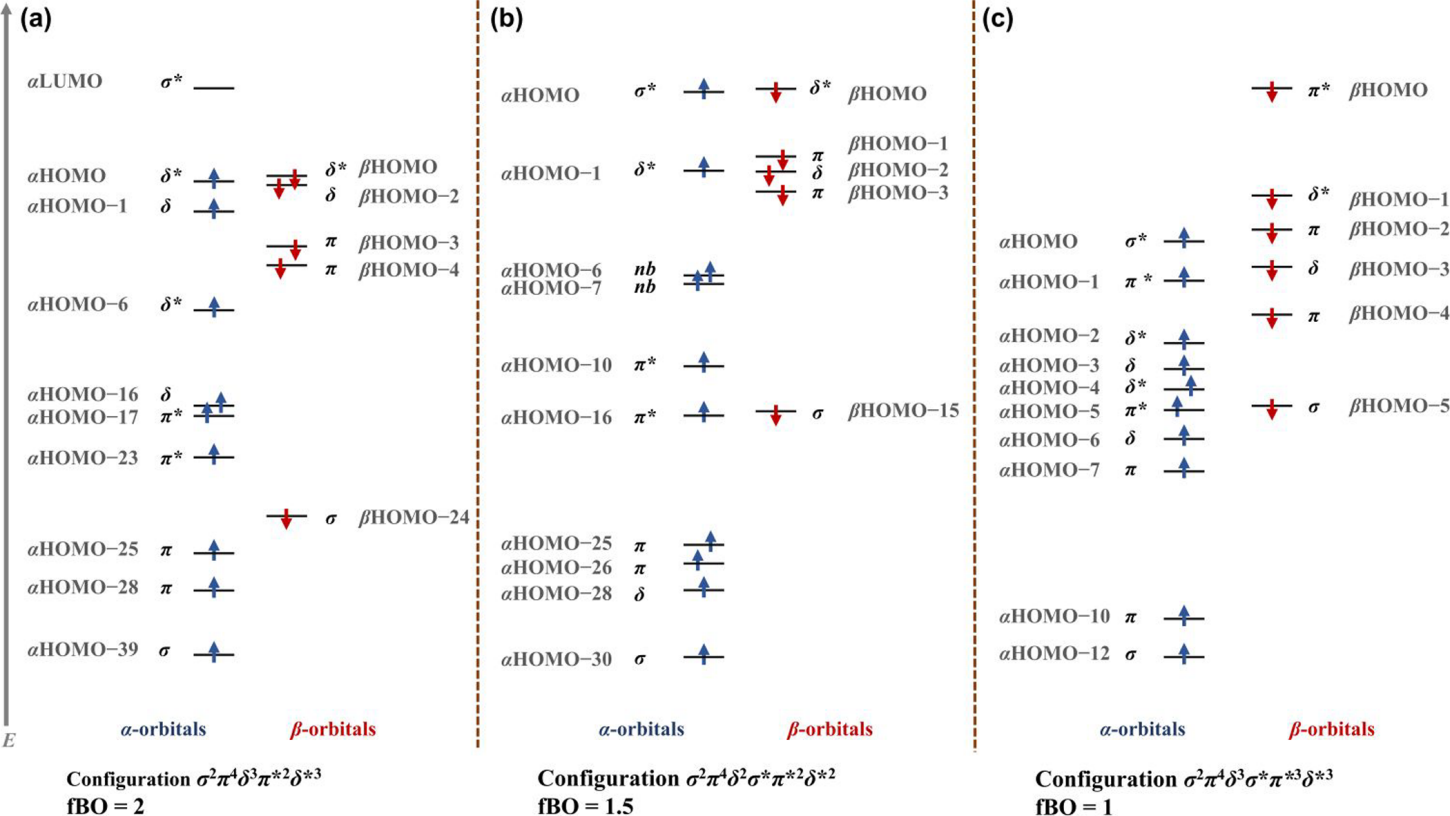
Molecular orbital manifold diagrams of (a) **1**, (b) **2**, and (c) **3**.

In the β manifold, the HOMO (*d*_x2–y2_ – *d*_x2–y2_) is again a δ* orbital; β-HOMO–3 and −4 arise from π interactions between *d*_*yz*_ and *d*_*xz*_ pairs, respectively. The δ bonding combination (*d*_x2–y2_ + *d*_x2–y2_) and σ bonding (*d*_z2_ + *d*_z2_) are found in β-HOMO–2 and β-HOMO–24, respectively. Taken together, the fMO electron configuration of **1** can be described as σ^2^π^4^δ ^3^π*^2^δ*^3^, consistent with a formal Co=Co double bond.

NBO analysis further characterizes the Co–Co σ bond as an *spd*-hybridized interaction: the α orbital contains 0.97 e, shared roughly 52% on Co1 (*sp* ^0.06^ *d* ^9.29^) and 48% on Co2 (*sp* ^0.07^ *d* ^9.08^), while the β orbital (0.97 e) is split 50% on each cobalt (Co1 *sp* ^0.07^ *d* ^7.16^; Co2 *sp* ^0.07^ *d* ^7.59^). The out-of-plane π bond (0.99 e) is formed by *d*_*xz*_ overlap (∼50.6% Co1, 49.4% Co2), whereas the in-plane π component (0.65 e) features enhanced *p* character (*p*/*d* ≈ 1.5–1.6; 46.8% Co1, 53.2% Co2). A Wiberg bond index of 2.14 corroborates the double-bond character of the Co–Co interaction.

Complexes **C** also possess a quintet ground state; however, their four unpaired electrons are distributed across two dominant electronic configurations: σ*π*δ*δ* and σ*π*δδ.^7a^ Interestingly, despite this electronic structure, the Co­(I)–Co­(I) double bonds in **C** (2.1345(7) and 2.1404(10) Å) are significantly shorter than the Co–Co bond lengths observed in **1**–**3**. The longer Co–Co bond lengths in **1**–**3** likely arise from two contributing factors. First, the higher oxidation state of the cobalt centers in **1** (Co­(II)) increases electrostatic repulsion between the Co atoms and consequently elongates the Co–Co bond. Second, the strong π-donating nature of the diamide ligand enhances π-interactions with the Co *d*-orbitals, which in turn diminishes direct orbital overlap between the two cobalt centers. In contrast, the formamidinate ligands in **C** are significantly weaker π-donors, or primarily σ-donating, leaving the Co *d* orbitals more available for effective overlap and thus reinforcing the Co–Co interaction. A comparable ligand effect is also observed in molybdenum systems. The silyldiamide-stabilized quadruply bonded dinuclear complex Mo_2_{μ-Me_2_Si­(N-2,6-^*i*^Pr_2_C_6_H_3_)_2_} exhibits a Mo–Mo bond length of 2.1784(12) Å,^5a^ which is 0.0909 Å longer than that of the amidinate-supported species Mo_2_(μ-Cl)­[Cl_2_Li­(OEt_2_)]­[μ-κ^2^-HC­(N-2,6-^*i*^Pr_2_C_6_H_3_)_2_]_2_ (2.0875(4) Å).^5b^ Thus, in both dinuclear cobalt and molybdenum compounds, replacement of a strong π-donating ligand with a weaker one results in a shortening of the metal–metal bond by approximately 0.1 Å.

Frontier-orbital analysis of **2** (Figures 4b, S28 and S29) shows that the extra α-electron occupies the σ* combination of *d*_*z*_^2^ orbitals (α-HOMO). The α-orbital manifold therefore comprises the bonding σ (α-HOMO–39, *d*_*z*_^2^ + *d*_*z*_^2^), two π bonds (α-HOMO–25, *d*_*xz*_ + *d*_*xz*_; α-HOMO–26, *d*_*yz*_ + *d*_*yz*_), a δ bond (α-HOMO–1, *d*_*xy*_ + *d*_*xy*_), the antibonding σ* (α-HOMO, *d*_z2_–*d*_z2_), two π* orbitals (α-HOMO–10, *d*_*yz*_ – d_*yz*_; α-HOMO–16, *d*_*xz*_ – *d*_*xz*_), and the δ* orbital (α-HOMO–28, *d*_*xy*_ – *d*_*xy*_). Notably, α-HOMO–6 and α-HOMO–7 are nonbonding *d*_x2_-_y2_ orbitals localized on each Co center, signifying diradical character at the mixed-valent Co_2_^3+^ core. The β manifold mirrors five of the key orbitals in **1**, namely, the δ* (β-HOMO, *d*_x2–y2_ – *d*_x2–y2_), a π bond (β-HOMO–1, *d*_*xz*_ + *d*_*xz*_), a δ bond (β-HOMO–2, *d*_x2–y2_ + *d*_x2–y2_), another π bond (β-HOMO–3, *d*_*yz*_ + *d*_*yz*_), and the σ bond (β-HOMO–15, *d*_z2_ + *d*_z2_), in order of descending energy.

NBO analysis of **2** (Figures S35–S37) shows that its Co–Co bonding is concentrated in the β manifold. The Co–Co σ orbital (0.96 e) carries very little s/p character, consisting of 50.25% Co1 (*sp* ^0.07^ *d* ^5.10^) and 49.75% Co2 (*sp* ^0.07^ *d* ^5.55^). The out-of-plane π bond arises from d_*xz*_ overlap (51.04% Co1 (*pd* ^99.99^) + 48.96% Co2 (*pd* ^75.30^)), while the in-plane π orbital is slightly polarized (56.89% Co1 (*sp* ^1.39^ *d* ^99.99^) + 43.11% Co2 (*pd* ^2.37^)). A Wiberg bond index of 1.69 indicates a hemibond character. The fMO occupation (σ^2^π^4^δ ^2^σ*π*^2^δ*^2^) corresponds to a formal Co–Co bond order of 1.5. In comparison, the trigonal-type Co_2_(amidinato)_3_ complexes **B**, which also possess a sextet ground state, exhibit an fMO configuration of σ^2^π^4^π*^4^σ*δ ^2^δ*^2^, thereby corresponding to a formal Co–Co bond order of 0.5.^7l^ The Co–Co bond lengths in **B** are significantly longer (2.320–2.385 Å),^7f–7h^ primarily due to the presence of two additional electrons in the π* orbitals, which weaken the metal–metal interaction and result in bond elongation. In contrast, the fMOs of **3** (Figures 4c, S30 and S31) display five α-spin bonding–antibonding pairs derived from the 10 *d* orbitals, but feature an additional β π* orbital from *d*_z2_–*d*_z2_ overlap, reflecting further weakening of the metal–metal bond in the dianionic complex.

Cotton reported that oxidation of the lantern-type amidinate-supported diamagnetic dicobalt complex Co_2_(DPhBz)_4_ (DPhBz = diphenylbenzamidinato) to [Co_2_(DPhBz)_4_]^+^ led to a shortening of the Co–Co bond from 2.3735(9) Å to 2.322(2) Å and 2.332(2) Å,^7i^ accompanied by an increase in the formal Co–Co bond order from 1.0 to 1.5. Given that the ground-state electronic structures of Co_2_(DPhBz)_4_ and [Co_2_(DPhBz)_4_]^+^ are σ^2^π^4^δ ^2^δ*^2^π*^4^ and σ^2^π^4^δ ^2^π*^4^δ*, respectively, removal of an electron from a δ* has a minimal effect on the Co–Co bond length. Instead, the overall contraction appears to be dominated by an increased electrostatic attraction resulting from the higher positive charge on the metal centers. In contrast, the change in Co–Co bond length between compounds **1** and **2** is much less pronounced. Based on their electronic structures, compound **2** contains an electron in the σ* orbital, which would be expected to significantly weaken and lengthen the Co–Co bond. However, this effect is offset by a reduction in intermetallic repulsion, resulting in only a minor change in bond length.

NBO analysis of **3** (Figures S38–S40) corroborates the fMO picture, revealing two β-orbital bonding interactions: a σ bond (0.83 e; 43.8% Co1 (*sp* ^0.82^ *d* ^23.13^) + 56.2% Co2 (*sp* ^0.07^ *d* ^4.76^)) and a π bond (0.93; 47.3% Co1 (*pd* ^10.24^) + 52.7% Co2 (*pd* ^99.99^)). The Wiberg bond index for Co–Co in **3** is 1.18, the lowest of the series—consistent with single-bond character. The fMO-derived electron configuration (σ^2^π^4^δ ^3^σ*π*^3^δ*^3^) corresponds to a formal bond order of one. By contrast, low-coordinate (CN ≤ 3) homounivalent paddlewheel complexes typically maximize metal–metal bond order,^1b,1c^ especially those featuring Co_2_^2+^ cores.^7a,7j^ Here, however, the Co_2_^2+^ unit in **3** exhibits the smallest Co–Co bond order, whereas the more oxidized Co_2_^4+^ center in **1** supports a true Co=Co double bond—inverting the usual trend.

## Conclusions

In summary, we have synthesized and structurally characterized three novel digonal paddlewheel dicobalt complexes **1**–**3**, supported by two diamido ligands. They can be readily interconverted by redox reactions. Structurally, their cobalt-containing fragments are almost superimposed, and their Co_2_N_4_Si_2_ cores exhibit a chairlike conformation. Thus, compounds **1**–**3** are rare examples where different oxidation state Co_2_ units can be accommodated in the same conformation. Unlike the short Co–Co bonds (i.e., double bonds) observed in the homounivalent dicobalt complexes supported by σ-donating ligands, short Co–Co bonds were found in **1** and **2** rather than **3**, resulting from the involvement of more *d* orbitals in Co–N π bonding. The reactivity of **1**–**3** toward small molecules is currently investigated in our laboratory.

## Experiment Section

### General Consideration

All manipulations were carried out using standard Schlenk and glovebox techniques under an atmosphere of high-purity nitrogen. The 4 Å sieves and *Celite* were dried in vacuo for 1 week at a temperature just above 200 °C. Diethyl ether (Et_2_O) and tetrahydrofuran (THF) were distilled under nitrogen from purple sodium benzophenone ketyl. *n*-Hexane and toluene were passed through columns of solvent purification systems (Vigor VAPA-5) to remove oxygen and moisture. Distilled solvents were transferred under vacuum into vacuum-tight glass vessels before being transferred into a glovebox and stored over activated molecular sieves. Solution NMR spectra were recorded by using Varian Unity INOVA 500 MHz and JEOL ECZ500R/S1 500 MHz spectrometers at room temperature. ^1^H and ^13^C­{^1^H} chemical shifts are reported referenced to the residual C_6_D_6_ solvent resonances of 7.16 ppm (^1^H) and 128.6­(t) ppm (^13^C), respectively. Elemental analysis was carried out with a Heraeus CHN-O rapid elementary analyzer. Magnetic measurements for **1**–**3** were conducted between 2 and 300 K with a Quantum Design MPMS-3 SQUID Magnetometer, and data were fitted by the PHI code. The silyldiamide ligand H_2_[Ph_2_Si­(N-2,6-^*i*^Pr_2_C_6_H_3_)_2_][Bibr ref16] and KC_8_ ^17^ were synthesized following the documented methods.

### Synthesis of Li_2_[Ph_2_Si­(N-2,6-^i^Pr_2_C_6_H_3_)_2_][Bibr ref18]

In a glovebox, a 20 mL vial was charged with H_2_[Ph_2_Si­(N-2,6-^*i*^Pr_2_C_6_H_3_)_2_] (1.004 g, 1.880 mmol) and 4 mL of *n*-hexane, Meanwhile, *n*-BuLi (2.5 M in *n*-hexane, 2.5 mL, 6.250 mmol) was precooled at −35 °C. The cold *n*-BuLi solution was then slowly added to the vial. The suspension remained white and was allowed to stir for 2 h. At this point, the white solid was collected by filtration, washed with *n*-hexane, and dried under vacuum to give the product (0.812 g, 1.481 mmol, 78.7% yield). ^1^H NMR (500 MHz, 293 K, C_6_D_6_): δ 0.39 (d, *J*_H–H_ = 6.3 Hz, 6H, HC*Me*_2_), 0.72 (d, *J*_H–H_ = 6.9 Hz, 6H, HC*Me*_2_), 1.04 (d, *J*_H–H_ = 6.5 Hz, 6H, HC*Me*_2_), 1.59 (d, 6H, HC*Me*_2_, *J*_H–H_ = 7.0 Hz), 3.18 (septet, 2H, *H*CMe_2_, *J*_H–H_ = 6.8 Hz), 4.00 (septet, *J*_H–H_ = 6.8 Hz, 2H, *H*CMe_2_), 6.09–7.14 (m, 16H, *Ar*). ^13^C­{^1^H} NMR (126 MHz, 293 K, C_6_D_6_,): δ 24.2 (HC*Me*_2_), 25.3 (HC*Me*_2_), 26.8 (HC*Me*_2_), 27.1 (HC*Me*_2_), 28.2 (H*C*Me_2_), 29.5 (H*C*Me_2_), 118.9 (*Ar*), 124.5 (*Ar*), 125.4 (*Ar*), 126.9 (*Ar*), 127.5 (*Ar*) 135.9 (*Ar*), 141.6 (*Ar*), 143.0 (*Ar*), 146.4 (*Ar*), 149.0 (*Ar*), 153.4 (*Ar*)

**Caution!** *n*-butyllithium and Li_2_[Ph_2_Si­(N-2,6-^*i*^Pr_2_C_6_H_3_)_2_] are pyrophoric and must be handled with extreme care.

### Synthesis of Co_2_(μκ^2^-Ph_2_Si­(N-2,6-^i^Pr_2_C_6_H_3_)_2_)_2_ (**1**)

In a glovebox, a 20 mL vial was charged with Li_2_[Ph_2_Si­(N-2,6-^*i*^Pr_2_C_6_H_3_)_2_] (0.360 g, 0.659 mmol) and CoCl_2_ (0.078 g, 0.60 mmol). Four ml THF was added to the mixture, and the resultant suspension quickly turned dark brown. The solution was allowed to stir for 1 day. At this point, all volatiles were removed under reduced pressure. The dark brown residue was collected by filtration and washed with Et_2_O and then followed by toluene. The remaining material was extracted by THF and recrystallized in THF to give green solid **1** (0.029 g, 0.025 mmol, 8.3% yield). Single crystals suitable for X-ray diffraction were obtained from recrystallization in THF at −35 °C. μ_eff_ = 4.71 μ_B_ (Evans method, 293 K). μ_eff_ = 5.18 μ_B_ (SQUID, 300 K). Anal. Calcd for C_72_H_88_Co_2_N_4_Si_2_: C, 73.07; H, 7.49; N, 4.73. Found: C, 73.05; H, 8.03; N, 4.47.

No uncommon hazards are noted.

### Synthesis of [Li­(OEt_2_)_4_]­[Co_2_(μκ^2^-Ph_2_Si­(N-2,6-^i^Pr_2_C_6_H_3_)_2_)_2_] ([Li­(OEt_2_)_4_]­[**2**])

#### Method **1**

In a glovebox, a 20 mL vial was charged with Li_2_[Ph_2_Si­(N-2,6-^*i*^Pr_2_C_6_H_3_)_2_] (0.360 g, 0.659 mmol) and CoCl_2_ (0.078 g, 0.60 mmol). Four ml THF was added to the mixture, and the resultant suspension quickly turned dark brown. The solution was allowed to stir for 1 h. At this point, all volatiles were removed under reduced pressure. The dark residue was washed with *n*-hexane, then extracted with Et_2_O, and the insoluble materials were filtered off through a pad of *Celite*. The filtrate was then dried under vacuum to give­[Li­(OEt_2_)_4_]­[**2**] (0.067 g, 0.045 mmol, 15.3% yield). Single crystals suitable for X-ray diffraction of [Li­(OEt_2_)_4_]­[**2**] were obtained from recrystallization in Et_2_O at – 35 °C. μ_eff_ = 5.84 μ_B_ (Evans method, 293 K). μ_eff_ = 6.21 μ_B_ (SQUID, 300 K). Anal. Calcd for C_88_H_128_Co_2_LiN_4_O_4_Si_2_: C, 71.08; H, 8.68; N, 3.77. Found: C, 70.46; H, 7.99; N, 4.03.

No uncommon hazards are noted.

#### Method **2**

In a glovebox, a 20 mL vial was charged with Li_2_[Ph_2_Si­(N-2,6-^*i*^Pr_2_C_6_H_3_)_2_] (0.324 g, 0.591 mmol), CoCl_2_ (0.078 g, 0.600 mmol), and KC_8_ (0.041 g, 0.297 mmol). Four ml THF was added to the mixture, and the resultant suspension quickly turned dark purple. The solution was allowed to stir for 1 h. At this point, all volatiles were removed under reduced pressure. The dark purple residue was collected by filtration and washed with *n*-hexane, then extracted with Et_2_O, and the insoluble materials were filtered off through a pad of *Celite*. The filtrate was then dried under vacuum to give **2** (0.262 g, 0.176 mmol, 59.5% yield).

#### Method **3**

In a glovebox, a 20 mL vial was charged with **1** (0.046 g, 0.038 mmol) and [Li]_2_[**3**] (0.043 g, 0.036 mmol). Four ml THF was added to the mixture, and the resultant suspension quickly turned dark purple. The solution was allowed to stir for 1 h. At this point, all volatiles were removed under reduced pressure. The dark purple residue was collected by filtration and washed with *n*-hexane, then extracted with Et_2_O, and the insoluble materials were filtered off through a pad of *Celite*. The filtrate was then dried under vacuum to give [Li­(OEt_2_)_4_]­[**2**] (0.033 g, 0.022 mmol, 30.6% yield).

### Synthesis of Li_2_[Co_2_(μκ^2^-Ph_2_Si­(N-2,6-^i^Pr_2_C_6_H_3_)_2_)_2_] ([Li]_2_[**3**])

In a glovebox, a 20 mL vial was charged with Li_2_[Ph_2_Si­(N-2,6-^*i*^Pr_2_C_6_H_3_)_2_] (0.237 g, 0.432 mmol), CoCl_2_ (0.057 g, 0.439 mmol), and KC_8_ (0.0656 g, 0.475 mmol). Four ml THF was added to the mixture, and the resultant suspension quickly turned dark brown. The solution was allowed to stir for 1 h. At this point, all volatiles were removed under reduced pressure. The dark brown residue was washed with *n*-hexane and extracted with Et_2_O, and the insoluble materials were filtered off through a pad of *Celite*. The filtrate was then dried under vacuum to give an orange brown solid of [Li]_2_[**3**] (0.243 g, 0.203 mmol, 92.4% yield). μ_eff_ = 4.89 μ_B_ (Evans method, 293 K). μ_eff_ = 5.68 μ_B_ (SQUID, 300 K). Anal. Calcd for C_72_H_88_Co_2_Li_2_N_4_Si_2_: C, 72.22; H, 7.41; N, 4.68. Found: C, 72.31.; H, 7.79; N, 4.03.

No uncommon hazards are noted.

### Synthesis of [(THF)_2_K⊂(18-crown-6)]_2_[Co_2_(μκ^2^-Ph_2_Si­(N-2,6-^i^Pr_2_C_6_H_3_)_2_)_2_] ([K-(18-C-6)]_2_[**3**])

In a glovebox, a 20 mL vial was charged with [Li]_2_[**3**] (0.132g, 0.110 mmol), KI (0.042 g, 0.253 mmol), and 18-crown-6 (0.030 g, 0.114 mmol). Four ml Et_2_O was added to the mixture, and the resultant suspension quickly turned dark brown. The solution was allowed to stir for 1 day. At this point, all volatiles were removed under reduced pressure. The dark brown residue was washed with Et_2_O and extracted into THF, and the insoluble materials were filtered off through a pad of *Celite*. The filtrate was then dried under vacuum to give the orange brown solid of [K-(18-C-6)]_2_[**3**] (0.036 g, 0.020 mmol, 18.1% yield). Single crystals suitable for X-ray diffraction of [K-(18-C-6)]_2_[**3**] were obtained from recrystallization in THF at −35 °C. Anal. Calcd for C_96_H_136_Co_2_K_2_N_4_O_12_Si_2_: C, 64.40; H, 7.66; N, 3.13. Found: C, 65.10.; H, 8.02; N, 3.10.

No uncommon hazards are noted.

## Supplementary Material



## Notes

General considerations; details of crystal data; NMR spectra; elemental analysis report; Evans method; SQUID; cyclic voltammetry; and details of computational studies (DOCX)

## ∥

Merck Life Science, Bengaluru, Karnataka, India
