# Supplementary material for: Paddlewheel Dicobalt Complexes with Metal Centers Having Various Oxidation States
Source: Inorg Chem. 2025 Sep 24;64(39):19555–62. doi: 10.1021/acs.inorgchem.5c02468 (PMC12505273; doi:10.1021/acs.inorgchem.5c02468)
Supplement: Supplementary file 1 [file ic5c02468_si_001.docx]

**Supporting Information**

**Paddlewheel Dicobalt Complexes with Metal Centers Having Various Oxidation States**

Bo-An Liao^†^, Cian-Wei Yang^†^, Anokh K. Nair, Yi-Chou Tsai*

*^a^*Department of Chemistry, National Tsing Hua University, Hsinchu, 30013, Taiwan

Email: yictsai@mx.nthu.edu.tw (Yi-Chou Tsai)

† The authors contributed equally to this work.

**Table of contents**

[1. General considerations 1](#_Toc204678576)

[2. Details of crystal data 4](#_Toc204678577)

[3. NMR spectra 30](#_Toc204678578)

[4. Elemental analysis report 33](#_Toc204678579)

[5. Evans method 37](#_Toc204678580)

[6. SQUID 39](#_Toc204678581)

[7. Cyclic Voltammetry 42](#_Toc204678582)

[8. Details of computational studies 43](#_Toc204678583)

[9. References 113](#_Toc204678584)

1. General considerations

All manipulations were carried out using standard Schlenk and glove box techniques under an atmosphere of high-purity nitrogen. The 4 Å sieves and *Celite* were dried in vacuo one week at a temperature just above 200 °C. Diethyl ether (Et_2_O) and tetrahydrofuran (THF) were distilled under nitrogen from purple sodium benzophenone ketyl. *n*-Hexane and toluene were passed through columns of solvent purification systems (Vigor VAPA-5) to remove oxygen and moisture. Distilled solvents were transferred under vacuum into vacuum-tight glass vessels before being transferred into a glove box and stored over activated molecular sieves. Solution NMR spectra were recorded using Varian Unity INOVA 500 MHz and JEOL ECZ500R/S1 500 MHz spectrometers at room temperature. ^1^H and ^13^C{^1^H} chemical shift are reported referenced to the residual C_6_D_6_ solvent resonances of 7.16 ppm (^1^H) and 128.6(t) ppm (^13^C) respectively. Elemental analysis was carried out with a Heraeus CHN-O rapid elementary analyzer. Magnetic measurements for **1**−**3** were conducted between 2 to 300 K with a Quantum Design MPMS-3 SQUID Magnetometer and data were fitted by PHI.^1^ The silyldiamide ligand H_2_[Ph_2_Si(N-2,6-*^i^*Pr_2_C_6_H_3_)_2_]^2^ and KC_8_^3^ were synthesized following the documented methods.

**SQUID measurements**

Magnetic measurements for **1**−**3** were conducted between 2 to 300 K with a Quantum Design MPMS-3 SQUID Magnetometer. The sample was loaded into a capsule, hermetically sealed under an inert atmosphere, and mounted in a non-magnetic straw. ZFC magnetization data were then collected under an applied dc field of 5000 or 10000 G. After cooling the sealed capsule to 2 K in zero field, magnetization was recorded on warming at the following temperature intervals: 1 K steps (2–10 K); 2 K steps (12–50 K); 5 K steps (55–100 K); 10 K steps (110–300 K). Magnetization data were fitted with PHI using the Hamiltonian below and corrected with temperature independent paramagnetism (TIP) = 830 × 10^–6^ cm^3^ mol^–1^ and intermolecular interaction (z*J*).

$$\hat{H}=\sum_{i=1}^{2} \left\{ D_{i}\left[ {\hat{S}_{iz}}^{2}-\frac{1}{3}\hat{S}_{i}\left( \hat{S}_{i}+1 \right) \right]+E_{i}\left( {\hat{S}_{ix}}^{2}-{\hat{S}_{iy}}^{2} \right) \right\}+\mu_{B}\sum_{i=1}^{2} {g\hat{S}}_{i}\cdot B$$

Effective magnetic moments (*μ*_eff_) were calculated and then corrected for sample’s diamagnetism using diamagnetic constants derived from Pascal’s constants.^5^

**Evans method**

Effective magnetic moments (*μ*_eff_) of **1**−**3** were determined by Evans method.^5−7^ **1** was dissolved in the mixture of THF and TMS_2_O, and ^1^H NMR spectrum was measured at 293 K by No-D technique with the mixture of THF and TMS_2_O as internal standard.^8−9^ **2** and **3** was dissolved in the mixture of C_6_D_6_ and TMS_2_O, and ^1^H NMR spectra were measured at 293 K with the mixture of C_6_D_6_ and TMS_2_O as internal standard. Effective magnetic moments were determined by following equation, and diamagnetic constants were calculated by Pascal’s constants.^10^

$$x_{g}=\frac{3\Delta f}{4\pi fm}+\frac{x_{0}\left( D_{solvent}-D_{mix} \right)}{m}+x_{0}$$

$$x_{m}=x_{g}\left( MW \right)-x_{d}$$

$$\mu_{eff}=\sqrt{8x_{m}T}$$

*∆f*: chemical shift difference; *f*: operating frequency of ^1^H NMR; *m*: concentration of sample in solvent (g/mL); *χ_0_*: mass susceptibility of solvent; *χ_d_*: diamagnetic correction of sample.

**X-ray crystallography**

Single crystals data were collected on Rigaku XtaLAB HyPix-Arc 150 diffractometer with Cu-*Kα* radiation (*λ* = 1.54178 Å) or Mo-*Kα* radiation (*λ* = 0.71073 Å) at 100(10) K. The structure determinations and refinements were carried out using the *SHELXS*^11^ and *SHELXL*^12^ programs respectively on the Olex2 interface.^13^ The structures were solved using direct methods, which yielded the positions of all nonhydrogen atoms. Hydrogen atoms were placed in calculated positions in the final structure refinement. Crystallographic refinement parameters are listed in Table *S1*.

**Cyclic Voltammetry**

Cyclic-voltammetry measurements were performed on a PalmSens4 potentiostat controlled by PSTrace software, using a conventional three-electrode cell. A 3.0 mm-diameter glassy-carbon disk served as the working electrode, and platinum wires were employed as the counter and reference electrodes. A 0.10 M solution of [Bu_4_N][PF_6_] was used as the supporting electrolyte. The glassy-carbon working electrode was repolished before each voltammogram on a wetted Buehler felt pad with 0.05 μm alumina slurry. All potentials are reported versus the ferrocene/ferrocenium couple (Fc/Fc⁺) as an internal reference.

**Open circuit potential**

Open circuit potential measurement was performed on a PalmSens4 potentiostat controlled by PSTrace software, using a conventional three-electrode cell. platinum wires were employed as the working and counter electrode, and an Ag wire was employed as reference electrodes. The potential is reported versus the ferrocene/ferrocenium couple (Fc/Fc⁺) as an internal reference.

2. Details of crystal data

**Table *S*1.** Crystal and intensity collection data for **1**, **2**[Li(OEt_2_)_4_] and **3**[K-(18-C-6)]_2_

| Complex | 1 | 2[Li(OEt_2_)_4_] | | 3[K-(18-C-6)]_2_ | |  |
| --- | --- | --- | --- | --- | --- | --- |
| Empirical formula | C_72_H_88_Co_2_N_4_Si_2_ | C_88_H_123.34_Co_2_LiN_4_O_4_Si_2_ | | C_112_H_168_Co_2_K_2_N_4_O_16_Si_2_ | |  |
| Crystal system | triclinic | triclinic | | monoclinic | |  |
| Formula weight | 1183.50 | 1482.22 | | 2078.73 | |  |
| Space group | P-1 | P-1 | | C2/c | |  |
| *a*, Å | 12.5789(7) | 11.97970(18) | | 32.4277(6) | |  |
| *b*, Å | 12.8115(7) | 13.25783(19) | | 19.3978(4) | |  |
| *c*, Å | 15.5797(10) | 27.6560(5) | | 19.7244(3) | |  |
| *α*, deg | 80.781(3) | 82.5774(14) | | 90 | |  |
| *β*, deg | 85.991(3) | 85.4254(14) | | 106.120(2) | |  |
| *γ*, deg | 69.149(3) | 84.6812(12) | | 90 | |  |
| *V*, Å^3^ | 2315.8(2) | 4326.67(13) | | 32.4277(6) | |  |
| *Z* | 1 | 2 | | 4 | |  |
| Cryst dimens, mm^3^ | 0.22 × 0.2 × 0.2 | 0.183 × 0.112 × 0.045 | | 0.08 × 0.06 × 0.02 | |  |
| radiation wavelength: γ, | 0.71073 (Mo *Kα*) | | 1.54184 (Cu *Kα*) | | 1.54184 (Cu *Kα*) | |
| 2*θ* rang, deg | 3.438 to 52.976 | 1.488 to 52.896 | | 5.366 to 134.158 | |  |
| Limiting indices | -15<=h<=15 | -14 ≤ h ≤ 14 | | -37<= h<=38 | |  |
|  | -15<=k<=16 | -15 ≤ k ≤ 16 | | -37 <=h<=38 | |  |
|  | -16<=l<=19 | -34 ≤ l ≤ 33 | | -23<= l <=20 | |  |
| Reflections collected | 30895 | 67640 | | 54394 | |  |
| Independent reflections | 9398 [R(int) = 0.0377] | 16759 [R(int) = 0.0256] | | 10639 [R(int) = 0.0545] | |  |
| Data / restraints / parameters | 9398 / 0 / 369 | 16759/681/1147 | | 10639 / 0 / 630 | |  |
| GOF on *F*^2^ | 1.082 | 1.059 | | 1.074 | |  |
| Final R indices  [I＞2*σ*(I)] | R_1_ = 0.0574,  wR_2_ = 0.1670 | R_1_ = 0.0459,  wR_2_ = 0.1198 | | R_1_ = 0.0565,  wR_2_ = 0.1585 | |  |
| R indices (all data) | R_1_ = 0.0714,  wR_2_ = 0.1748 | R_1_ = 0.0583,  wR_2_ = 0.1262 | | R_1_ = 0.0565,  wR_2_ = 0.1585 | |  |
| Largest diff. peak and hole | 0.60 and -0.47 e.Å^-3^ | 0.70 and -0.35 e.Å^-3^ | | 0.58 and -0.48 e.Å^-3^ | |  |


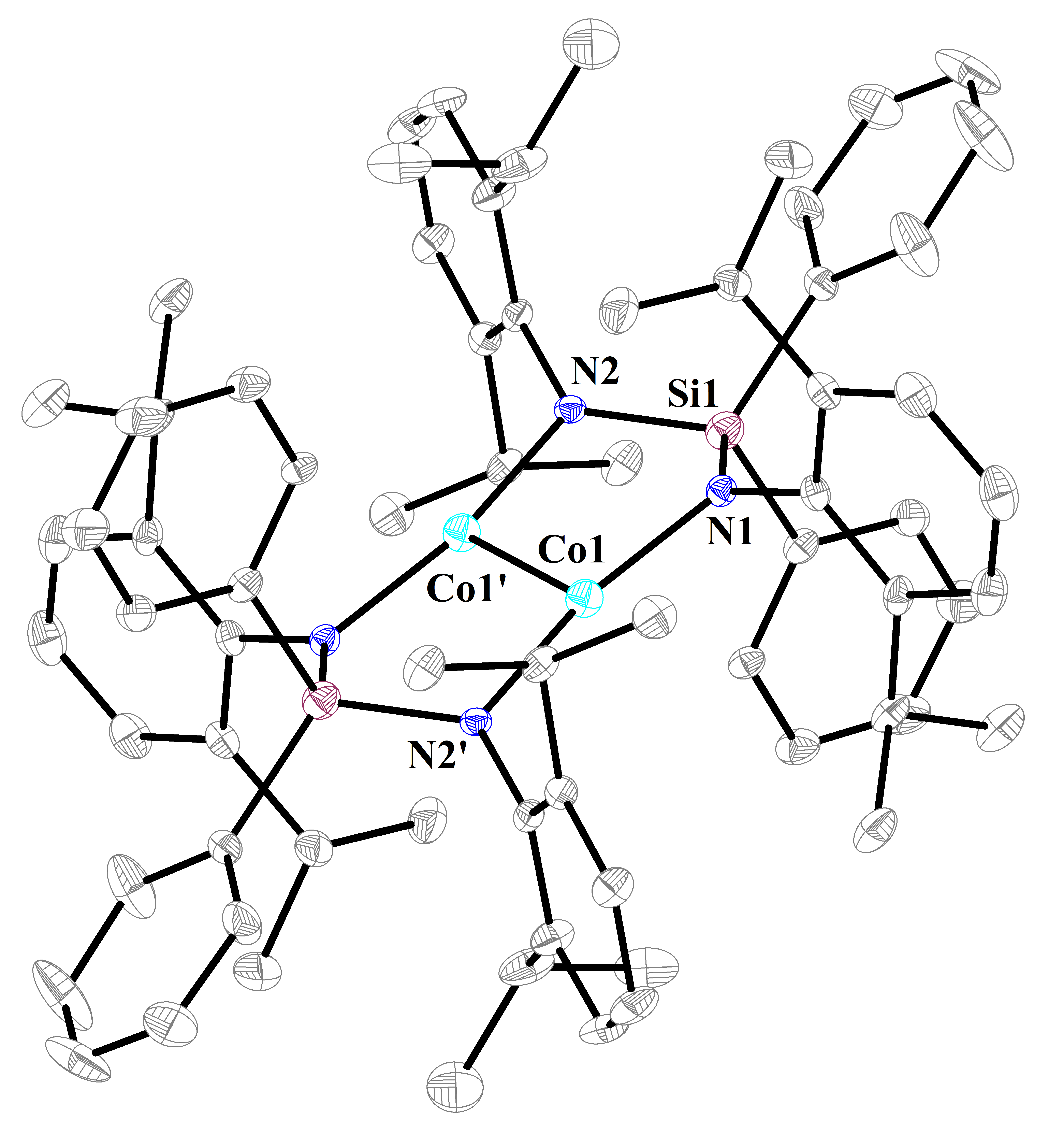


**Figure. *S*1** The solid-state molecular structure of **1** drawn with 30% thermal ellipsoids probability level. Hydrogen atoms are omitted for the sake of clarity. Symmetry-generated atoms are labeled by the ' sign. Selected bond lengths (Å) and angles (°): Co(1)−Co(1') 2.2390(5), Co(1)−N(1) 1.878(3), N(1)−Si(1) 1.719(3), Si(1)−N(2) 1.726(3), Co(1')−N(2) 1.862(3); N(1)−Co(1)−Co(1') 99.62(8), Si(1)−N(1)−Co(1) 111.49(13), N(1)−Si(1)−N(2) 105.70(12), Si(1)−N(2)−Co(1') 114.63(12), N(2)−Co(1')−Co(1), 95.95(8); N(1)−Co(1)−Co(1')−N(2) 0.33(11), Si(1)−N(2)−N(1)−Co(1) 146.76(16).


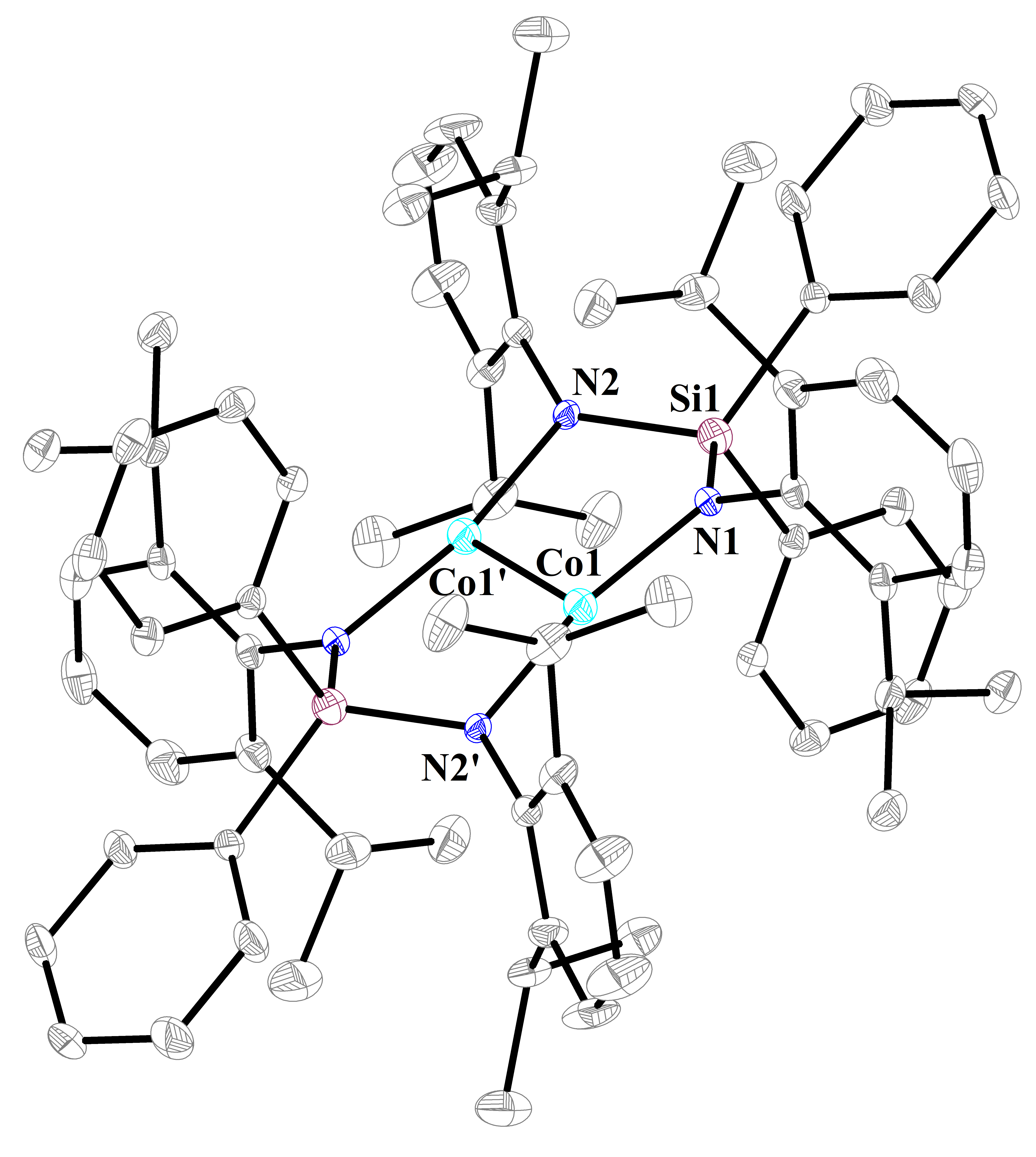


**Figure. *S*2** The solid-state molecular structure of **2**[Li(OEt_2_)_4_] drawn with 30% thermal ellipsoids probability level. Hydrogen atoms and countercation are omitted for the sake of clarity. Symmetry-generated atoms are labeled by the ' sign. Selected bond lengths (Å) and angles (°): **Form1**: Co(1)−Co(1') 2.2376(8), Co(1)−N(1) 1.8757(19), N(1)−Si(1) 1.718(2), Si(1)−N(2) 1.7241(19), Co(1')−N(2) 1.8535(19); N(1)−Co(1)−Co(1') 99.19(7), Si(1)−N(1)−Co(1) 112.12(10), N(1)−Si(1)−N(2) 105.42(10), Si(1)−N(2)−Co(1') 115.06(10); N(2)−Co(1')−Co(1) 96.22(6), N(1)−Co(1)−Co(1')−N(2) 0.22(9), Si(1)−N(2)−N(1)−Co(1), 147.67(12). **Form2**: Co(1)−Co(1') 2.2327(8), Co(1)−N(1) 1.8748(16), N(1)−Si(1) 1.7167(18), Si(1)−N(2) 1.7174(18), Co(1')−N(2) 1.8582(16); N(1)−Co(1)−Co(1') 98.98(6), Si(1)−N(1)−Co(1) 112.65(9), N(1)−Si(1)−N(2) 105.23(9), Si(1)−N(2)−Co(1') 115.38(9); N(2)−Co(1')−Co(1) 96.28(8), N(1)−Co(1)−Co(1') −N(2) 0.09(9), Si(1)−N(2)−N(1)−Co(1), 148.34(12).
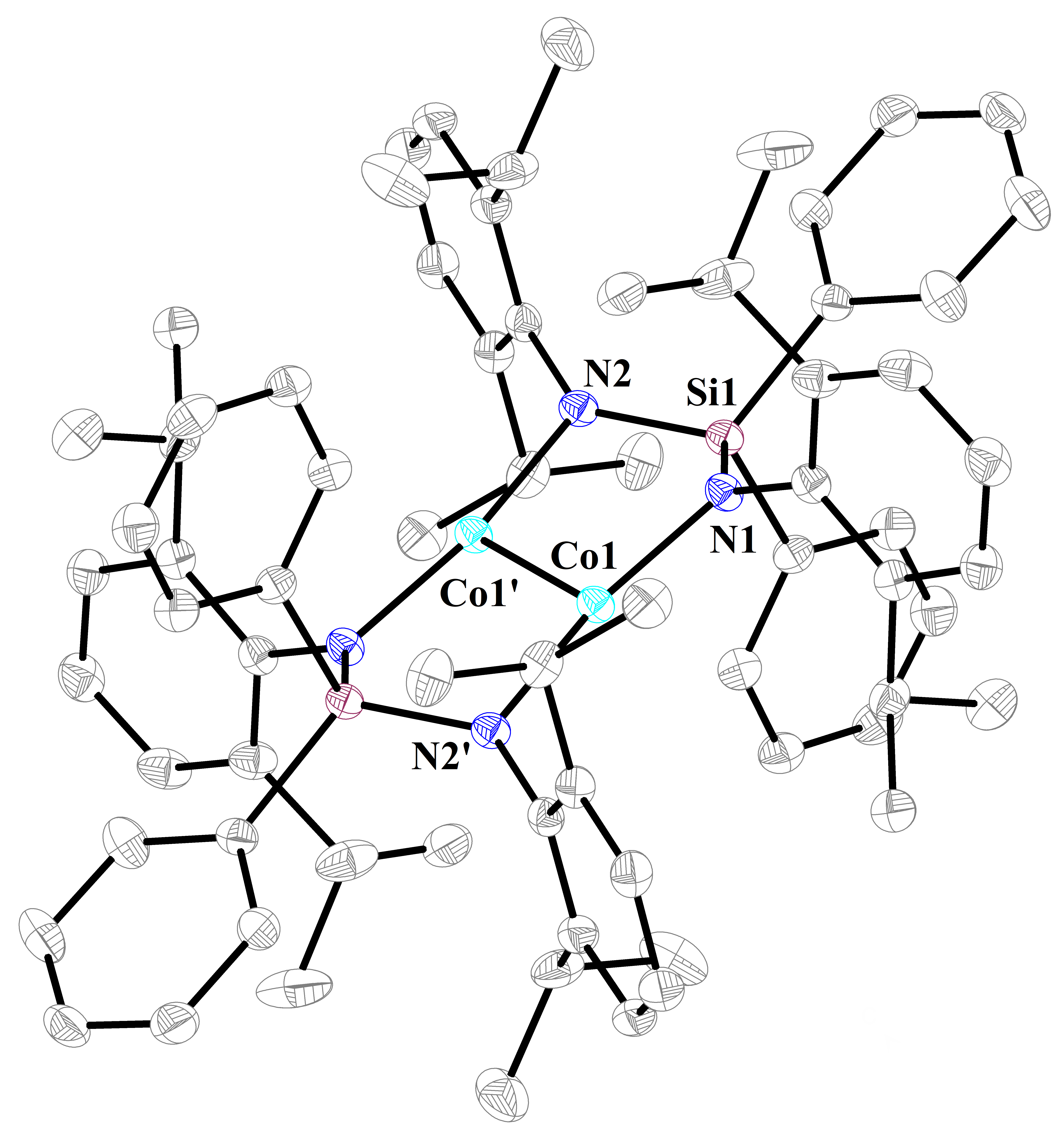


**Figure. *S*3** The solid-state molecular structure of **3**[K-(18-C-6)]_2_ drawn with 30% thermal ellipsoids probability level. Hydrogen atoms and countercations are omitted for the sake of clarity. Symmetry-generated atoms are labeled by the ' sign. Selected bond lengths (Å) and angles (°): Co(1)−Co(1') 2.3276(8), Co(1)−N(1) 1.888(2), N(1)−Si(1) 1.706(2), Si(1)−N(2) 1.699(2), Co(1')−N(2) 1.896(2); N(1)−Co(1)−Co(1') 96.06(7), Si(1)−N(1)−Co(1) 111.48(12), N(1)−Si(1)−N(2) 108.97(11), Si(1)−N(2)−Co(1') 111.20(12), N(2)−Co(1')−Co(1) 97.41(7); N(1)−Co(1)−Co(1') −N(2) 0.15 (10), Si(1)−N(2)−N(1)−Co(1), 141.51(15).

**Table *S*2.** Fractional atomic coordinates (×10^4^) and equivalent isotropic displacement parameters (Å^2^×10^3^) for **1**. U_eq_ is defined as one third of the trace of the orthogonalised U_IJ_ tensor.

| **Atom** | ***x*** | ***y*** | ***z*** | **U(eq)** |
| --- | --- | --- | --- | --- |
| Co1 | 537.7(3) | 4152.3(3) | 10334.3(3) | 16.38(13) |
| N1 | 1245(2) | 3596(2) | 9328.3(17) | 18.3(5) |
| N2 | -67(2) | 5678(2) | 8509.5(17) | 18.8(5) |
| Si1 | 1231.2(7) | 4619.4(6) | 8456.8(5) | 16.96(19) |
| C1 | 1761(2) | 2412(2) | 9390(2) | 20.2(6) |
| C2 | 2800(3) | 1827(2) | 9818(2) | 21.6(6) |
| C3 | 3288(3) | 661(3) | 9863(2) | 29.0(8) |
| C4 | 2769(3) | 49(3) | 9500(3) | 36.7(9) |
| C5 | 1742(3) | 614(3) | 9090(3) | 36.1(9) |
| C6 | 1221(3) | 1777(3) | 9025(2) | 30.7(8) |
| C7 | 65(3) | 2348(3) | 8602(3) | 40.7(10) |
| C8 | 22(4) | 1999(4) | 7729(3) | 57.8(12) |
| C9 | -874(3) | 2157(4) | 9200(3) | 48.9(11) |
| C10 | -689(2) | 6556(3) | 7856(2) | 19.8(6) |
| C11 | -1465(3) | 6355(3) | 7332(2) | 24.7(7) |
| C12 | -2067(3) | 7222(3) | 6701(2) | 35.3(8) |
| C13 | -1942(3) | 8263(3) | 6558(3) | 41.8(10) |
| C14 | -1213(3) | 8460(3) | 7071(2) | 35.1(8) |
| C15 | -591(3) | 7640(3) | 7726(2) | 24.1(7) |
| C16 | -1672(3) | 5245(3) | 7478(2) | 28.9(7) |
| C17 | -1944(5) | 4913(4) | 6656(3) | 61.2(14) |
| C18 | -2624(4) | 5287(3) | 8138(3) | 42.8(10) |
| C19 | 143(3) | 7967(3) | 8297(2) | 28.8(7) |
| C20 | 1176(3) | 8138(3) | 7821(3) | 40.7(10) |
| C21 | -552(3) | 9008(3) | 8709(3) | 38.8(9) |
| C22 | 3421(3) | 2445(3) | 10235(2) | 24.1(7) |
| C23 | 4424(3) | 2549(3) | 9648(3) | 33.5(8) |
| C24 | 3814(3) | 1895(3) | 11155(2) | 36.2(8) |
| C25 | 1587(3) | 4026(3) | 7394(2) | 22.6(6) |
| C26 | 2448(4) | 2982(3) | 7350(3) | 40.2(9) |
| C27 | 2790(4) | 2551(4) | 6598(3) | 54.4(12) |
| C28 | 2254(4) | 3133(5) | 5834(3) | 61.1(14) |
| C29 | 1390(4) | 4095(5) | 5846(3) | 70.3(17) |
| C30 | 1068(4) | 4578(4) | 6610(3) | 54.7(13) |
| C31 | 2440(3) | 5159(3) | 8569(2) | 22.9(7) |
| C32 | 3257(3) | 5158(3) | 7918(3) | 32.6(8) |
| C33 | 4153(3) | 5515(4) | 8032(3) | 43.8(10) |
| C34 | 4253(3) | 5875(3) | 8792(3) | 42.2(10) |
| C35 | 3450(3) | 5903(3) | 9446(3) | 31.9(8) |
| C36 | 2550(3) | 5546(2) | 9334(2) | 22.4(7) |

**Table *S*3.** Anisotropic displacement parameters (Å^2^×10^3^) for **1**. The anisotropic displacement factor exponent takes the form: -2π^2^[h^2^a^*2^U_11_ + 2hka*b*U_12_ +…].

| Atom | **U_11_** | **U_22_** | **U_33_** | **U_23_** | **U_13_** | **U_12_** |
| --- | --- | --- | --- | --- | --- | --- |
| Co1 | 15.2(2) | 14.6(2) | 17.9(2) | -2.16(15) | 0.54(15) | -3.83(15) |
| N1 | 19.0(12) | 16.4(12) | 20.2(14) | -5.6(10) | 1.5(10) | -6.1(10) |
| N2 | 17.9(12) | 18.2(12) | 20.4(14) | -2.5(10) | 0.9(10) | -6.6(10) |
| Si1 | 15.6(4) | 15.9(4) | 19.4(4) | -2.6(3) | 0.5(3) | -5.7(3) |
| C1 | 19.5(14) | 17.7(14) | 23.6(17) | -2.7(12) | 2.6(12) | -7.3(12) |
| C2 | 19.3(15) | 19.3(15) | 24.3(17) | -5.0(12) | 4.5(12) | -4.8(12) |
| C3 | 21.7(16) | 19.8(16) | 40(2) | -3.1(14) | -0.4(14) | -1.2(13) |
| C4 | 34.1(19) | 14.0(15) | 59(3) | -9.9(15) | 4.3(17) | -4.3(14) |
| C5 | 40(2) | 25.3(17) | 50(2) | -16.5(16) | 0.5(17) | -16.1(16) |
| C6 | 30.9(18) | 24.1(16) | 40(2) | -3.6(15) | -8.6(15) | -11.5(14) |
| C7 | 39(2) | 28.6(18) | 59(3) | -5.8(17) | -22.6(19) | -13.2(16) |
| C8 | 58(3) | 76(3) | 54(3) | -10(2) | -15(2) | -38(3) |
| C9 | 33(2) | 43(2) | 70(3) | -25(2) | -11(2) | -4.7(18) |
| C10 | 15.6(14) | 23.2(15) | 17.9(16) | -1.7(12) | 1.7(11) | -4.3(12) |
| C11 | 23.4(16) | 31.0(17) | 19.0(17) | -4.7(13) | 1.0(12) | -8.3(13) |
| C12 | 32.0(19) | 44(2) | 26.4(19) | -0.9(16) | -8.7(15) | -9.2(16) |
| C13 | 39(2) | 44(2) | 29(2) | 8.6(17) | -4.3(16) | -3.8(17) |
| C14 | 33.7(19) | 29.9(18) | 35(2) | 6.0(15) | -0.8(16) | -7.5(15) |
| C15 | 23.5(16) | 18.3(15) | 24.8(17) | 1.9(12) | 3.3(13) | -3.2(12) |
| C16 | 31.1(18) | 35.1(18) | 24.0(18) | -4.2(14) | -4.5(14) | -14.9(15) |
| C17 | 99(4) | 75(3) | 40(3) | -21(2) | 7(3) | -63(3) |
| C18 | 47(2) | 42(2) | 43(2) | -5.1(18) | 11.9(19) | -23.9(19) |
| C19 | 29.8(17) | 16.6(15) | 40(2) | -0.1(14) | -1.4(15) | -9.1(13) |
| C20 | 30.0(19) | 25.9(18) | 65(3) | -1.3(17) | 0.7(18) | -10.9(15) |
| C21 | 39(2) | 21.1(17) | 55(3) | -0.6(16) | 3.1(18) | -11.5(15) |
| C22 | 19.4(15) | 17.5(14) | 32.8(19) | -0.4(13) | -3.6(13) | -4.2(12) |
| C23 | 22.5(17) | 31.5(18) | 45(2) | -0.1(16) | -0.9(15) | -10.0(14) |
| C24 | 33.5(19) | 36.1(19) | 37(2) | -0.9(16) | -10.5(16) | -10.3(16) |
| C25 | 18.7(15) | 30.1(16) | 21.2(17) | -6.3(13) | 1.6(12) | -10.7(13) |
| C26 | 53(2) | 28.5(18) | 29(2) | -7.7(15) | 12.1(17) | -4.0(17) |
| C27 | 60(3) | 48(3) | 53(3) | -26(2) | 17(2) | -12(2) |
| C28 | 47(3) | 100(4) | 45(3) | -50(3) | 12(2) | -22(3) |
| C29 | 51(3) | 106(4) | 23(2) | -11(2) | -6.4(19) | 12(3) |
| C30 | 42(2) | 61(3) | 31(2) | -1(2) | 4.4(18) | 15(2) |
| C31 | 18.9(15) | 20.1(15) | 29.5(18) | -2.1(13) | -0.5(13) | -7.0(12) |
| C32 | 32.4(18) | 38.4(19) | 35(2) | -14.6(16) | 12.3(15) | -20.0(16) |
| C33 | 34(2) | 56(2) | 55(3) | -25(2) | 21.9(18) | -29.6(19) |
| C34 | 29.3(19) | 50(2) | 61(3) | -25(2) | 10.8(18) | -25.7(18) |
| C35 | 26.1(17) | 36.2(19) | 41(2) | -12.0(16) | 0.8(15) | -17.7(15) |
| C36 | 17.5(14) | 20.8(15) | 30.4(18) | -5.8(13) | 0.6(12) | -7.9(12) |

**Table *S*4.** Hydrogen atom coordinates (Å×10^4^) and isotropic displacement parameters (Å^2^×10^3^) for **1**.

| **Atom** | ***x*** | ***y*** | ***z*** | **U(eq)** |
| --- | --- | --- | --- | --- |
| H3 | 3978.96 | 283.53 | 10142.18 | 35 |
| H4 | 3107.45 | -730.42 | 9532.72 | 44 |
| H5 | 1388.72 | 203.9 | 8850.36 | 43 |
| H7 | -82 | 3161.44 | 8503.5 | 49 |
| H8A | 164.87 | 1202.51 | 7800.09 | 87 |
| H8B | 589.39 | 2170.07 | 7347.39 | 87 |
| H8C | -717.16 | 2402.48 | 7482.94 | 87 |
| H9A | -699.05 | 1361.67 | 9378.07 | 73 |
| H9B | -1582.75 | 2467.42 | 8898.02 | 73 |
| H9C | -933.06 | 2520.69 | 9703.43 | 73 |
| H12 | -2575.88 | 7095.74 | 6361.21 | 42 |
| H13 | -2344.76 | 8820.6 | 6121.14 | 50 |
| H14 | -1131.41 | 9162.88 | 6979.25 | 42 |
| H16 | -976.45 | 4654.63 | 7717.61 | 35 |
| H17A | -2715.84 | 5352.54 | 6497.13 | 92 |
| H17B | -1848.29 | 4126.11 | 6752.26 | 92 |
| H17C | -1439.59 | 5049.62 | 6195.9 | 92 |
| H18A | -3317.48 | 5857.8 | 7915.96 | 64 |
| H18B | -2437.52 | 5463.19 | 8670.97 | 64 |
| H18C | -2721.26 | 4567.17 | 8243.85 | 64 |
| H19 | 423.71 | 7341.18 | 8772.22 | 35 |
| H20A | 1622.53 | 7463.8 | 7585.95 | 61 |
| H20B | 1626.57 | 8299.26 | 8218 | 61 |
| H20C | 931.73 | 8757.26 | 7356.18 | 61 |
| H21A | -864.49 | 9634.16 | 8259.62 | 58 |
| H21B | -70.97 | 9184.76 | 9070.41 | 58 |
| H21C | -1158.84 | 8860.79 | 9054.89 | 58 |
| H22 | 2886.43 | 3210.47 | 10270.57 | 29 |
| H23A | 4974.02 | 1811.15 | 9613.36 | 50 |
| H23B | 4772.36 | 2992.34 | 9888.55 | 50 |
| H23C | 4152.19 | 2910.29 | 9076.89 | 50 |
| H24A | 3172.24 | 1844.86 | 11508.18 | 54 |
| H24B | 4154.85 | 2341.65 | 11397.58 | 54 |
| H24C | 4361.9 | 1150.8 | 11138.78 | 54 |
| H26 | 2802.28 | 2563.15 | 7861.95 | 48 |
| H27 | 3385.34 | 1865.49 | 6596.81 | 65 |
| H28 | 2497.67 | 2850.28 | 5312.98 | 73 |
| H29 | 990.95 | 4454.89 | 5337.82 | 84 |
| H30 | 495.64 | 5281.51 | 6592.71 | 66 |
| H32 | 3202.82 | 4912.9 | 7397.35 | 39 |
| H33 | 4687.43 | 5510.15 | 7588.12 | 53 |
| H34 | 4862.19 | 6100.23 | 8868.24 | 51 |
| H35 | 3508.98 | 6158.66 | 9960.32 | 38 |
| H36 | 2012.26 | 5566.24 | 9778.54 | 27 |

**Table *S*5.** Fractional atomic coordinates (×10^4^) and equivalent isotropic displacement parameters (Å^2^×10^3^) for **2**. U_eq_ is defined as one third of the trace of the orthogonalised U_IJ_ tensor.

| **Atom** | ***x*** | ***y*** | ***z*** | **U(eq)** |
| --- | --- | --- | --- | --- |
| C2 | 3694(2) | 6355.0(17) | 11322.6(8) | 37.7(5) |
| C3 | 2854(2) | 6720(2) | 11646.5(10) | 48.5(7) |
| C4 | 1899(3) | 6221(2) | 11779.8(11) | 57.3(8) |
| C5 | 1775(3) | 5331(2) | 11584.1(12) | 58.5(8) |
| C6 | 2593(2) | 4922(2) | 11262.2(10) | 46.9(7) |
| C7 | 2397(3) | 3956(2) | 11047.7(13) | 59.4(9) |
| C8 | 1563(3) | 4189(3) | 10654.7(14) | 73.8(11) |
| C9 | 2023(4) | 3104(2) | 11441.7(16) | 91.1(14) |
| C10 | 4730(2) | 6945.3(17) | 11195.8(9) | 37.8(5) |
| C11 | 5428(3) | 6925(2) | 11638.5(10) | 52.2(7) |
| C12 | 4409(3) | 8049.0(18) | 10982.3(10) | 46.2(6) |
| C13 | 3755(2) | 7510.3(17) | 9643.9(8) | 31.3(5) |
| C14 | 4449(2) | 8337.5(17) | 9576.7(8) | 31.7(5) |
| C15 | 3985(2) | 9302.8(17) | 9666.7(9) | 37.3(5) |
| C16 | 2871(2) | 9479.7(18) | 9822.5(9) | 39.7(6) |
| C17 | 2195(2) | 8681.7(19) | 9884.5(9) | 41.1(6) |
| C18 | 2616(2) | 7696.5(18) | 9800.6(9) | 38.7(6) |
| C19 | 1738(11) | 6950(12) | 9768(6) | 51(3) |
| C20 | 1193(10) | 7118(12) | 9273(6) | 68(3) |
| C21 | 829(12) | 6918(9) | 10191(7) | 66(3) |
| C22 | 5688(2) | 8171.4(17) | 9424.4(9) | 36.2(5) |
| C23 | 6369(2) | 7873.8(19) | 9871.2(10) | 44.5(6) |
| C24 | 6154(2) | 9096.1(19) | 9105.7(10) | 46.6(6) |
| C25 | 4776(2) | 6739.2(17) | 8475.8(8) | 32.9(5) |
| C26 | 4379(2) | 7758.8(19) | 8405.7(9) | 39.3(5) |
| C27 | 4521(3) | 8350(2) | 7960.1(9) | 48.4(7) |
| C28 | 5065(2) | 7940.5(18) | 7563.2(9) | 38.1(5) |
| C29 | 5469(3) | 6938(2) | 7617.7(9) | 49.3(7) |
| C30 | 5326(3) | 6347(2) | 8067.1(10) | 52.9(7) |
| C31 | 3213(2) | 5228.6(17) | 8943.3(8) | 32.9(5) |
| C32 | 2751(2) | 4512.6(17) | 9306.2(8) | 33.6(5) |
| C33 | 1840(2) | 3998.5(19) | 9232.3(9) | 40.1(6) |
| C34 | 1345(2) | 4187(2) | 8789.3(10) | 46.0(6) |
| C35 | 1776(3) | 4884(2) | 8424.8(10) | 51.5(7) |
| C36 | 2699(2) | 5396(2) | 8502.5(9) | 43.8(6) |
| Co2 | 4494.4(3) | 5518.8(2) | 5237.2(2) | 25.68(9) |
| Si2 | 5613.9(5) | 6665.5(4) | 4340.1(2) | 26.90(13) |
| N3 | 4544.5(15) | 6692.3(13) | 4791.6(6) | 27.6(4) |
| N4 | 5778.7(15) | 5426.6(13) | 4210.2(6) | 28.2(4) |
| C37 | 3751.6(19) | 7531.9(16) | 4883.4(8) | 30.4(5) |
| C38 | 3906.5(19) | 8152.4(16) | 5248.0(8) | 32.8(5) |
| C39 | 3126(2) | 8984.4(18) | 5313.0(10) | 40.0(6) |
| C40 | 2198(2) | 9192.0(19) | 5041.0(11) | 46.7(6) |
| C41 | 2029(2) | 8560(2) | 4698.4(10) | 44.3(6) |
| C42 | 2785(2) | 7733.1(18) | 4610.3(9) | 36.5(5) |
| C43 | 2561(2) | 7073(2) | 4226.9(9) | 42.0(6) |
| C44 | 1678(2) | 6335(2) | 4414.7(11) | 47.4(6) |
| C45 | 2231(3) | 7687(3) | 3745.0(11) | 64.1(9) |
| C46 | 4903(2) | 7961.3(18) | 5563.0(8) | 34.5(5) |
| C47 | 5731(2) | 8789.9(19) | 5425.8(9) | 41.1(6) |
| C48 | 4542(2) | 7871(2) | 6109.3(9) | 43.7(6) |
| C49 | 6263.7(19) | 5026.0(17) | 3778.8(8) | 31.7(5) |
| C50 | 7380(2) | 4568.7(19) | 3751.1(9) | 37.2(5) |
| C51 | 7826(2) | 4203(2) | 3319.3(10) | 49.7(7) |
| C52 | 7212(3) | 4270(3) | 2918.1(10) | 56.8(8) |
| C53 | 6109(3) | 4693(2) | 2945.4(9) | 50.7(7) |
| C54 | 5606(2) | 5054.2(18) | 3371.9(8) | 36.7(5) |
| C55 | 4375(2) | 5420(2) | 3407.0(9) | 41.4(6) |
| C56 | 3682(2) | 4568(2) | 3665.1(9) | 44.2(6) |
| C57 | 3900(3) | 5831(2) | 2914.9(10) | 57.9(8) |
| C58 | 8113(2) | 4419(2) | 4181.7(9) | 39.0(5) |
| C59 | 8635(2) | 3318(2) | 4272.9(11) | 49.2(7) |
| C60 | 9036(2) | 5154(2) | 4123.5(12) | 51.5(7) |
| C61 | 6947.8(19) | 7045.4(16) | 4567.8(8) | 30.5(5) |
| C62 | 7529(2) | 7866.0(18) | 4332.1(9) | 36.0(5) |
| C63 | 8488(2) | 8147.2(19) | 4513.4(10) | 40.9(6) |
| C64 | 8895(2) | 7621.7(19) | 4934.2(10) | 41.5(6) |
| C65 | 8350(2) | 6795.8(18) | 5173.6(9) | 37.2(5) |
| C66 | 7393.1(19) | 6520.2(17) | 4990.9(8) | 32.9(5) |
| C67 | 5373.4(19) | 7683.2(16) | 3801.1(8) | 31.0(5) |
| C68 | 4913(2) | 8661.0(17) | 3876.4(9) | 38.7(5) |
| C69 | 4797(2) | 9444.4(19) | 3494.3(10) | 44.1(6) |
| C70 | 5152(3) | 9269(2) | 3025.0(10) | 50.3(7) |
| C71 | 5651(3) | 8313(2) | 2938.3(10) | 62.5(9) |
| C72 | 5756(3) | 7539.1(19) | 3321.4(9) | 48.9(7) |
| O1 | 667(5) | 619(7) | 8471(2) | 65.9(16) |
| O2 | 960(40) | 1680(20) | 7391(9) | 73(3) |
| O3 | 1635(9) | -739(13) | 7515(6) | 61.7(14) |
| O4 | -882(3) | 279(4) | 7648(2) | 51.1(12) |
| C81 | -2588(4) | -391(4) | 7573(3) | 74.2(14) |
| C82 | -2807(4) | 693(4) | 7734(3) | 63.0(13) |
| C83 | -1729(3) | 1117(3) | 7588.9(19) | 61.2(11) |
| C85 | -1401(3) | -670(3) | 7639(2) | 59.1(11) |
| C87 | 1160(20) | 1687(14) | 6855(7) | 80(3) |
| C88 | 960(20) | 2859(13) | 6711(7) | 78(3) |
| C89 | 1660(20) | 3173(15) | 7056(8) | 88(3) |
| C90 | 1870(20) | 2315(15) | 7415(8) | 83(3) |
| C97 | 2237(5) | -421(6) | 7044(3) | 84.1(17) |
| C98 | 1720(8) | -450(7) | 6605(3) | 150(4) |
| C99 | 1523(6) | -1782(5) | 7618(3) | 89.0(16) |
| C100 | 1040(8) | -2073(6) | 8114(4) | 134(3) |
| C102 | -416(7) | 831(7) | 8764(3) | 82.3(19) |
| C103 | -522(12) | -139(11) | 9146(5) | 109(4) |
| C104 | 1602(5) | 783(6) | 8747(2) | 79.1(17) |
| C105 | 2672(7) | 514(8) | 8459(4) | 63(2) |
| Li1 | 654(4) | 396(4) | 7775.1(19) | 54.5(12) |
| O3A | 1470(20) | -770(30) | 7539(16) | 77(3) |
| C97A | 1995(12) | -952(9) | 7078(6) | 79(2) |
| C98A | 2050(11) | -2019(9) | 7124(5) | 96(3) |
| C99A | 1464(13) | -1752(11) | 7941(6) | 83(3) |
| C73 | 1184(12) | -2397(11) | 7569(5) | 105(3) |
| O1A | 1017(7) | 426(10) | 8425(3) | 71(2) |
| C74 | 2079(7) | -25(8) | 8583(3) | 70(2) |
| C1A | 2903(12) | 732(13) | 8559(6) | 84(4) |
| C1B | 267(10) | 841(9) | 8803(4) | 93(2) |
| C1C | -696(16) | 153(16) | 9002(8) | 120(5) |
| O4A | -896(10) | 85(14) | 7727(7) | 59(2) |
| C85A | -1275(9) | -284(11) | 7292(5) | 65(2) |
| C81A | -2458(11) | -192(13) | 7327(6) | 64(2) |
| C82A | -2845(12) | 323(14) | 7775(7) | 67(3) |
| C83A | -1860(9) | 500(11) | 8008(5) | 65(2) |

**Table *S*6.** Anisotropic displacement parameters (Å^2^×10^3^) for **2**[Li(OEt_2_)_4_]. The anisotropic displacement factor exponent takes the form: -2π^2^[h^2^a^*2^U_11_ + 2hka*b*U_12_ +…].

| **Atom** | **U_11_** | **U_22_** | **U_33_** | **U_23_** | **U_13_** | **U_12_** |
| --- | --- | --- | --- | --- | --- | --- |
| Co1 | 41.2(2) | 26.49(19) | 24.32(18) | -5.25(14) | 3.87(15) | -10.09(15) |
| Si1 | 43.0(3) | 26.0(3) | 23.5(3) | -4.6(2) | 3.1(2) | -10.8(3) |
| N1 | 42.6(11) | 29.8(10) | 26.4(9) | -7.5(8) | 3.6(8) | -8.7(8) |
| N2 | 42.5(11) | 25.8(9) | 26.0(9) | -5.0(7) | 2.8(8) | -8.8(8) |
| C1 | 50.3(14) | 30.6(12) | 29.3(12) | -6.2(9) | 7.2(10) | -7.6(10) |
| C2 | 56.5(15) | 28.3(12) | 28.1(12) | -3.5(9) | 1.4(10) | -5.9(11) |
| C3 | 68.7(18) | 36.0(14) | 41.0(15) | -13.2(11) | 11.4(13) | -6.9(13) |
| C4 | 70(2) | 45.9(16) | 54.3(18) | -16.0(14) | 27.7(15) | -6.9(14) |
| C5 | 60.6(18) | 46.3(16) | 67(2) | -16.2(14) | 30.7(15) | -16.5(14) |
| C6 | 54.7(16) | 35.3(13) | 50.6(16) | -11.3(12) | 18.0(13) | -12.9(12) |
| C7 | 57.0(18) | 41.4(15) | 82(2) | -26.5(15) | 31.8(16) | -21.5(13) |
| C8 | 58.5(19) | 76(2) | 99(3) | -56(2) | 19.5(19) | -25.5(17) |
| C9 | 108(3) | 44.4(18) | 121(3) | -26(2) | 54(3) | -38(2) |
| C10 | 57.8(15) | 25.5(11) | 31.1(12) | -5.5(9) | -1.2(11) | -7.0(11) |
| C11 | 74(2) | 41.5(15) | 44.0(15) | -7.2(12) | -15.7(14) | -6.8(14) |
| C12 | 68.4(18) | 29.5(13) | 39.9(14) | -1.1(11) | -1.4(13) | -6.7(12) |
| C13 | 43.3(13) | 29.0(11) | 22.5(10) | -4.3(9) | -1.0(9) | -6.3(10) |
| C14 | 42.0(13) | 28.4(11) | 25.2(11) | -1.2(9) | -5.2(9) | -6.1(10) |
| C15 | 46.7(14) | 25.7(11) | 40.2(13) | -1.0(10) | -6.8(11) | -7.2(10) |
| C16 | 49.5(14) | 30.2(12) | 40.4(14) | -7.6(10) | -6.0(11) | -1.4(11) |
| C17 | 42.5(14) | 40.6(14) | 41.7(14) | -12.6(11) | 2.2(11) | -5.7(11) |
| C18 | 44.2(14) | 33.4(12) | 40.1(13) | -11.3(10) | 6.6(11) | -10.2(11) |
| C19 | 41(5) | 42(5) | 70(7) | -20(5) | 19(5) | -3(4) |
| C20 | 53(5) | 68(7) | 94(9) | -36(6) | 4(6) | -32(5) |
| C21 | 57(6) | 47(4) | 92(8) | -17(5) | 31(5) | -13(4) |
| C22 | 40.8(13) | 26.4(11) | 42.6(13) | -4.2(10) | -2.8(10) | -8.8(10) |
| C23 | 45.1(14) | 30.6(12) | 58.9(17) | -2.6(11) | -12.3(12) | -4.6(11) |
| C24 | 46.7(15) | 36.1(13) | 55.6(17) | 1.5(12) | 1.8(12) | -10.9(11) |
| C25 | 41.1(13) | 30.1(11) | 28.7(11) | -4.1(9) | 1.7(9) | -12.4(10) |
| C26 | 50.2(14) | 38.9(13) | 26.7(12) | -3.7(10) | 0.9(10) | 4.2(11) |
| C27 | 73.6(19) | 35.7(13) | 31.3(13) | 1.8(11) | -1.7(12) | 9.3(13) |
| C28 | 50.3(14) | 36.4(13) | 27.5(12) | 2.8(10) | -0.5(10) | -13.5(11) |
| C29 | 76(2) | 39.3(14) | 29.3(13) | -4.3(11) | 15.0(13) | -4.9(13) |
| C30 | 92(2) | 29.1(13) | 33.3(14) | -2.5(11) | 12.7(14) | -0.9(13) |
| C31 | 44.1(13) | 27.7(11) | 27.8(11) | -4.7(9) | 0.1(9) | -7.8(10) |
| C32 | 41.3(13) | 28.9(11) | 30.9(12) | -2.8(9) | -0.3(10) | -7.6(10) |
| C33 | 46.5(14) | 33.9(13) | 39.9(14) | -1.8(10) | 2.4(11) | -11.2(11) |
| C34 | 51.2(15) | 44.6(15) | 45.9(15) | -7.9(12) | -1.7(12) | -21.5(12) |
| C35 | 67.7(19) | 54.2(17) | 36.4(14) | -1.6(12) | -11.5(13) | -23.8(14) |
| C36 | 60.2(16) | 40.2(14) | 32.4(13) | 0.6(11) | -2.1(11) | -19.4(12) |
| Co2 | 32.17(19) | 22.12(17) | 22.78(17) | -1.62(13) | 0.14(13) | -5.84(14) |
| Si2 | 33.6(3) | 23.3(3) | 24.0(3) | -0.7(2) | -1.3(2) | -7.4(2) |
| N3 | 32.8(9) | 22.6(9) | 27.4(9) | -1.8(7) | -1.5(7) | -5.3(7) |
| N4 | 36.2(10) | 25.7(9) | 23.5(9) | -3.2(7) | -0.9(7) | -6.7(8) |
| C37 | 35.2(12) | 24.8(11) | 30.6(11) | 0.3(9) | -0.4(9) | -6.2(9) |
| C38 | 38.2(12) | 24.6(11) | 36.0(12) | -3.7(9) | 1.7(10) | -8.4(9) |
| C39 | 47.6(14) | 26.0(11) | 47.0(15) | -9.1(10) | 2.0(11) | -5.0(10) |
| C40 | 45.6(15) | 30.8(13) | 61.1(18) | -4.3(12) | -0.8(13) | 5.6(11) |
| C41 | 39.2(13) | 39.7(14) | 53.0(16) | -2.4(12) | -7.9(12) | 1.7(11) |
| C42 | 36.9(12) | 33.8(12) | 38.3(13) | -0.6(10) | -4.2(10) | -3.9(10) |
| C43 | 39.7(13) | 49.1(15) | 38.6(14) | -8.5(11) | -8.7(11) | -1.6(11) |
| C44 | 47.5(15) | 44.5(15) | 53.6(17) | -17.5(13) | -4.7(12) | -4.5(12) |
| C45 | 79(2) | 66(2) | 49.6(18) | 0.3(15) | -24.3(16) | -15.1(17) |
| C46 | 43.6(13) | 28.9(11) | 32.8(12) | -7.0(9) | -2.3(10) | -7.2(10) |
| C47 | 44.3(14) | 39.2(13) | 42.5(14) | -10.4(11) | -1.5(11) | -11.9(11) |
| C48 | 56.5(16) | 40.1(14) | 36.1(13) | -7.7(11) | -1.0(11) | -10.7(12) |
| C49 | 39.5(12) | 30.6(11) | 26.0(11) | -3.3(9) | 2.5(9) | -12.0(10) |
| C50 | 40.1(13) | 38.3(13) | 34.6(13) | -8.2(10) | 5.1(10) | -12.5(11) |
| C51 | 44.1(15) | 66.5(18) | 40.2(15) | -17.2(13) | 10.0(12) | -10.1(13) |
| C52 | 58.5(18) | 80(2) | 34.1(14) | -19.5(14) | 13.0(13) | -13.4(16) |
| C53 | 61.0(18) | 65.9(18) | 27.9(13) | -10.9(12) | -1.7(12) | -13.9(15) |
| C54 | 46.8(14) | 38.9(13) | 25.0(11) | -1.8(10) | -1.4(10) | -10.5(11) |
| C55 | 49.7(15) | 45.5(14) | 30.3(12) | -5.6(11) | -8.3(11) | -4.9(12) |
| C56 | 39.4(13) | 58.6(17) | 36.6(13) | -11.9(12) | -2.1(11) | -7.3(12) |
| C57 | 70(2) | 65.2(19) | 40.4(15) | -4.9(14) | -20.7(14) | -2.2(16) |
| C58 | 32.6(12) | 47.3(14) | 38.5(13) | -12.9(11) | 2.7(10) | -5.3(11) |
| C59 | 44.2(15) | 49.6(16) | 52.9(17) | -8.3(13) | 1.3(12) | 0.1(12) |
| C60 | 38.6(14) | 51.1(16) | 69(2) | -24.4(15) | 1.5(13) | -8.1(12) |
| C61 | 35.1(12) | 26.2(11) | 31.4(11) | -7.7(9) | 2.0(9) | -6.2(9) |
| C62 | 39.0(13) | 32.1(12) | 37.6(13) | -3.6(10) | 0.7(10) | -10.2(10) |
| C63 | 39.1(13) | 33.6(13) | 51.3(15) | -7.2(11) | 3.4(11) | -11.9(10) |
| C64 | 32.4(12) | 41.5(14) | 54.3(16) | -17.1(12) | -4.1(11) | -6.5(10) |
| C65 | 37.4(13) | 38.2(13) | 37.6(13) | -9.1(10) | -6.3(10) | -1.7(10) |
| C66 | 34.8(12) | 30.0(11) | 34.9(12) | -7.0(9) | -0.8(9) | -5.1(9) |
| C67 | 39.8(12) | 26.4(11) | 27.6(11) | 0.5(9) | -3.7(9) | -10.8(9) |
| C68 | 53.1(15) | 27.6(12) | 34.5(13) | -0.8(10) | 2.7(11) | -8.2(11) |
| C69 | 57.4(16) | 27.6(12) | 45.8(15) | 2.7(11) | -2.6(12) | -5.9(11) |
| C70 | 77(2) | 33.3(13) | 39.9(15) | 10.3(11) | -13.7(13) | -12.9(13) |
| C71 | 115(3) | 39.5(15) | 31.4(14) | 2.2(12) | 2.3(16) | -12.1(16) |
| C72 | 80(2) | 29.6(13) | 34.3(14) | 0.6(10) | 5.2(13) | -5.4(13) |
| O1 | 58(3) | 89(4) | 51(2) | -1(2) | -17(2) | -7(3) |
| O2 | 96(5) | 56(5) | 67(5) | -1(5) | -4(5) | -9(5) |
| O3 | 39(3) | 54(2) | 93(3) | -15(2) | 0(3) | -5(2) |
| O4 | 42.1(16) | 49(2) | 65(2) | -17.5(19) | -7.5(15) | 0.3(14) |
| C81 | 57(2) | 69(3) | 102(4) | -24(3) | -11(3) | -15(2) |
| C82 | 49(2) | 55(3) | 88(3) | -26(3) | -2(2) | -1(2) |
| C83 | 52(2) | 55(2) | 78(3) | -20(2) | -8(2) | 6.6(18) |
| C85 | 54(2) | 43(2) | 82(3) | -10(2) | -5(2) | -6.4(17) |
| C87 | 109(6) | 61(5) | 67(5) | 3(5) | 1(5) | -7(5) |
| C88 | 104(5) | 57(5) | 70(5) | 3(4) | 5(5) | -13(5) |
| C89 | 101(5) | 67(5) | 89(5) | 9(5) | 6(5) | -6(5) |
| C90 | 94(5) | 64(5) | 86(5) | 9(5) | -5(5) | -8(5) |
| C97 | 58(3) | 100(4) | 96(4) | -40(4) | 9(3) | 11(3) |
| C98 | 174(7) | 172(7) | 76(5) | -2(5) | 13(5) | 93(6) |
| C99 | 64(3) | 64(3) | 141(4) | -26(4) | 3(3) | -2(2) |
| C100 | 115(6) | 77(5) | 192(8) | 39(5) | 7(6) | 3(4) |
| C102 | 80(4) | 103(4) | 65(4) | -24(3) | -15(3) | 8(4) |
| C103 | 116(7) | 125(8) | 77(7) | -13(5) | 15(5) | 18(6) |
| C104 | 70(3) | 106(4) | 63(3) | -14(3) | -23(3) | 2(3) |
| C105 | 67(5) | 59(5) | 68(5) | -8(3) | -30(3) | -5(4) |
| Li1 | 49(3) | 58(3) | 56(3) | -3(2) | -5(2) | -4(2) |
| O3A | 55(5) | 67(4) | 107(4) | -11(4) | 0(5) | 4(5) |
| C97A | 56(4) | 74(4) | 102(4) | -9(4) | 6(4) | 4(4) |
| C98A | 82(5) | 80(5) | 132(6) | -37(5) | -9(5) | -1(5) |
| C99A | 63(5) | 63(4) | 120(5) | -10(5) | -9(5) | 2(4) |
| C73 | 90(5) | 74(5) | 152(6) | -16(6) | -15(5) | 3(5) |
| O1A | 68(4) | 81(4) | 67(3) | -7(3) | -27(3) | -8(4) |
| C74 | 69(4) | 84(4) | 61(4) | -6(3) | -27(3) | -5(4) |
| C1A | 93(7) | 86(8) | 73(7) | 8(6) | -15(6) | -27(6) |
| C1B | 85(5) | 112(5) | 85(4) | -23(4) | -22(4) | -1(4) |
| C1C | 130(9) | 130(10) | 94(10) | -13(8) | 10(8) | 13(9) |

**Table *S*7.** Hydrogen atom coordinates (Å×10^4^) and isotropic displacement parameters (Å^2^×10^3^) for **22**[Li(OEt_2_)_4_].

| **Atom** | ***x*** | ***y*** | ***z*** | **U(eq)** |
| --- | --- | --- | --- | --- |
| H7 | 3129.02 | 3700.98 | 10888.27 | 71 |
| H8A | 843.42 | 4468.08 | 10796.97 | 111 |
| H8B | 1454.55 | 3559.59 | 10519.55 | 111 |
| H8C | 1854 | 4688.3 | 10393.65 | 111 |
| H9A | 2566.9 | 2977.53 | 11693.61 | 137 |
| H9B | 1981.68 | 2478.56 | 11292.25 | 137 |
| H9C | 1281.43 | 3313.03 | 11590.43 | 137 |
| H10 | 5210.13 | 6608.35 | 10939.93 | 45 |
| H11A | 4996.64 | 7297.26 | 11885.31 | 78 |
| H11B | 6125.66 | 7248.16 | 11535.43 | 78 |
| H11C | 5607.9 | 6215.84 | 11777.75 | 78 |
| H12A | 3992.55 | 8051.67 | 10691.16 | 69 |
| H12B | 5091.83 | 8403.68 | 10892.72 | 69 |
| H12C | 3937.46 | 8397.73 | 11226.49 | 69 |
| H15 | 4448.91 | 9855.45 | 9619.2 | 45 |
| H16 | 2573.41 | 10142.71 | 9886.21 | 48 |
| H17 | 1423.46 | 8803.62 | 9986.82 | 49 |
| H19 | 2147.53 | 6255.17 | 9790.31 | 61 |
| H20A | 699.9 | 7752.02 | 9252.52 | 103 |
| H20B | 751.97 | 6543.04 | 9246.85 | 103 |
| H20C | 1782.09 | 7164.56 | 9006.03 | 103 |
| H21A | 1183.54 | 6872.33 | 10501.57 | 99 |
| H21B | 404.34 | 6321.53 | 10190.58 | 99 |
| H21C | 318.94 | 7540.72 | 10151.98 | 99 |
| H22 | 5785 | 7585.5 | 9226.32 | 43 |
| H23A | 6285.59 | 8432.23 | 10074.86 | 67 |
| H23B | 7163.48 | 7740.23 | 9765.31 | 67 |
| H23C | 6096.42 | 7257.51 | 10060.71 | 67 |
| H24A | 5696.91 | 9293.26 | 8824.74 | 70 |
| H24B | 6932.58 | 8916.63 | 8989.87 | 70 |
| H24C | 6129.35 | 9668.1 | 9299.11 | 70 |
| H26 | 3998.08 | 8060.32 | 8672.79 | 47 |
| H27 | 4239.79 | 9047.93 | 7926.44 | 58 |
| H28 | 5158.13 | 8349.1 | 7256.51 | 46 |
| H29 | 5848.97 | 6645.14 | 7347.94 | 59 |
| H30 | 5611.13 | 5650.31 | 8097.73 | 64 |
| H32 | 3075.92 | 4378.22 | 9612.58 | 40 |
| H33 | 1553.13 | 3515.79 | 9484.78 | 48 |
| H34 | 714.36 | 3838.72 | 8737.52 | 55 |
| H35 | 1444.07 | 5015.18 | 8119.89 | 62 |
| H36 | 2984.77 | 5874.03 | 8247.37 | 53 |
| H39 | 3238.41 | 9416.83 | 5550.17 | 48 |
| H40 | 1679.68 | 9763.65 | 5088.75 | 56 |
| H41 | 1378.07 | 8694.42 | 4517.91 | 53 |
| H43 | 3276.97 | 6651.87 | 4154.39 | 50 |
| H44A | 1916.47 | 5916.34 | 4713.07 | 71 |
| H44B | 1589.02 | 5891.62 | 4165.17 | 71 |
| H44C | 959.05 | 6721.09 | 4486.65 | 71 |
| H45A | 1509.3 | 8082.51 | 3798.51 | 96 |
| H45B | 2164.16 | 7219.9 | 3503.62 | 96 |
| H45C | 2809.12 | 8152.31 | 3624.22 | 96 |
| H46 | 5308.28 | 7294.89 | 5495.7 | 41 |
| H47A | 5966.93 | 8822.32 | 5077.07 | 62 |
| H47B | 6389.93 | 8622.75 | 5618.3 | 62 |
| H47C | 5362.17 | 9452.31 | 5494.84 | 62 |
| H48A | 4138.41 | 8513.22 | 6187.11 | 66 |
| H48B | 5208.38 | 7725.13 | 6298.48 | 66 |
| H48C | 4047.98 | 7315.1 | 6192.13 | 66 |
| H51 | 8576.69 | 3900.1 | 3303.43 | 60 |
| H52 | 7536.84 | 4029.21 | 2625.16 | 68 |
| H53 | 5682.59 | 4739.91 | 2666.97 | 61 |
| H55 | 4283.66 | 5990.82 | 3614.46 | 50 |
| H56A | 3783.92 | 3981.4 | 3478.74 | 66 |
| H56B | 2885.27 | 4816.25 | 3687.11 | 66 |
| H56C | 3932.76 | 4359.61 | 3994.66 | 66 |
| H57A | 4358.98 | 6361.75 | 2746.48 | 87 |
| H57B | 3124.76 | 6120.45 | 2969.86 | 87 |
| H57C | 3913.93 | 5272.85 | 2713.54 | 87 |
| H58 | 7619.49 | 4558.76 | 4478.8 | 47 |
| H59A | 8041.88 | 2847.46 | 4302.34 | 74 |
| H59B | 9025.12 | 3225.98 | 4575.81 | 74 |
| H59C | 9172.3 | 3175.82 | 3998.95 | 74 |
| H60A | 9563.28 | 5004.13 | 3847.51 | 77 |
| H60B | 9437.19 | 5071.52 | 4423.14 | 77 |
| H60C | 8699.35 | 5856.67 | 4062.57 | 77 |
| H62 | 7258.83 | 8237.39 | 4041.49 | 43 |
| H63 | 8864.04 | 8704.35 | 4346.45 | 49 |
| H64 | 9544.58 | 7821.43 | 5060.5 | 50 |
| H65 | 8633.68 | 6422.72 | 5461.15 | 45 |
| H66 | 7027.8 | 5956.85 | 5158.24 | 39 |
| H68 | 4672.08 | 8794.91 | 4199.27 | 46 |
| H69 | 4472.28 | 10100.13 | 3557 | 53 |
| H70 | 5057.39 | 9798.24 | 2761.36 | 60 |
| H71 | 5918 | 8193.33 | 2616.36 | 75 |
| H72 | 6098.96 | 6890.06 | 3257.37 | 59 |
| H81A | -3060.35 | -877 | 7778.51 | 89 |
| H81B | -2749.57 | -381.36 | 7226.65 | 89 |
| H82A | -3425.98 | 1095.2 | 7561.17 | 76 |
| H82B | -2984.8 | 661.64 | 8091.11 | 76 |
| H83A | -1713.2 | 1434.27 | 7244.36 | 73 |
| H83B | -1603.54 | 1642.19 | 7798.12 | 73 |
| H85A | -1052.21 | -1033.86 | 7367.45 | 71 |
| H85B | -1306.79 | -1120.93 | 7950.4 | 71 |
| H87A | 610.34 | 1307.55 | 6718.07 | 96 |
| H87B | 1934 | 1419.38 | 6758.49 | 96 |
| H88A | 1234.47 | 3070.85 | 6368.69 | 94 |
| H88B | 164.97 | 3110.8 | 6764.98 | 94 |
| H89A | 2371.31 | 3392.53 | 6889.27 | 106 |
| H89B | 1264.62 | 3751.89 | 7211.7 | 106 |
| H90A | 1863.36 | 2533.32 | 7743.8 | 99 |
| H90B | 2604.13 | 1945.37 | 7338.07 | 99 |
| H97A | 2427.36 | 288.65 | 7048.17 | 101 |
| H97B | 2955.88 | -852.36 | 7027.39 | 101 |
| H98A | 2185.97 | -137.04 | 6328.8 | 225 |
| H98B | 977.34 | -72.94 | 6619.99 | 225 |
| H98C | 1637.76 | -1161.02 | 6561.7 | 225 |
| H99A | 2272.1 | -2156.56 | 7575.26 | 107 |
| H99B | 1038.36 | -1989.13 | 7380.45 | 107 |
| H10A | 331.65 | -1658.08 | 8169.08 | 202 |
| H10B | 1567.27 | -1958.24 | 8351.19 | 202 |
| H10C | 898.93 | -2796.11 | 8154.58 | 202 |
| H10D | -1055.98 | 932.79 | 8551.91 | 99 |
| H10E | -395.42 | 1447.12 | 8928.95 | 99 |
| H10F | -487.15 | -745.75 | 8975.22 | 163 |
| H10G | -1240.57 | -74 | 9339.6 | 163 |
| H10H | 96.31 | -205.24 | 9362.68 | 163 |
| H10I | 1569.19 | 350.53 | 9066.47 | 95 |
| H10J | 1561.89 | 1506.1 | 8806.64 | 95 |
| H10K | 2549.55 | 634.51 | 8109.11 | 95 |
| H10L | 2916.11 | -206.97 | 8548.99 | 95 |
| H10M | 3251.8 | 938.92 | 8529.29 | 95 |
| H97C | 1532.27 | -638.51 | 6807.66 | 94 |
| H97D | 2752.17 | -698.6 | 7025.55 | 94 |
| H98D | 2821.9 | -2309.46 | 7189.23 | 116 |
| H98E | 1846.83 | -2246.63 | 6817.46 | 116 |
| H99C | 2203.3 | -1961.28 | 8075.44 | 99 |
| H99D | 868.17 | -1690.42 | 8207.98 | 99 |
| H73A | 393.83 | -2251.1 | 7482.93 | 126 |
| H73B | 1329.91 | -3134.39 | 7680.17 | 126 |
| H74A | 2367.35 | -557.74 | 8374.43 | 85 |
| H74B | 1976.27 | -357.31 | 8923.45 | 85 |
| H1AA | 3054.42 | 1021.6 | 8218.3 | 126 |
| H1AB | 3602.09 | 403.01 | 8689.4 | 126 |
| H1AC | 2606.02 | 1277.18 | 8753.23 | 126 |
| H1BA | -68.89 | 1520.32 | 8668.93 | 111 |
| H1BB | 711.3 | 938.35 | 9077.65 | 111 |
| H1CA | -959.82 | -154.57 | 8732.37 | 180 |
| H1CB | -1320.25 | 567.74 | 9149.13 | 180 |
| H1CC | -414.81 | -388.01 | 9249.06 | 180 |
| H85C | -986.68 | 126.96 | 6990.99 | 78 |
| H85D | -990.08 | -1006.14 | 7278.47 | 78 |
| H81C | -2746.83 | -874.53 | 7356.2 | 76 |
| H81D | -2743.54 | 220.8 | 7030.98 | 76 |
| H82C | -3285.29 | 978.78 | 7680.29 | 81 |
| H82D | -3326.91 | -118.07 | 8002.09 | 81 |

**Table *S*8.** Fractional atomic coordinates (×10^4^) and equivalent isotropic displacement parameters (Å^2^×10^3^) for **3**[K-(18-C-6)]_2_. U_eq_ is defined as one third of the trace of the orthogonalised U_IJ_ tensor.

| **Atom** | ***x*** | ***y*** | ***z*** | **U(eq)** |
| --- | --- | --- | --- | --- |
| C1 | 6790.6(9) | 8528.7(16) | 3681.0(14) | 30.8(6) |
| C2 | 6844.1(11) | 9250.8(16) | 3660.6(16) | 38.2(7) |
| C3 | 6683.2(12) | 9608.0(17) | 3029.0(17) | 44.2(8) |
| C4 | 6475.1(12) | 9279.9(19) | 2415.8(17) | 45.9(8) |
| C5 | 6434.3(10) | 8569.5(18) | 2419.6(16) | 41.4(7) |
| C6 | 6591.9(9) | 8185.6(16) | 3037.6(15) | 32.8(6) |
| C7 | 7093.3(14) | 9635.9(18) | 4313.5(18) | 52.9(10) |
| C8 | 7549.1(15) | 9746(2) | 4300(2) | 67.6(12) |
| C9 | 6897.1(17) | 10321(2) | 4423(3) | 76.3(14) |
| C10 | 6545.7(10) | 7409.3(17) | 3000.7(16) | 37.2(7) |
| C11 | 6685.4(11) | 7097.7(18) | 2389.5(18) | 43.2(8) |
| C12 | 6090.6(12) | 7194(2) | 2957.9(19) | 50.3(9) |
| C13 | 7157.7(9) | 8099.7(16) | 6473.2(14) | 29.8(6) |
| C14 | 7283.1(10) | 8769.2(16) | 6740.3(15) | 35.4(7) |
| C15 | 7328.6(11) | 8917.6(18) | 7446.6(16) | 41.1(7) |
| C16 | 7258.6(10) | 8427.7(19) | 7905.6(16) | 42.6(8) |
| C17 | 7140.4(10) | 7769.4(19) | 7652.1(16) | 39.5(7) |
| C18 | 7090.2(9) | 7590.2(16) | 6953.2(15) | 33.2(7) |
| C19 | 7388.7(13) | 9318.7(17) | 6271.6(17) | 46.4(8) |
| C20 | 7866.1(14) | 9389(2) | 6399(2) | 64.1(12) |
| C21 | 7190.6(13) | 10015(2) | 6344(3) | 64.6(11) |
| C22 | 6946.3(10) | 6868.6(17) | 6704.6(16) | 38.1(7) |
| C23 | 7213.9(12) | 6310.7(19) | 7173.4(18) | 48.4(8) |
| C24 | 6469.1(11) | 6781(2) | 6640.5(19) | 51.7(9) |
| C25 | 6302.1(9) | 8701.0(15) | 5031.4(16) | 32.7(6) |
| C26 | 6221.2(10) | 8893.5(19) | 5666.8(18) | 43.5(8) |
| C27 | 5894.1(11) | 9359(2) | 5681(2) | 55.8(10) |
| C28 | 5639.2(11) | 9632.6(19) | 5056(2) | 53.8(10) |
| C29 | 5705.3(11) | 9445.2(19) | 4424(2) | 52.2(9) |
| C30 | 6032.5(11) | 8986.8(19) | 4413.3(19) | 45.8(8) |
| C31 | 6382.9(9) | 7195.4(16) | 4844.0(14) | 31.6(6) |
| C32 | 5952.0(10) | 7180.8(18) | 4828.3(16) | 38.6(7) |
| C33 | 5723.9(11) | 6562.4(19) | 4786.7(19) | 46.5(8) |
| C34 | 5923.1(11) | 5945.3(18) | 4746.2(18) | 45.2(8) |
| C35 | 6348.2(11) | 5940.9(18) | 4734.2(17) | 40.3(7) |
| C36 | 6573.6(10) | 6557.9(16) | 4789.5(16) | 36.2(7) |
| Co1 | 7426.1(2) | 7595.1(2) | 4395.5(2) | 27.51(13) |
| N1 | 6942.0(7) | 8162.5(12) | 4317.8(12) | 28.7(5) |
| N2 | 7118.3(7) | 7938.8(12) | 5757.8(12) | 28.6(5) |
| Si1 | 6719.3(2) | 8021.6(4) | 4993.9(4) | 27.14(18) |
| C37 | 5007.5(11) | 2057(2) | 150(2) | 50.8(9) |
| C38 | 5195.3(12) | 2412(2) | 842(2) | 53.5(9) |
| C39 | 5138.3(12) | 3426(3) | 1475(2) | 65.3(12) |
| C40 | 4862.1(13) | 4035(3) | 1469(2) | 65.2(11) |
| C41 | 4187.2(13) | 4379(2) | 1589(2) | 55.3(9) |
| C42 | 3752.2(13) | 4127(2) | 1575.0(19) | 54.7(9) |
| C43 | 3123.1(11) | 3634(2) | 827(2) | 49.8(9) |
| C44 | 2935.8(11) | 3368.3(19) | 95.7(19) | 47.7(8) |
| C45 | 3019.7(11) | 2484.1(19) | -682.1(18) | 44.9(8) |
| C46 | 3242.8(11) | 1822.1(19) | -705.7(19) | 45.9(8) |
| C47 | 3930.2(12) | 1330.2(18) | -523.1(18) | 45.0(8) |
| C48 | 4377.3(12) | 1511.1(19) | -525.8(18) | 46.7(8) |
| C49 | 4383(2) | 1359(4) | 1725(3) | 124(3) |
| C50 | 4250.4(19) | 1024(3) | 2271(3) | 98.9(19) |
| C51 | 3937.0(15) | 1498(3) | 2448(2) | 69.7(12) |
| C52 | 3811(2) | 1969(4) | 1807(4) | 124(3) |
| C53 | 3695.2(14) | 4280(2) | -855(3) | 69.3(12) |
| C54 | 3814.5(17) | 4707(3) | -1389(3) | 84.2(14) |
| C55 | 4237(2) | 4519(5) | -1362(4) | 142(3) |
| C56 | 4384(2) | 4007(5) | -826(3) | 138(3) |
| K1 | 4070.6(2) | 2891.3(4) | 463.1(4) | 44.43(19) |
| O1 | 3689.4(7) | 1953.0(12) | -589.7(11) | 41.4(5) |
| O2 | 4577.2(7) | 1865.6(13) | 104.2(12) | 44.3(5) |
| O3 | 4964.8(7) | 3031.4(14) | 850.8(12) | 51.7(6) |
| O4 | 4458.8(8) | 3815.4(13) | 1545.1(13) | 52.6(6) |
| O5 | 3549.0(8) | 3857.1(13) | 899.9(12) | 46.0(6) |
| O6 | 3157.1(7) | 2755.0(13) | 18.0(12) | 43.2(5) |
| O7 | 4122.3(14) | 1923(2) | 1465(2) | 112.7(15) |
| O8 | 4074.8(11) | 3960.1(19) | -446.2(18) | 85.2(11) |

**Table *S*9.** Anisotropic displacement parameters (Å^2^×10^3^) for **3**[K-(18-C-6)]_2_. The anisotropic displacement factor exponent takes the form: -2π^2^[h^2^a^*2^U_11_ + 2hka*b*U_12_ +…].

| **Atom** | **U_11_** | **U_22_** | **U_33_** | **U_23_** | **U_13_** | **U_12_** |
| --- | --- | --- | --- | --- | --- | --- |
| C1 | 31.1(14) | 34.7(16) | 29.0(14) | 2.1(12) | 12.6(11) | 3.1(12) |
| C2 | 51.7(19) | 32.6(17) | 33.7(15) | 0.3(13) | 17.7(14) | 2.0(14) |
| C3 | 65(2) | 28.5(16) | 43.9(18) | 7.3(14) | 22.5(16) | 5.5(15) |
| C4 | 54(2) | 47(2) | 35.1(17) | 11.1(15) | 10.8(15) | 10.9(16) |
| C5 | 44.1(18) | 43.6(19) | 33.8(16) | 4.5(14) | 6.6(13) | 3.7(15) |
| C6 | 32.2(14) | 35.2(16) | 31.7(14) | 2.1(13) | 10.0(12) | 0.7(12) |
| C7 | 93(3) | 32.7(18) | 35.0(17) | -1.8(14) | 21.6(18) | -10.7(19) |
| C8 | 81(3) | 46(2) | 55(2) | -15.0(19) | -15(2) | 12(2) |
| C9 | 100(4) | 55(3) | 91(3) | -37(2) | 55(3) | -19(2) |
| C10 | 40.0(17) | 37.1(17) | 31.5(15) | 1.2(13) | 4.7(13) | -3.7(14) |
| C11 | 45.0(18) | 40.1(19) | 42.9(17) | -3.0(15) | 9.4(14) | 1.3(15) |
| C12 | 53(2) | 54(2) | 44.7(19) | -1.1(17) | 15.7(16) | -11.8(18) |
| C13 | 25.7(13) | 35.9(16) | 29.3(14) | -0.1(12) | 10.2(11) | 2.6(12) |
| C14 | 38.3(16) | 35.1(17) | 32.1(15) | -2.2(13) | 8.5(12) | 4.2(13) |
| C15 | 47.6(18) | 41.3(18) | 32.6(16) | -9.0(14) | 8.3(14) | 7.3(15) |
| C16 | 44.6(18) | 56(2) | 30.4(15) | -5.3(15) | 15.2(13) | 7.3(16) |
| C17 | 37.8(16) | 53(2) | 29.4(15) | 1.7(14) | 12.2(13) | 2.6(15) |
| C18 | 31.9(15) | 40.4(18) | 29.0(15) | 1.1(13) | 11.5(12) | 0.0(13) |
| C19 | 75(2) | 27.0(17) | 33.4(16) | -1.0(13) | 8.0(16) | -2.5(16) |
| C20 | 83(3) | 46(2) | 83(3) | 21(2) | 55(2) | 15(2) |
| C21 | 53(2) | 36(2) | 100(3) | 9(2) | 14(2) | 3.1(17) |
| C22 | 40.7(17) | 44.5(19) | 30.2(15) | 3.4(14) | 11.7(13) | -7.2(14) |
| C23 | 55(2) | 46(2) | 44.1(18) | 6.2(16) | 13.7(16) | -3.5(17) |
| C24 | 44.0(19) | 61(2) | 49(2) | 6.6(18) | 11.2(16) | -15.4(17) |
| C25 | 32.5(15) | 28.1(15) | 40.0(16) | -2.0(13) | 14.3(12) | -0.8(12) |
| C26 | 32.7(16) | 52(2) | 46.7(18) | -11.8(16) | 12.7(14) | 3.1(15) |
| C27 | 33.2(17) | 63(2) | 74(3) | -32(2) | 18.6(17) | -4.1(17) |
| C28 | 29.9(16) | 36.8(19) | 93(3) | -12.6(19) | 14.9(18) | 2.8(14) |
| C29 | 37.7(18) | 45(2) | 75(3) | 17.5(19) | 17.6(17) | 10.5(16) |
| C30 | 40.7(18) | 49(2) | 51.4(19) | 12.4(16) | 19.6(15) | 6.5(15) |
| C31 | 35.4(15) | 34.1(16) | 26.5(13) | -1.6(12) | 10.7(12) | -1.6(13) |
| C32 | 38.5(17) | 38.4(18) | 41.7(17) | -0.6(14) | 15.7(14) | -3.0(14) |
| C33 | 36.4(17) | 51(2) | 55(2) | -1.3(17) | 17.2(15) | -7.9(16) |
| C34 | 51(2) | 37.9(19) | 46.5(19) | -4.6(15) | 14.1(16) | -16.0(16) |
| C35 | 44.8(18) | 34.5(17) | 41.4(17) | -2.4(14) | 11.9(14) | -3.3(14) |
| C36 | 38.9(16) | 34.9(17) | 36.2(15) | 0.8(13) | 12.7(13) | -2.8(13) |
| Co1 | 28.6(2) | 29.9(3) | 26.2(2) | 1.36(19) | 11.22(18) | 2.95(19) |
| N1 | 30.8(12) | 29.2(13) | 27.2(11) | 3.6(10) | 9.9(9) | 4.4(10) |
| N2 | 31.1(12) | 30.4(13) | 26.9(11) | -0.6(10) | 12.4(9) | 2.0(10) |
| Si1 | 28.7(4) | 27.1(4) | 27.3(4) | 0.4(3) | 10.7(3) | 1.0(3) |
| C37 | 40.8(18) | 58(2) | 57(2) | 3.4(18) | 19.5(16) | 7.8(17) |
| C38 | 38.2(18) | 71(3) | 52(2) | 6.6(19) | 13.2(16) | 5.1(18) |
| C39 | 41(2) | 90(3) | 61(2) | -19(2) | 8.6(18) | -10(2) |
| C40 | 55(2) | 75(3) | 67(3) | -24(2) | 19(2) | -18(2) |
| C41 | 68(2) | 47(2) | 51(2) | -5.1(17) | 16.9(18) | 1.4(19) |
| C42 | 69(2) | 54(2) | 46(2) | -0.5(17) | 22.7(18) | 13.2(19) |
| C43 | 50(2) | 48(2) | 60(2) | 6.1(18) | 30.1(17) | 9.7(17) |
| C44 | 40.5(18) | 51(2) | 56(2) | 13.1(17) | 21.3(16) | 8.7(16) |
| C45 | 37.9(17) | 54(2) | 42.2(18) | 5.2(15) | 9.8(14) | -8.6(15) |
| C46 | 44.7(18) | 48(2) | 45.7(18) | 4.6(16) | 13.4(15) | -10.3(16) |
| C47 | 59(2) | 35.5(18) | 40.0(17) | 1.1(14) | 13.3(15) | -1.0(16) |
| C48 | 57(2) | 45(2) | 40.2(17) | -1.2(16) | 17.0(15) | 6.7(17) |
| C49 | 124(5) | 164(7) | 103(4) | 59(5) | 62(4) | 85(5) |
| C50 | 85(4) | 109(5) | 117(5) | 36(4) | 53(3) | 33(3) |
| C51 | 64(3) | 81(3) | 68(3) | 9(2) | 25(2) | 5(2) |
| C52 | 139(6) | 119(5) | 154(6) | 62(5) | 105(5) | 69(5) |
| C53 | 64(3) | 63(3) | 91(3) | -22(2) | 39(2) | -9(2) |
| C54 | 85(3) | 65(3) | 98(4) | 9(3) | 17(3) | 5(3) |
| C55 | 99(4) | 203(8) | 154(6) | 106(6) | 83(5) | 56(5) |
| C56 | 108(5) | 230(9) | 106(4) | 77(5) | 80(4) | 87(5) |
| K1 | 39.4(4) | 47.9(4) | 47.1(4) | -5.6(3) | 13.9(3) | 0.3(3) |
| O1 | 42.8(12) | 39.0(13) | 41.2(12) | 1.2(10) | 9.5(10) | -2.0(10) |
| O2 | 41.8(12) | 52.3(15) | 40.9(12) | -0.4(11) | 15.0(10) | 3.2(11) |
| O3 | 38.9(12) | 67.3(18) | 48.5(13) | -9.3(12) | 11.4(10) | -1.7(12) |
| O4 | 54.5(14) | 52.5(15) | 53.1(14) | -8.7(12) | 18.8(12) | -4.6(12) |
| O5 | 51.6(14) | 48.4(14) | 43.9(13) | 1.0(11) | 23.1(11) | 1.9(11) |
| O6 | 39.6(12) | 47.4(14) | 44.4(12) | 7.6(11) | 14.6(10) | 4.1(10) |
| O7 | 130(3) | 103(3) | 141(3) | 50(3) | 97(3) | 35(3) |
| O8 | 87(2) | 96(3) | 94(2) | 31(2) | 61(2) | 32(2) |

**Table *S*10.** Hydrogen atom coordinates (Å×10^4^) and isotropic displacement parameters (Å^2^×10^3^) for **3**[K-(18-C-6)]_2_.

| **Atom** | ***x*** | ***y*** | ***z*** | **U(eq)** |
| --- | --- | --- | --- | --- |
| H3 | 6719.11 | 10093.87 | 3024.41 | 53 |
| H4 | 6360.51 | 9535.1 | 1994.39 | 55 |
| H5 | 6296.15 | 8337.67 | 1992.73 | 50 |
| H7 | 7099.82 | 9339.3 | 4731.05 | 63 |
| H8A | 7678.72 | 9301.53 | 4241.66 | 101 |
| H8B | 7712.06 | 9959.21 | 4743.89 | 101 |
| H8C | 7554.01 | 10049.56 | 3904.89 | 101 |
| H9A | 6893.65 | 10628.73 | 4027.6 | 114 |
| H9B | 7067.84 | 10529.88 | 4864.17 | 114 |
| H9C | 6602.7 | 10247.38 | 4448.38 | 114 |
| H10 | 6736.93 | 7216.12 | 3448.17 | 45 |
| H11A | 6477.2 | 7220.79 | 1942.21 | 65 |
| H11B | 6699.87 | 6594.81 | 2438.33 | 65 |
| H11C | 6968.84 | 7277.16 | 2395.23 | 65 |
| H12A | 6004.53 | 7396.16 | 3353.03 | 75 |
| H12B | 6075.59 | 6689.91 | 2981.4 | 75 |
| H12C | 5896.95 | 7355.09 | 2511.6 | 75 |
| H15 | 7410.29 | 9369.71 | 7616.7 | 49 |
| H16 | 7290.66 | 8537.86 | 8386.73 | 51 |
| H17 | 7091.98 | 7429.25 | 7968.01 | 47 |
| H19 | 7267.22 | 9164.35 | 5772.14 | 56 |
| H20A | 7995.6 | 9544.95 | 6884.25 | 96 |
| H20B | 7925.75 | 9726.11 | 6068.55 | 96 |
| H20C | 7988.01 | 8941.5 | 6327.66 | 96 |
| H21A | 6877.36 | 9970.6 | 6216.87 | 97 |
| H21B | 7270.4 | 10347.9 | 6028.53 | 97 |
| H21C | 7296.11 | 10175.4 | 6832.64 | 97 |
| H22 | 6984.56 | 6812.84 | 6221.67 | 46 |
| H23A | 7518.6 | 6386.7 | 7213.96 | 73 |
| H23B | 7127.48 | 5856.8 | 6963.89 | 73 |
| H23C | 7167.35 | 6330.86 | 7643.13 | 73 |
| H24A | 6422.04 | 6819.07 | 7108.79 | 78 |
| H24B | 6373.34 | 6327.5 | 6439.52 | 78 |
| H24C | 6305.73 | 7141.6 | 6331.87 | 78 |
| H26 | 6392.06 | 8704 | 6097.52 | 52 |
| H27 | 5847.01 | 9487.35 | 6117.85 | 67 |
| H28 | 5418.12 | 9950.7 | 5065.79 | 65 |
| H29 | 5527.89 | 9628.27 | 3995.46 | 63 |
| H30 | 6075.13 | 8862.66 | 3971.72 | 55 |
| H32 | 5807.71 | 7603.71 | 4846.34 | 46 |
| H33 | 5430.93 | 6569.69 | 4786.44 | 56 |
| H34 | 5770.48 | 5524.38 | 4726.56 | 54 |
| H35 | 6485.39 | 5517.64 | 4688.44 | 48 |
| H36 | 6866.56 | 6546.39 | 4790.22 | 43 |
| H37A | 5012.9 | 2369.92 | -243.99 | 61 |
| H37B | 5178.39 | 1641.69 | 116.37 | 61 |
| H38A | 5174.26 | 2109.31 | 1234.9 | 64 |
| H38B | 5502.09 | 2516.28 | 900.62 | 64 |
| H39A | 5433.22 | 3576.6 | 1496.14 | 78 |
| H39B | 5151.79 | 3139.86 | 1896.62 | 78 |
| H40A | 5002.4 | 4349 | 1861.07 | 78 |
| H40B | 4819.17 | 4289.12 | 1019.54 | 78 |
| H41A | 4166.26 | 4696.14 | 1188.13 | 66 |
| H41B | 4311.59 | 4636.81 | 2031.97 | 66 |
| H42A | 3775.31 | 3763.82 | 1936.72 | 66 |
| H42B | 3579.67 | 4510.52 | 1683.49 | 66 |
| H43A | 2948.39 | 4022.42 | 919.01 | 60 |
| H43B | 3122.62 | 3264.51 | 1173.52 | 60 |
| H44A | 2626.2 | 3270.87 | 15.16 | 57 |
| H44B | 2967.72 | 3716.57 | -253.12 | 57 |
| H45A | 3087.72 | 2815.17 | -1017.61 | 54 |
| H45B | 2705.34 | 2409.95 | -820.01 | 54 |
| H46A | 3198.41 | 1507.01 | -337.41 | 55 |
| H46B | 3124.79 | 1599.85 | -1171.19 | 55 |
| H47A | 3799.39 | 1017.23 | -921.18 | 54 |
| H47B | 3932.61 | 1093.71 | -77.28 | 54 |
| H48A | 4540.1 | 1086.67 | -559.46 | 56 |
| H48B | 4371.4 | 1805.97 | -938.08 | 56 |
| H49A | 4684.26 | 1515.6 | 1911.01 | 149 |
| H49B | 4370.67 | 1029.4 | 1336.62 | 149 |
| H50A | 4498.54 | 944.76 | 2688.19 | 119 |
| H50B | 4114.85 | 574.4 | 2105.49 | 119 |
| H51A | 4070.27 | 1761.47 | 2883.12 | 84 |
| H51B | 3685.37 | 1242.66 | 2509.63 | 84 |
| H52A | 3530.55 | 1825 | 1489.44 | 149 |
| H52B | 3785.32 | 2450.23 | 1956.88 | 149 |
| H53A | 3568.63 | 4571.07 | -552.16 | 83 |
| H53B | 3481.59 | 3928.02 | -1087.12 | 83 |
| H54A | 3619.06 | 4616.63 | -1863.53 | 101 |
| H54B | 3799.33 | 5202.53 | -1278.91 | 101 |
| H55A | 4426.22 | 4928.47 | -1254.69 | 171 |
| H55B | 4246.53 | 4333.08 | -1825.03 | 171 |
| H56A | 4415.57 | 3556.43 | -1042.45 | 166 |
| H56B | 4665.96 | 4140.61 | -506.86 | 166 |

3. NMR spectra

**Figure. *S*4** ^1^H NMR spectrum of Li_2_[Ph_2_Si(*N*-2,6-*^i^*Pr_2_C_6_H_3_)_2_] (500 MHz, C_6_D_6_, 293 K).

**Figure. *S*5** ^13^C{^1^H} NMR spectrum of Li_2_[Ph_2_Si(*N*-2,6-*^i^*Pr_2_C_6_H_3_)_2_] (125 MHz, C_6_D_6_, 293 K).

**Figure. *S*6** ^1^H NMR spectrum of **1** (500 MHz, C_6_D_6_, 293 K).

**Figure. *S*7** ^1^H NMR spectrum of **2**[Li(OEt_2_)_4_] (500 MHz, C_6_D_6_, 293 K).

**Figure. *S*8** ^1^H NMR of spectrum **3**[Li]_2_ (500 MHz, C_6_D_6_, 293 K).

4. Elemental analysis report


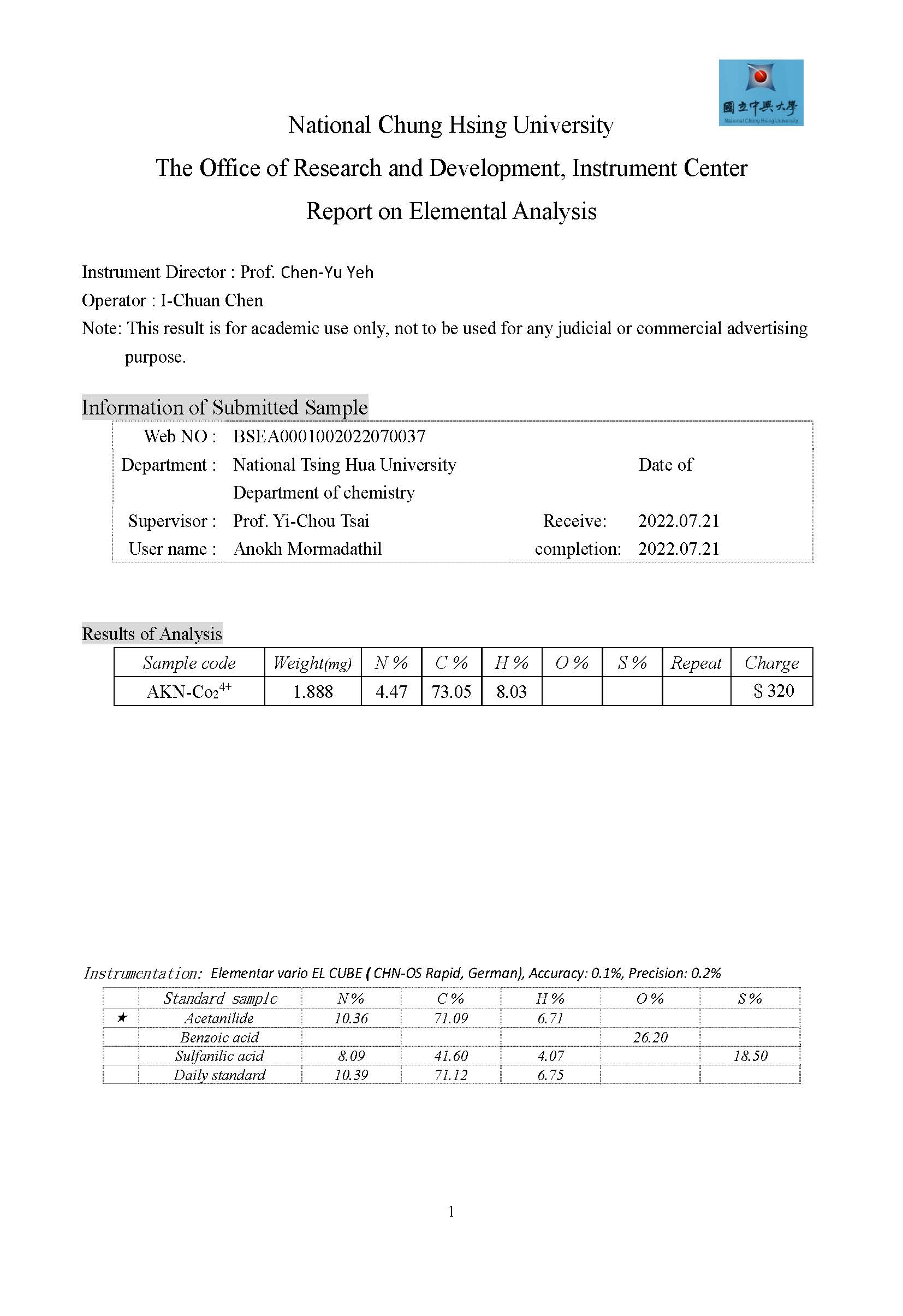


**Figure. *S*9** Elemental analysis report of **1**.


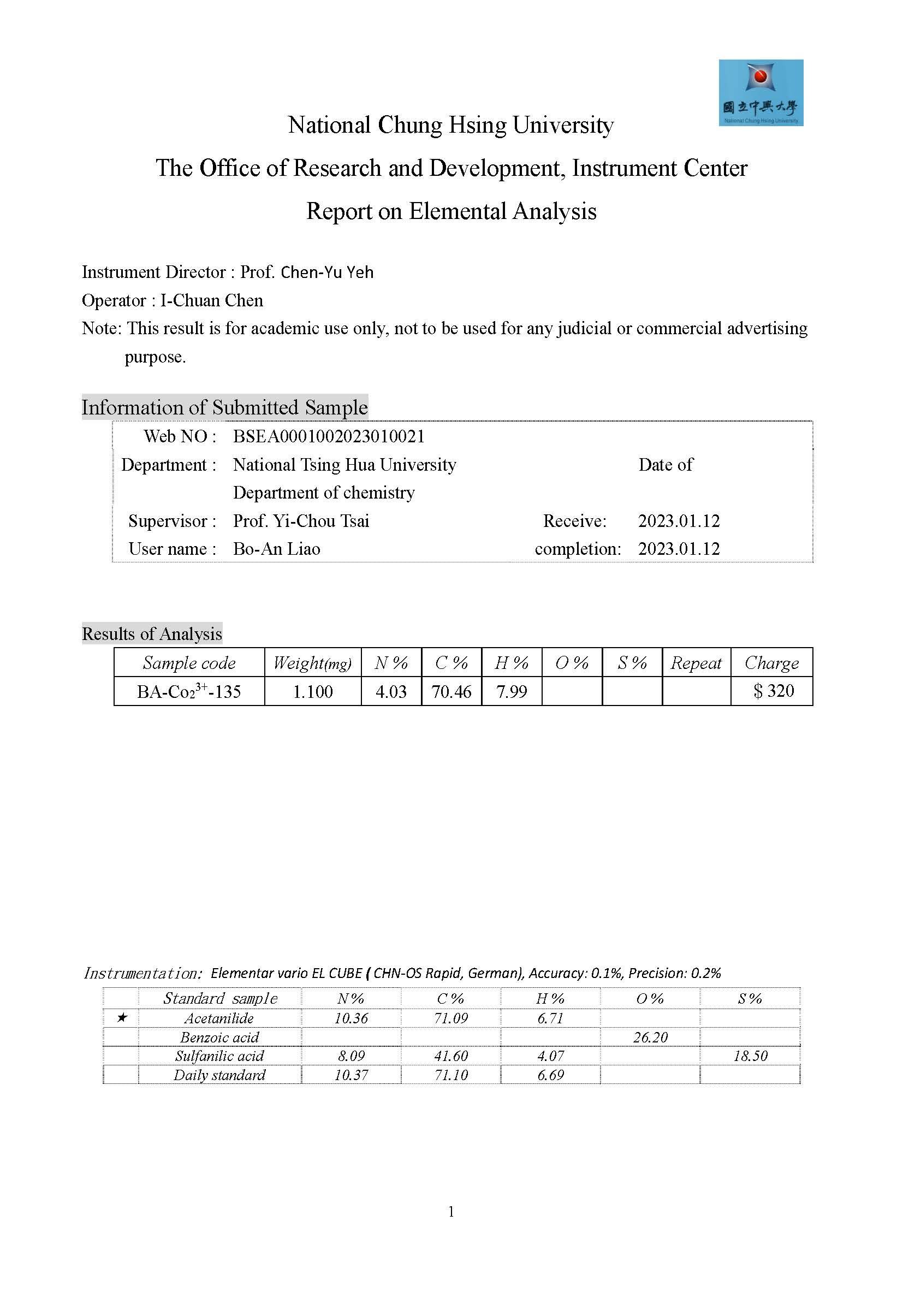


**Figure. *S*10** Elemental analysis report of **2**[Li(OEt_2_)_4_].


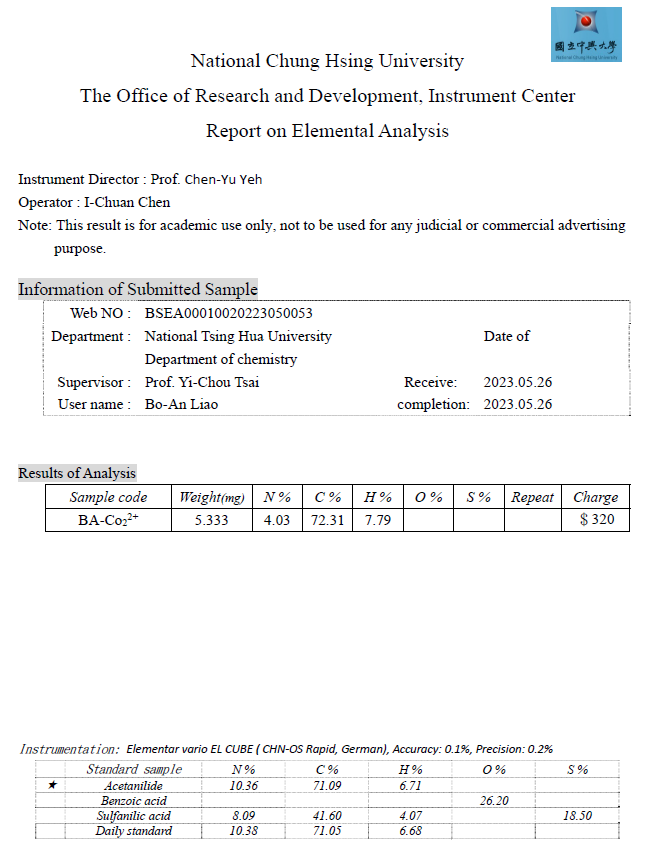


**Figure. *S*11** Elemental analysis report of **3**[Li]_2_.


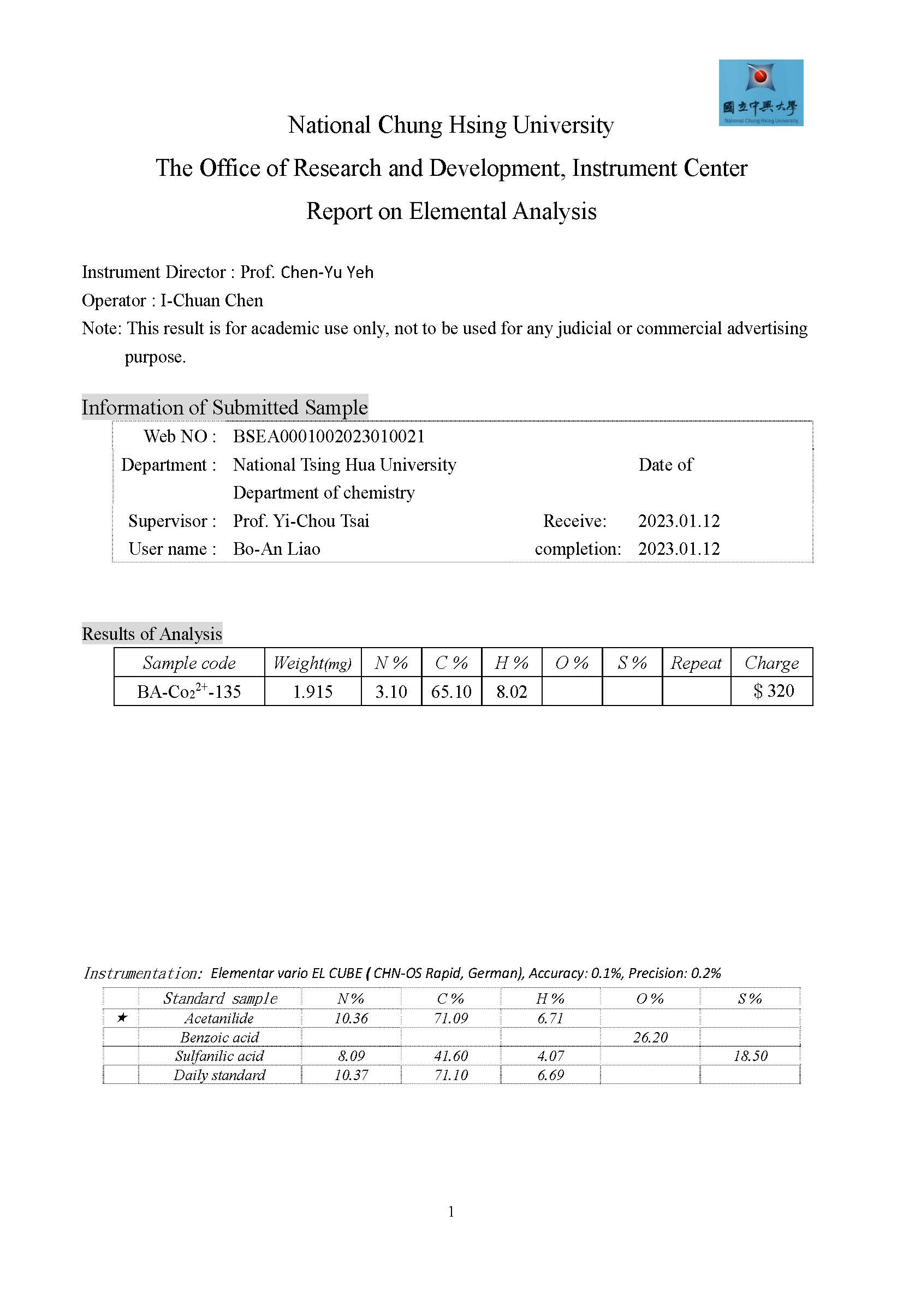


**Figure. *S*12** Elemental analysis report of **3**[K-(18-C-6)]_2_.

5. Evans method





**Figure. *S*13** Evans method NMR spectrum of **1** (500 MHz, 293 K).





**Figure. *S*14** Evans method NMR spectrum of **2** (500 MHz, 293 K).





**Figure. *S*15** Evans method NMR spectrum of **3**[Li]_2_ (500 MHz, 293 K).

6. SQUID





**Figure. *S*16** Temperature dependent *χ*_m_ plot of **1** at 10000 Oe from 2−300 K. (*S* = 2, simulation parameters: *g* = 2.3933, *D*= 6.263 cm^−1^, *E*= 2.088 cm^−1^, z*J* = −0.1345 cm^−1^.)





**Figure. *S*17** Temperature dependent *χ*^−1^ plot of **1** at 10000 Oe from 2−300 K.





**Figure. *S*18** Temperature dependent *χ*_m_ plot of of **2**[Li(OEt_2_)_4_] at 5000 Oe from 2−300 K. (*S* = 5/2, simulation parameters: *g* = 2.4253, *D* = 66.263 cm^−1^, *E* = −1.168 cm^−1,^ z*J* = −0.25 cm^−1^)





**Figure. *S*19** Temperature dependent *χ*^−1^ plot of **2**[Li(OEt_2_)_4_] at 5000 Oe from 2−300 K.





**Figure. *S*20.** Temperature dependent *χ*_m_ plot of **3**[Li]_2_ at 5000 Oe from 2−300 K. (*S* = 2, simulation parameters: *g* = 2.7620, *D* = −77.507 cm^−1^, *E* = −0.122 cm^−1,^ z*J* = −0.570 cm^−1^)





**Figure. *S*21.** Temperature dependent *χ*^−1^ plot of **3**[Li]_2_ at 5000 Oe from 2−300 K.

7. Cyclic Voltammetry





**Figure. *S*22.** Cyclic voltammogram of **1** recorded in THF solution containing 0.10 M [Bu_4_N][PF_6_]. Scan rate is 0.10 V/s





**Figure. *S*23.** Open circuit potential measurement of **1** recorded in THF solution containing 0.10 M [Bu_4_N][PF_6_]. Time interval = 0.10 s

8. Details of computational studies

**Computational methods**

Calculations were performed with the Gaussian 16 software package.^14^ The molecular geometries were optimized without symmetry constraints at the (U)BP86 level of density functional theory (DFT),^15−16^ and stability of wavefunction was checked for optimized structure. Vibrational frequency calculations at the same level of theory have also been performed at 1 atm and 298.15 K to identify all the located stationary points as minima (zero imaginary frequency). The SCF convergence criterion was set to 10^-8^ in all cases. The SDD ECP basis set^16−17^ was used to describe Co atom and a 6-31G(d,p) Pople basis set^17−23^ for H, C, N, and Si atoms (named BS-I). The basis set used for the single-point calculations comprised LanL2DZ ECP basis set^24^ for Co atom and a 6-31G(d,p) Pople basis set^17−23^ for the rest of atoms (named BS-II) with the optimized structures at the (U)BP86/BS-I level. In the literature it is stated that this basis set and the DFT methodology have been shown to give reliable results for Co metal.^25^ Multiwfn 3.8 (dev) program^26^ was used for the analyses of natural bond orbitals (NBO)^27^ and the spin density analysis. The Cartesian coordinates of the optimized geometries are listed in Table *S17*.

**Table *S*11.** DFT optimized structures of the model systems, and relative electronic energies and free energies (in kcal/mol at 1 atm and 298.15 K.) for the various spin states of **1**.

**^2^*^S^*^+1^1^Me/Me^**

|  | *S* = 0 (Singlet) | *S* = 1 (Triplet) | *S* = 2 (Quintet) | *S* = 3 (Septet) | *S* = 4 (Nonet) |
| --- | --- | --- | --- | --- | --- |
| ΔE | 14.25 | 6.72 | 0.00 | 0.43 | 56.36 |
| ΔG | 16.72 | 7.53 | 0.00 | 0.25 | 54.56 |

**Table *S*12.** DFT optimized structures of the model systems, and relative electronic energies and free energies (in kcal/mol at 1 atm and 298.15 K.) for the various spin states of **2**.

**^2^*^S^*^+1^2^Me/Me^**

|  | *S* = 1/2 (Doublet) | *S* = 3/2 (Quartet) | *S* = 5/2 (sextet) | *S* = 7/2 (Octet) | *S* = 9/2 (Decet) |
| --- | --- | --- | --- | --- | --- |
| ΔE | 13.02 | 15.58 | 0.00 | 49.94 | 106.36 |
| ΔG | 13.56 | 16.81 | 0.00 | 50.54 | 106.65 |

**Table *S*13.** DFT optimized structures of the model systems, and relative electronic energies and free energies (in kcal/mol at 1 atm and 298.15 K.) for the various spin states of **3**.

**^2^*^S^*^+1^3^Me/Me^**

|  | *S* = 0 (Singlet) | *S* = 1 (Triplet) | *S* = 2 (Quintet) | *S* = 3 (Septet) | *S* = 4 (Nonet) |
| --- | --- | --- | --- | --- | --- |
| ΔE | 11.78 | 14.36 | 0.00 | 0.69 | 43.31 |
| ΔG | 12.10 | 15.09 | 0.00 | 0.56 | 45.28 |

**

**

**Figure. *S*24.** Comparsions of different substitutions DFT optimized structures for the ground states of **1**−**3**.

**Table *S*14.** Comparisons of the experimental and calculated core structural metrics of **1** (*S* = 2, quintet state). Bond lengths and bond angles are respectively taken in units of (Å) and in units of (°).

**^2^*^S^*^+1^1^R/R'^**

|  | Exp. | Cal. *S* = 2  [R = Dipp, R' = Ph] | Cal. *S* = 2  [R = R' = Me] | Cal. *S* = 0  [R = R' = Me] | Cal. *S* = 1  [R = R' = Me] | Cal. *S* = 3  [R = R' = Me] | Cal. *S* = 4  [R = R' = Me] |
| --- | --- | --- | --- | --- | --- | --- | --- |
| Co(1)−Co(1') | 2.2390(7) | 2.1707 | 2.164 | 2.359 | 2.304 | 2.126 | 2.176 |
| Co(1)−N(1) | 1.878(3) | 1.8375 | 1.817 | 1.775 | 1.824 | 1.833 | 1.882 |
| N(1)−Si(1) | 1.719(3) | 1.7767 | 1.774 | 1.759 | 1.745 | 1.765 | 1.777 |
| Si(1)−N(1') | 1.726(3) | 1.7655 | 1.773 | 1.759 | 1.784 | 1.765 | 1.777 |
| N(1')−Co(1') | 1.862(3) | 1.8375 | 1.817 | 1.775 | 1.767 | 1.833 | 1.882 |
| N(1)−Co(1)−Co(1') | 99.62(8) | 98.63 | 99.28 | 96.13 | 99.03 | 100.16 | 100.08 |
| Si(1)−N(1)−Co(1) | 111.49(13) | 115.58 | 114.48 | 122.76 | 115.80 | 118.07 | 114.22 |
| N(1)−Si(1)−N(2) | 105.70(12) | 101.81 | 101.68 | 102.22 | 102.78 | 103.54 | 105.86 |
| Si(1)−N(2)−Co(1') | 114.63(13) | 114.82 | 114.45 | 122.76 | 121.03 | 118.07 | 114.22 |
| N(2)−Co(1')−Co(1) | 95.95(8) | 99.47 | 99.30 | 96.13 | 95.31 | 100.16 | 100.08 |
| N(1)−Co(1)−Co(1')−N(2) | 0.33(11) | 1.34 | 0.09 | 0.01 | 3.97 | 0.02 | 0.02 |
| Si(1)−N(2)−N(1)−Co(1) | 146.76(16) | 152.17 | 149.96 | 179.98 | 156.51 | 179.67 | 157.92 |

**Table *S*15.** Comparisons of the experimental and calculated core structural metrics of **2** (*S* = 5/2, sextet state). Bond lengths and bond angles are respectively taken in units of (Å) and in units of (°).

**^2^*^S^*^+1^2^R/R'^**

|  | Exp. | Cal. *S* = 5/2  [R = Dipp, R' = Ph] | Cal. *S* = 5/2  [R = R' = CH_3_] | Cal. *S* = 1/2  [R = R' = CH_3_] | Cal. *S* = 3/2  [R = R' = CH_3_] | Cal. *S* = 7/2  [R = R' = CH_3_] | Cal. *S* = 9/2  [R = R' = CH_3_] |
| --- | --- | --- | --- | --- | --- | --- | --- |
| Co(1)−Co(1') | 2.2329(8), 2.2376(8) | 2.20478 | 2.183 | 2.458 | 2.294 | 2.210 | 2.190 |
| Co(1)−N(1) | 1.8748(16), 1.8757(19) | 1.8763 | 1.855 | 1.850 | 1.838 | 1.830 | 1.894 |
| N(1)−Si(1) | 1.718(2), 1.7167(18) | 1.7361 | 1.743 | 1.745 | 1.741 | 1.751 | 1.767 |
| Si(1)−N(1') | 1.7241(19), 1.7174(18) | 1.7567 | 1.743 | 1.735 | 1.741 | 1.751 | 1.767 |
| N(1')−Co(1') | 1.8582(16), 1.8535(19) | 1.8737 | 1.855 | 1.847 | 1.838 | 1.837 | 1.893 |
| N(1)−Co(1)−Co(1') | 99.19(7), 98.98(6) | 98.80 | 99.56 | 94.96 | 97.44 | 101.65 | 99.51 |
| Si(1)−N(1)−Co(1) | 112.12(10). 112.65(9) | 112.37 | 117.00 | 121.29 | 119.68 | 116.70 | 115.67 |
| N(1)−Si(1)−N(2) | 105.42(10), 105.23(9) | 105.20 | 106.90 | 107.08 | 105.38 | 104.58 | 10.65 |
| Si(1)−N(2)−Co(1') | 115.06(10). 115.38(9) | 112.11 | 117.00 | 121.04 | 119.71 | 120.53 | 115.59 |
| N(2)−Co(1')−Co(1) | 96.22(6). 96.28(8) | 99.36 | 99.56 | 95.63 | 97.41 | 96..44 | 99.53 |
| N(1)−Co(1)−Co(1')−N(2) | 0.09(9), 0.22(9) | 0.26 | 0.03 | 0.00 | 0.09 | 2.34 | 0.52 |
| Si(1)−N(2)−N(1)−Co(1) | 148.34(12), 147.67(12) | 147.25 | 179.45 | 179.98 | 174.26 | 179.64 | 161.46 |

**Table *S*16.** Comparisons of the experimental and calculated core structural metrics of **3** (*S* = 2, quintet state). Bond lengths and bond angles are respectively taken in units of (Å) and in units of (°).

**^2^*^S^*^+1^3^R/R'^**

|  | Exp. | Cal. *S* = 2  [R = Dipp, R' = Ph] | Cal. *S* = 2  [R = R' = CH_3_] | Cal. *S* = 0  [R = R' = CH_3_] | Cal. *S* = 1  [R = R' = CH_3_] | Cal. *S* = 3  [R = R' = CH_3_] | Cal. *S* = 4  [R = R' = CH_3_] |
| --- | --- | --- | --- | --- | --- | --- | --- |
| Co(1)−Co(1') | 2.3276(8) | 2.3498 | 2.182 | 2.492 | 2.349 | 2.177 | 2.129 |
| Co(1)−N(1) | 1.888(2) | 1.9073 | 1.857 | 1.855 | 1.867 | 1.856 | 1.833 |
| N(1)−Si(1) | 1.706(2) | 1.7474 | 1.741 | 1.733 | 1.729 | 1.741 | 1.759 |
| Si(1)−N(1') | 1.699(2) | 1.7401 | 1.741 | 1.740 | 1.755 | 1.741 | 1.760 |
| N(1')−Co(1') | 1.896(2) | 1.9056 | 1.856 | 1.854 | 1.817 | 1.856 | 1.833 |
| N(1)−Co(1)−Co(1') | 96.06(7) | 97.17 | 99.63 | 95.38 | 97.20 | 99.70 | 100.153 |
| Si(1)−N(1)−Co(1) | 111.48(12) | 111.31 | 116.79 | 120.40 | 118.24 | 116.79 | 117.83 |
| N(1)−Si(1)−N(2) | 108.97(11) | 108.81 | 107.15 | 108.38 | 107.48 | 107.02 | 104.01 |
| Si(1)−N(2)−Co(1') | 111.20(12) | 111.08 | 116.85 | 121.17 | 119.82 | 116.86 | 117.90 |
| N(2)−Co(1')−Co(1) | 97.41(7) | 97.49 | 99.57 | 94.66 | 97.10 | 99.63 | 100.08 |
| N(1)−Co(1)−Co(1')−N(2) | 0.15(10) | 0.54 | 0.00 | 0.81 | 3.00 | 0.01 | 0.02 |
| Si(1)−N(2)−N(1)−Co(1) | 141.51(15) | 143.93 | 179.12 | 178.87 | 176.62 | 179.16 | 148.47 |





**Figure. *S*25.** The DFT calculated spin density of **1** (isovalue = 0.005 a.u.), **2** (isovalue = 0.007 a.u.), **3** (isovalue = 0.007 a.u.)


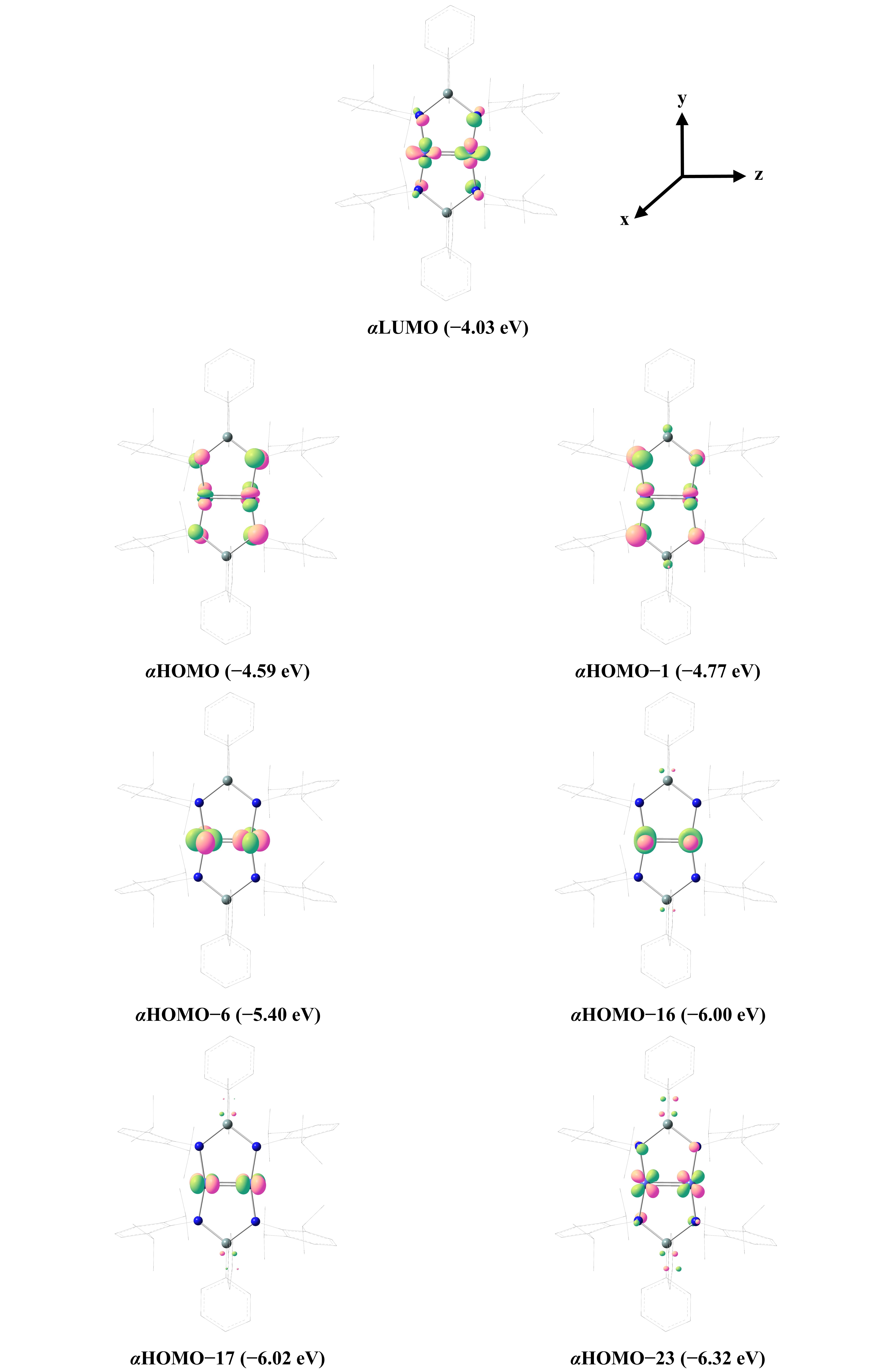


**Figure. *S*26.** The first half of FMOs of **1** plotted with an isosurface of 0.08 a.u.

**
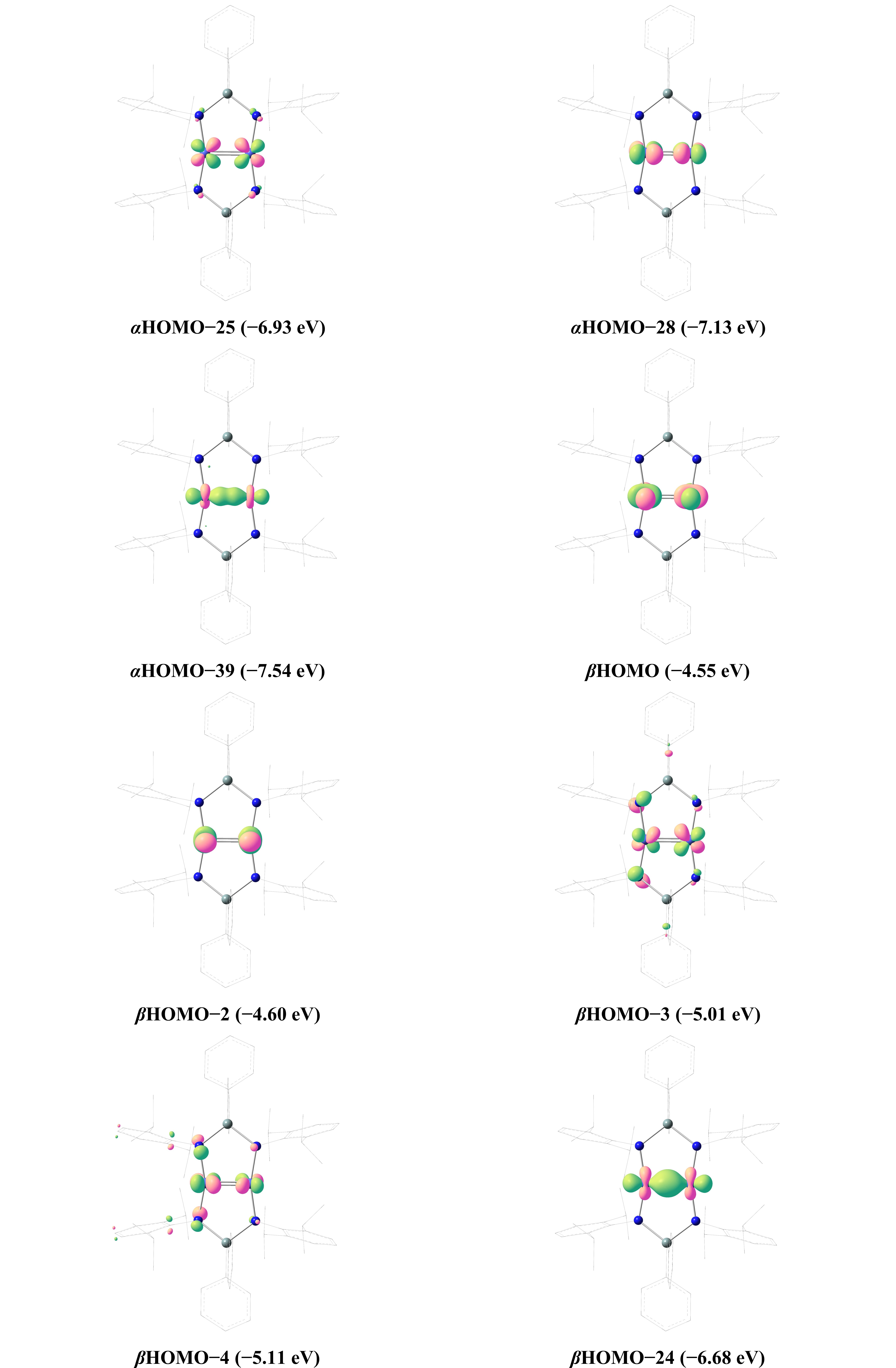
**

**Figure. *S*27.** The second half of FMOs of **1** plotted with an isosurface of 0.08 a.u.


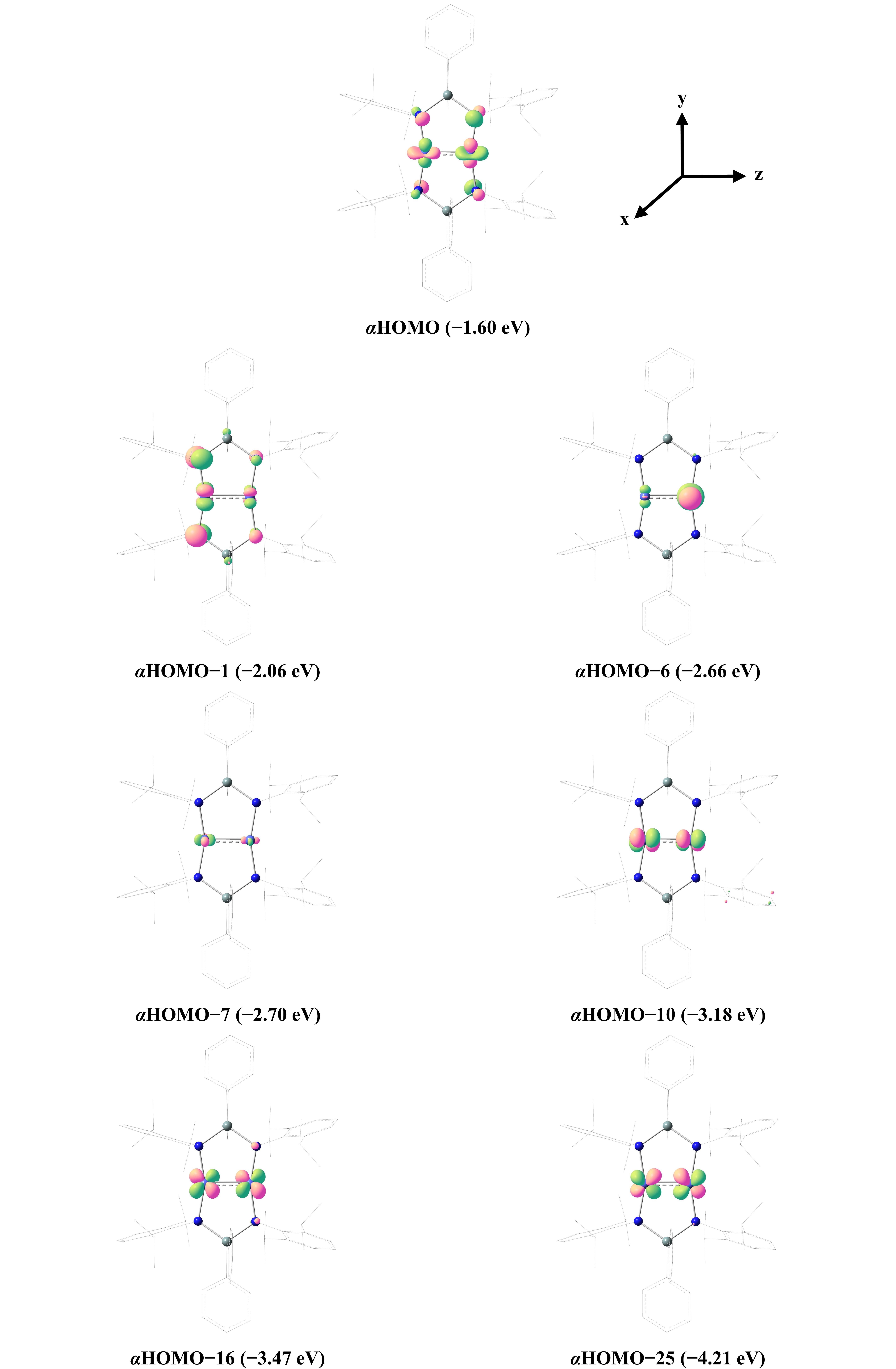


**Figure. *S*28.** The first half of FMOs of **2** plotted with an isosurface of 0.08 a.u.


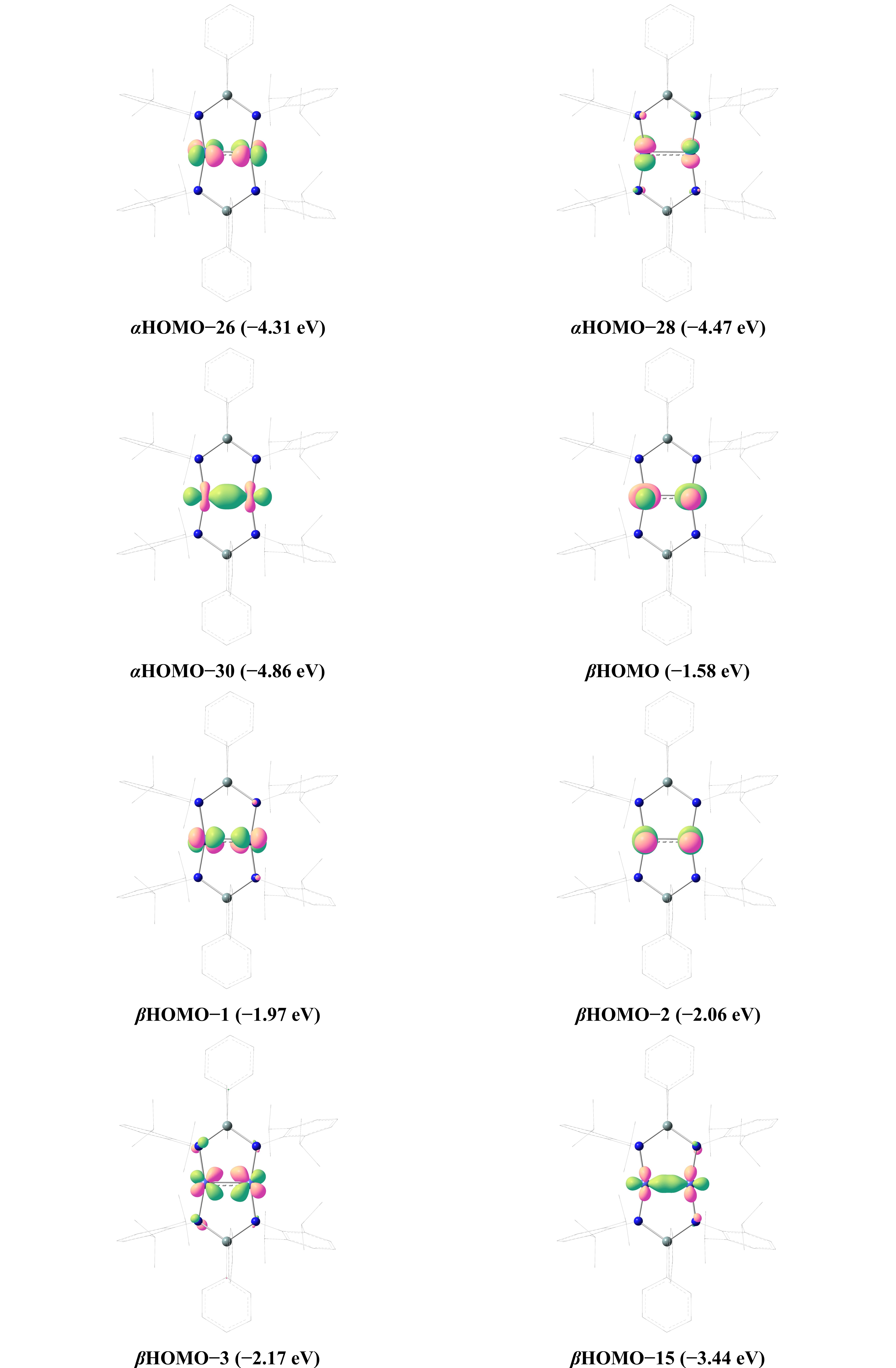


**Figure. *S*29.** The second half of FMOs of **2** plotted with an isosurface of 0.08 a.u.


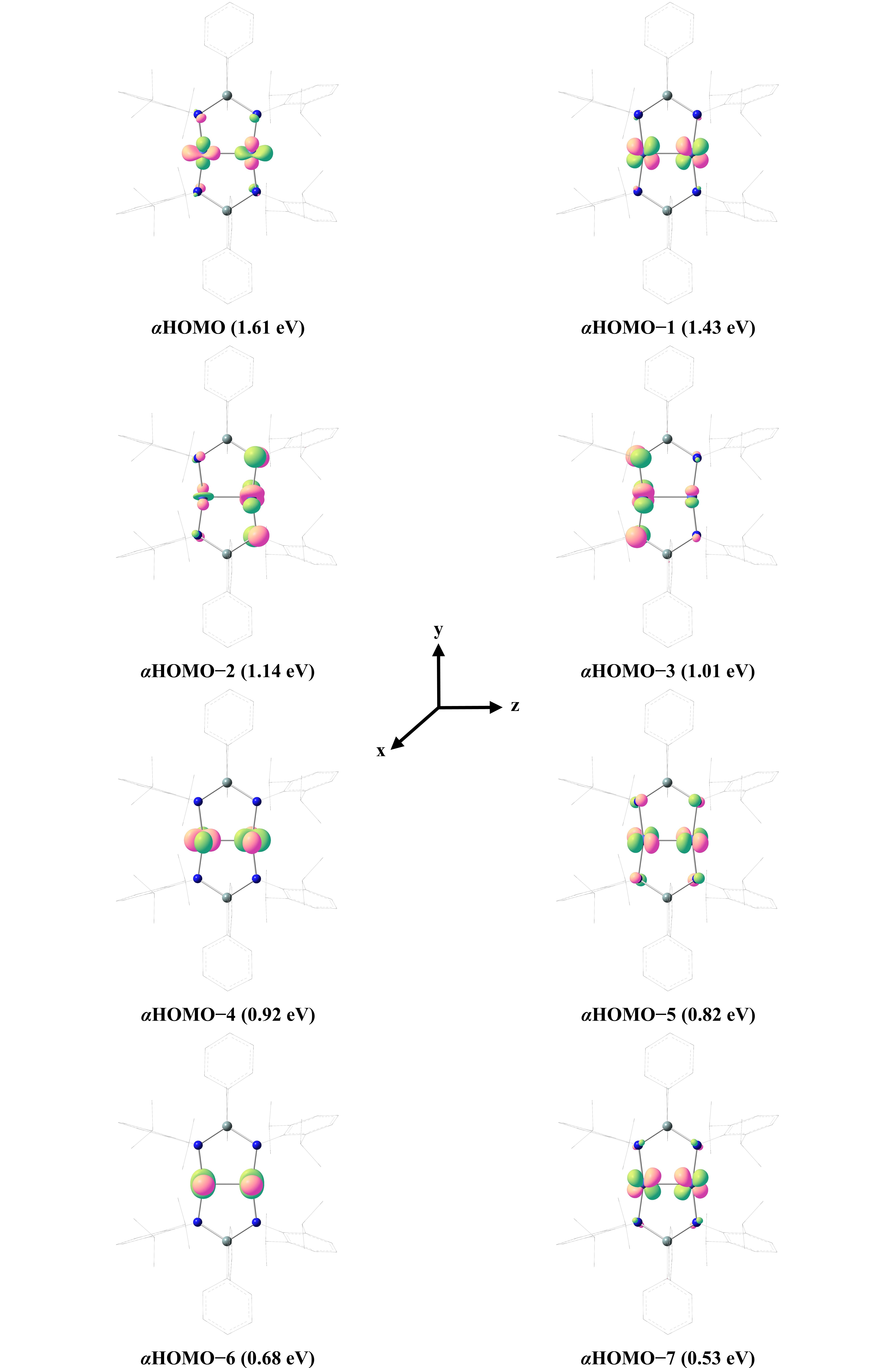


**Figure. *S*30.** The first half of FMOs of **3** plotted with an isosurface of 0.08 a.u.


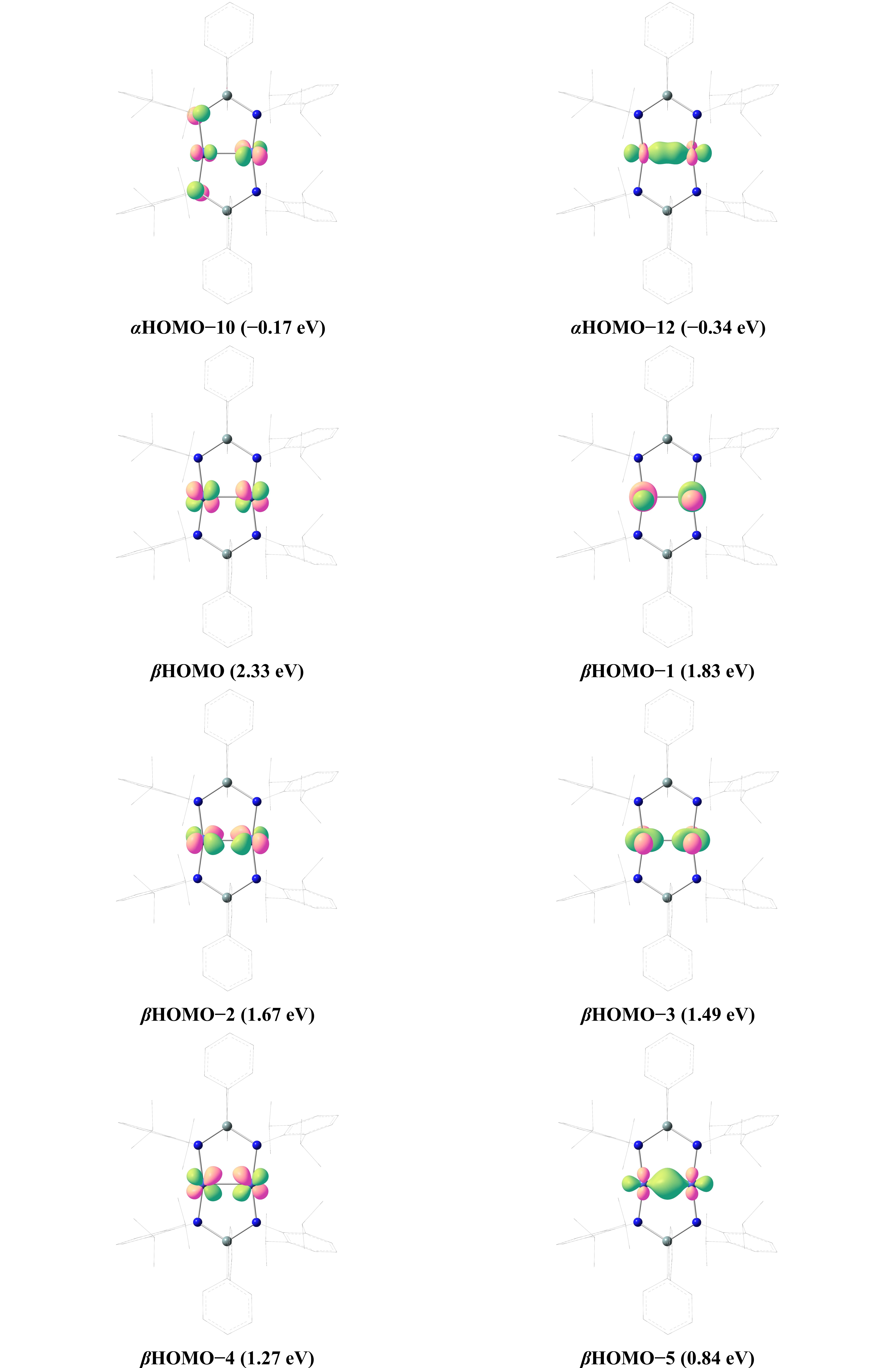


**Figure. *S*31** The second half of FMOs of **3** plotted with an isosurface of 0.08 a.u.

**
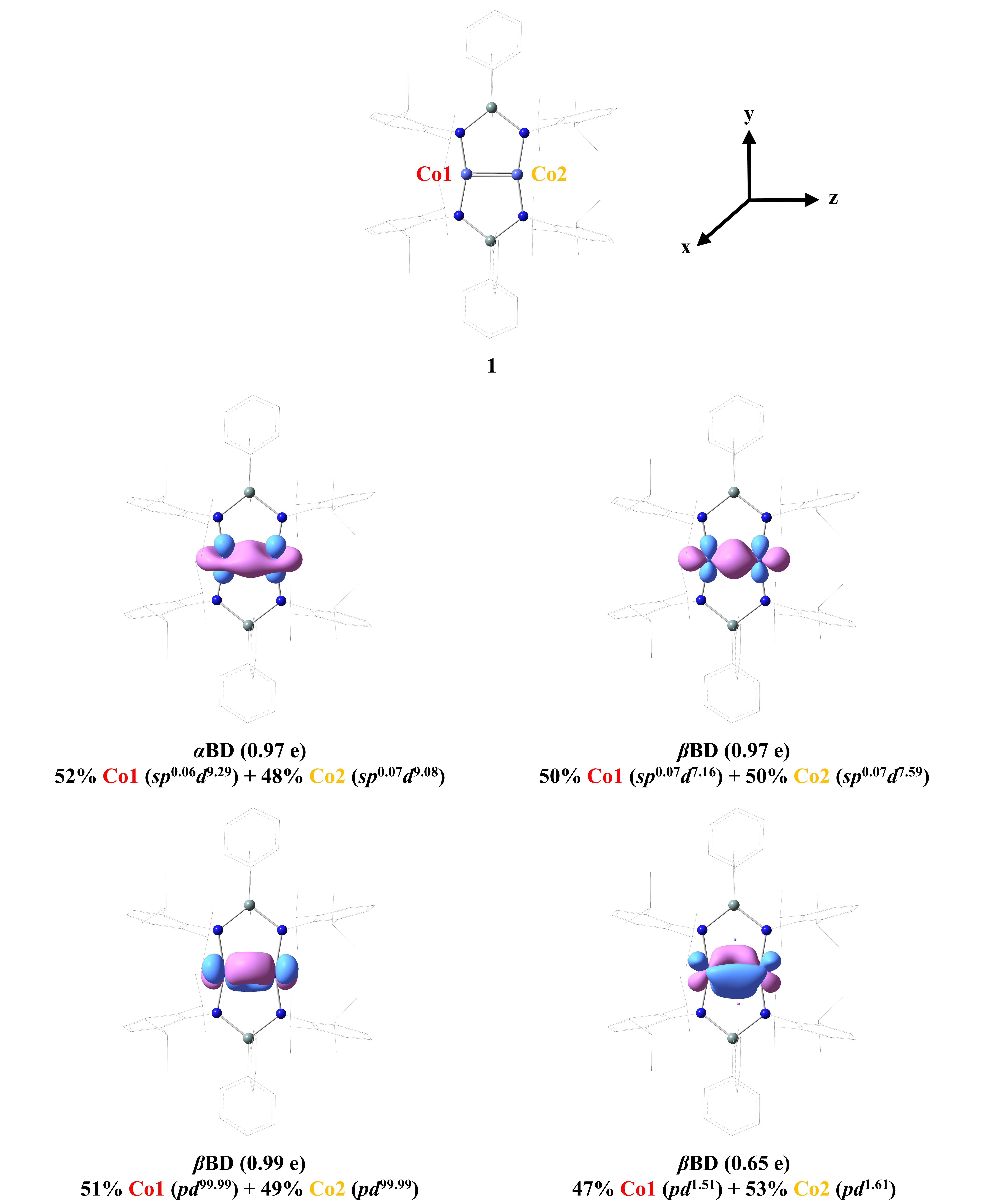
**

**Figure. *S*32** The first part of NBO analyses of **1** plotted with an isosurface of 0.05 a.u.

**
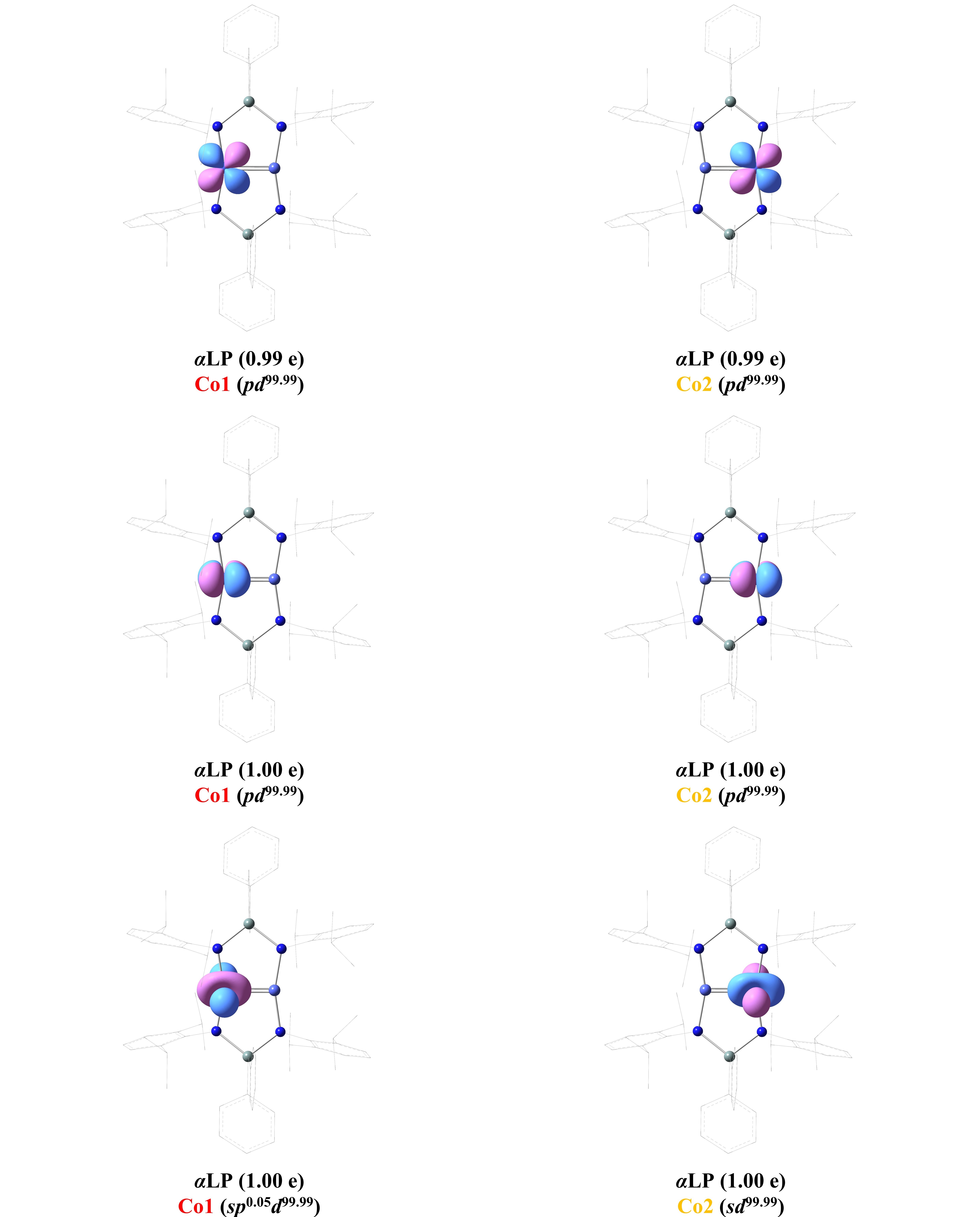
**

**Figure. *S*33** The second part of NBO analyses of **1** plotted with an isosurface of 0.05 a.u.

**
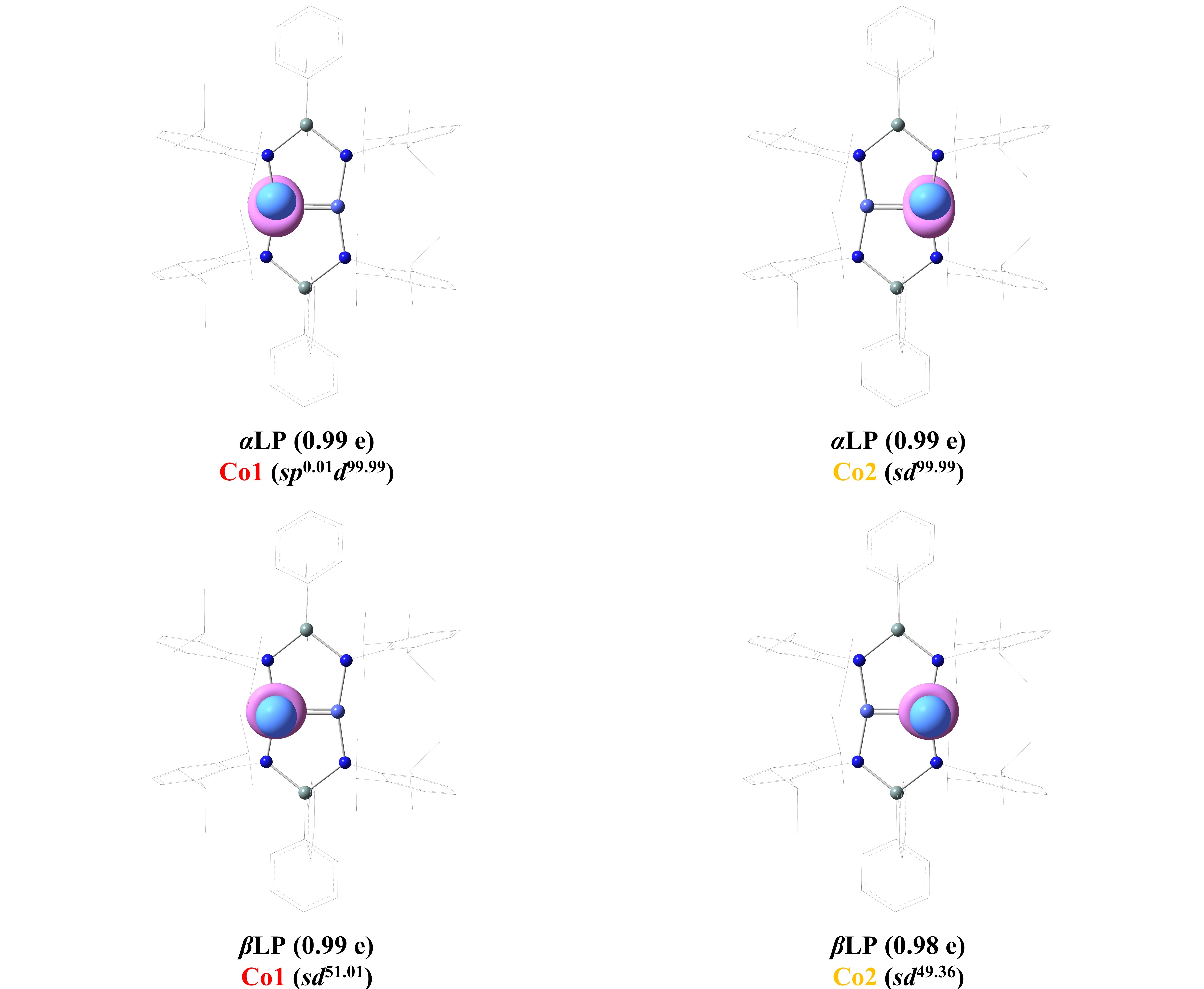
**

**Figure. *S*34** The third part of NBO analyses of **1** plotted with an isosurface of 0.05 a.u.





**Figure. *S*35** The first part of NBO analyses of **2** plotted with an isosurface of 0.05 a.u.

**
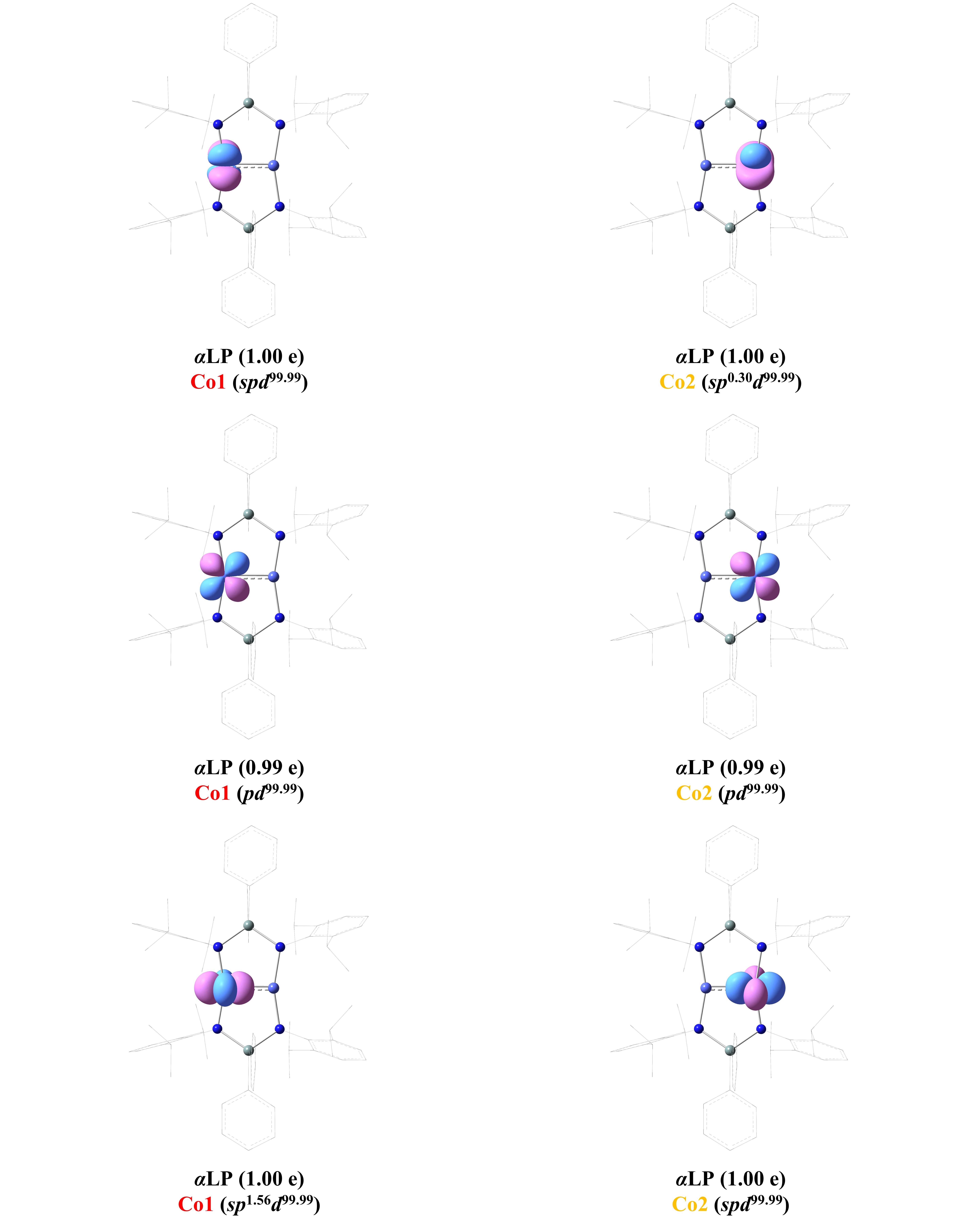
**

**Figure. *S*36** The second part of NBO analyses of **2** plotted with an isosurface of 0.05 a.u.

**
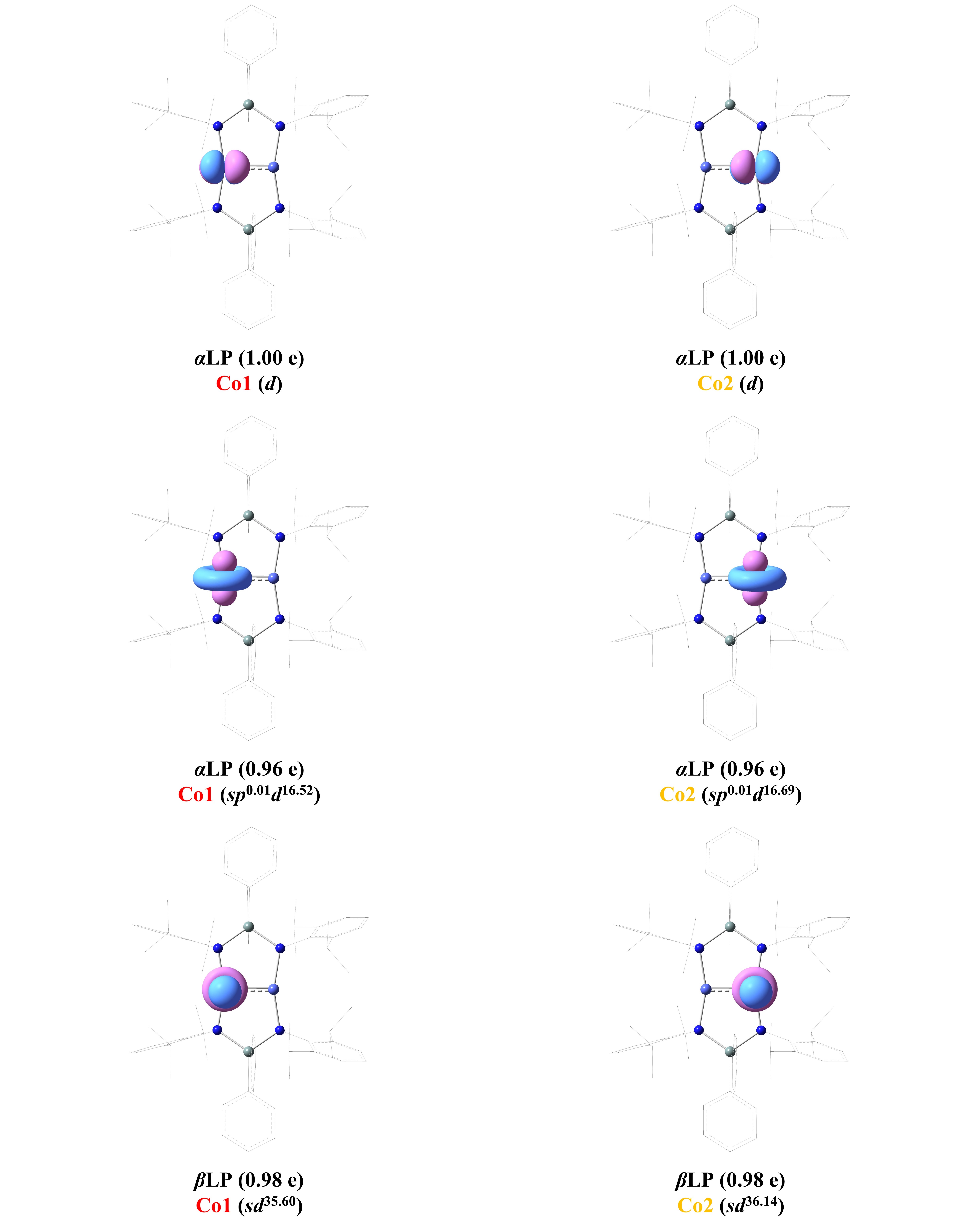
**

**Figure. *S*37** The third part of NBO analyses of **2** plotted with an isosurface of 0.05 a.u.





**Figure. *S*38** The first part of NBO analyses of **3** plotted with an isosurface of 0.05 a.u.


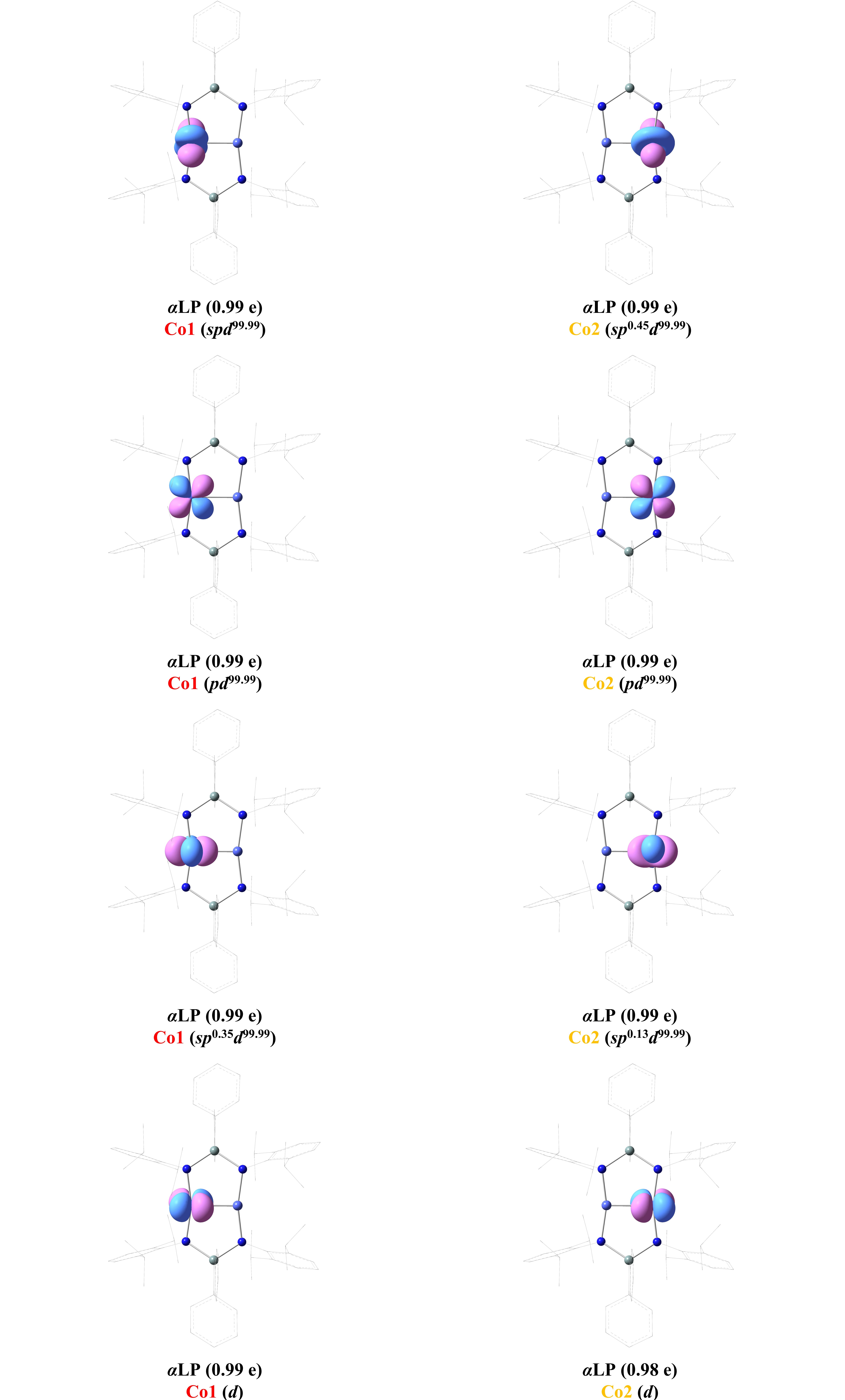


**Figure. *S*39** The second part of NBO analyses of **3** plotted with an isosurface of 0.05 a.u.


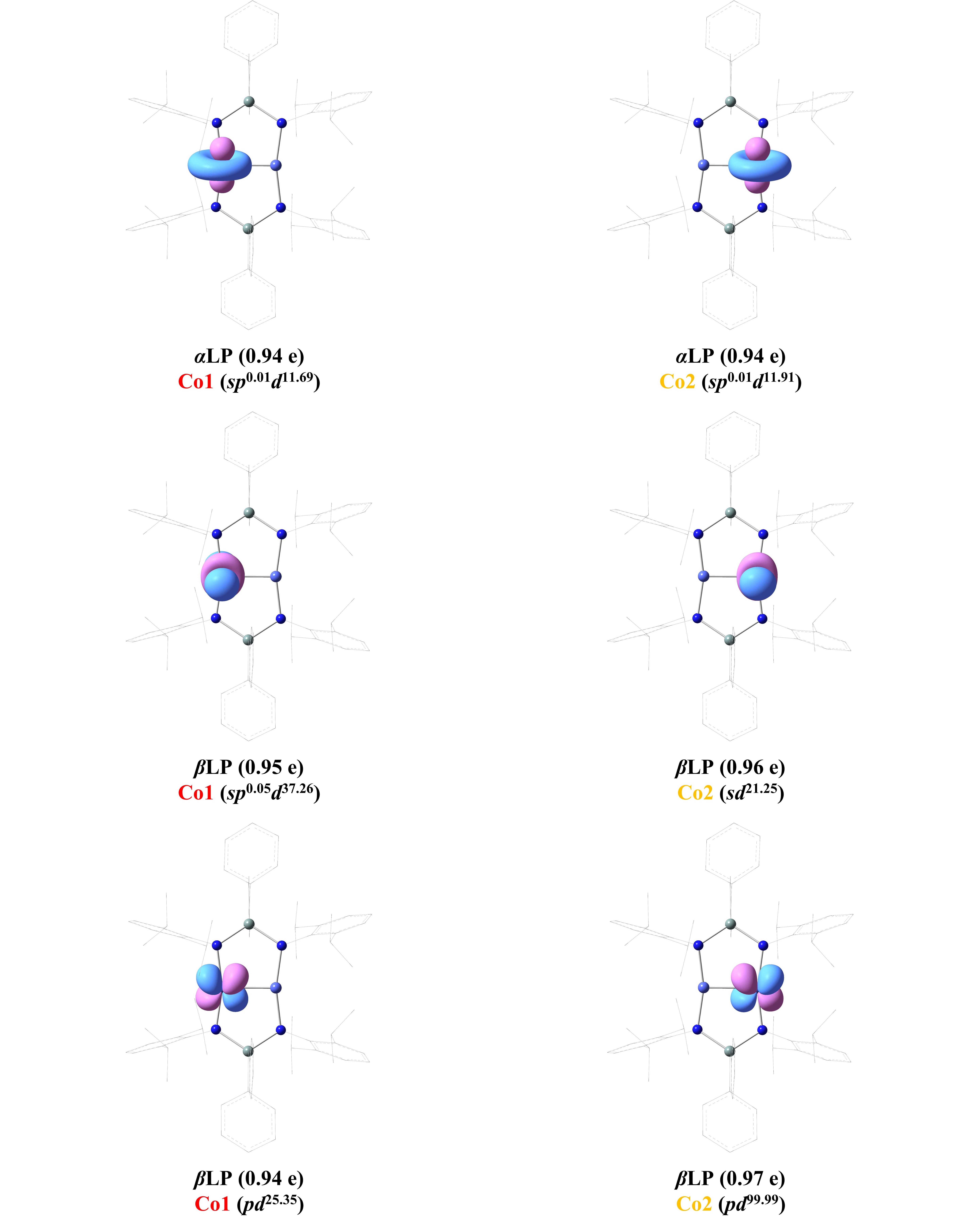


**Figure. *S*40** The third part of NBO analyses of **3** plotted with an isosurface of 0.05 a.u.

**Table *S*17.** Cartesian coordinates data sets and thermodynamic data for DFT optimized in at 1 atm and 298.15 K.

**^1^1^Me/Me^**

| Electronic Energy (UBP86) | −1409.341827 |
| --- | --- |
| Zero-point correction | 0.314684 |
| Thermal correction to Energy | 0.341572 |
| Thermal correction to Enthalpy | 0.342516 |
| Thermal correction to Gibbs Free Energy | 0.256637 |

Co 3.68981 7.2294 15.8872

Co 3.05216 9.25672 14.86815

N 4.59173 6.58131 14.48735

N 2.89875 7.52466 17.45957

N 2.14829 9.90395 16.26717

N 3.84388 8.96199 13.29598

Si 4.73602 7.47782 12.97895

Si 2.00197 9.00642 17.77479

C 2.77488 9.97316 19.21652

H 2.24994 10.92914 19.38925

H 2.72779 9.39905 20.1586

H 3.83355 10.19738 19.00435

C 0.18135 8.62706 18.1646

H -0.40091 9.55322 18.31467

H -0.27939 8.0635 17.33622

H 0.08267 8.0257 19.08545

C 1.51767 11.22345 16.13929

H 1.9129 11.9438 16.88486

H 1.69574 11.66279 15.13986

H 0.41823 11.17069 16.27869

C 3.00959 6.49386 18.49858

H 2.01508 6.1082 18.80355

H 3.59961 5.6258 18.1494

H 3.51008 6.8805 19.41007

C 5.22098 5.26107 14.61446

H 5.04317 4.82169 15.61392

H 4.82449 4.54128 13.86899

H 6.32038 5.31274 14.47432

C 3.7358 9.99426 12.2582

H 3.14701 10.86294 12.60792

H 4.7313 10.37851 11.95466

H 3.23546 9.60974 11.34571

C 6.55653 7.85201 12.58361

H 7.0213 8.41489 13.41021

H 7.1359 6.92424 12.43239

H 6.65403 8.45252 11.66208

C 3.9562 6.51265 11.53989

H 4.00187 7.08628 10.59745

H 4.47804 5.55519 11.36594

H 2.8976 6.29135 11.75551

**^3^1^Me/Me^**

| Electronic Energy (UBP86) | −1409.353827 |
| --- | --- |
| Zero-point correction | 0.314047 |
| Thermal correction to Energy | 0.341157 |
| Thermal correction to Enthalpy | 0.342101 |
| Thermal correction to Gibbs Free Energy | 0.253993 |

Co 3.6957 7.303 15.891

Co 3.0428 9.1815 14.8629

N 4.2223 6.4107 14.3413

N 3.3105 7.7657 17.6724

N 2.5163 10.0738 16.4126

N 3.4281 8.7189 13.0815

Si 4.5771 7.4385 13.0046

Si 2.1614 9.046 17.7493

C 2.09809 10.02091 19.42535

H 1.35398 10.78737 19.36425

H 1.85263 9.34806 20.22029

H 3.05274 10.465 19.61593

C 0.35455 8.36292 17.56954

H -0.17455 8.50776 18.48822

H -0.14768 8.88558 16.78247

H 0.38934 7.31868 17.33879

C 2.39736 11.53563 16.31361

H 3.36361 11.96113 16.13976

H 1.74605 11.78638 15.50255

H 1.99586 11.92344 17.22646

C 3.7744 6.83334 18.70989

H 3.30604 5.8816 18.56937

H 4.83632 6.722 18.64037

H 3.51852 7.21774 19.67512

C 4.34124 4.94888 14.4404

H 5.04379 4.69941 15.20793

H 3.38682 4.52882 14.68026

H 4.6797 4.55435 13.50515

C 2.9642 9.65126 12.04401

H 1.90429 9.77454 12.12337

H 3.44425 10.59848 12.17524

H 3.20637 9.25943 11.07824

C 6.38393 8.12164 13.18435

H 6.87817 7.61599 13.98743

H 6.92056 7.95476 12.27381

H 6.3496 9.17094 13.39094

C 4.64052 6.46363 11.32853

H 4.87618 7.13831 10.53219

H 5.39154 5.70366 11.38615

H 3.68881 6.01122 11.14283

**^5^1^Me/Me^**

| Electronic Energy (UBP86) | −1409.364539 |
| --- | --- |
| Zero-point correction | 0.313764 |
| Thermal correction to Energy | 0.341075 |
| Thermal correction to Enthalpy | 0.342019 |
| Thermal correction to Gibbs Free Energy | 0.252700 |

Co 3.6957 7.303 15.891

Co 3.0428 9.1815 14.8629

N 4.2223 6.4107 14.3413

N 3.3105 7.7657 17.6724

N 2.5163 10.0738 16.4126

N 3.4281 8.7189 13.0815

Si 4.5771 7.4385 13.0046

Si 2.1614 9.046 17.7493

C 2.09809 10.02091 19.42535

H 1.35398 10.78737 19.36425

H 1.85263 9.34806 20.22029

H 3.05274 10.465 19.61593

C 0.35455 8.36292 17.56954

H -0.17455 8.50776 18.48822

H -0.14768 8.88558 16.78247

H 0.38934 7.31868 17.33879

C 2.39736 11.53563 16.31361

H 3.36361 11.96113 16.13976

H 1.74605 11.78638 15.50255

H 1.99586 11.92344 17.22646

C 3.7744 6.83334 18.70989

H 3.30604 5.8816 18.56937

H 4.83632 6.722 18.64037

H 3.51852 7.21774 19.67512

C 4.34124 4.94888 14.4404

H 5.04379 4.69941 15.20793

H 3.38682 4.52882 14.68026

H 4.6797 4.55435 13.50515

C 2.9642 9.65126 12.04401

H 1.90429 9.77454 12.12337

H 3.44425 10.59848 12.17524

H 3.20637 9.25943 11.07824

C 6.38393 8.12164 13.18435

H 6.87817 7.61599 13.98743

H 6.92056 7.95476 12.27381

H 6.3496 9.17094 13.39094

C 4.64052 6.46363 11.32853

H 4.87618 7.13831 10.53219

H 5.39154 5.70366 11.38615

H 3.68881 6.01122 11.14283

**^7^1^Me/Me^**

| Electronic Energy (UBP86) | −1409.363858 |
| --- | --- |
| Zero-point correction | 0.313567 |
| Thermal correction to Energy | 0.341132 |
| Thermal correction to Enthalpy | 0.342076 |
| Thermal correction to Gibbs Free Energy | 0.241509 |

Co 3.6957 7.303 15.891

Co 3.0428 9.1815 14.8629

N 4.2223 6.4107 14.3413

N 3.3105 7.7657 17.6724

N 2.5163 10.0738 16.4126

N 3.4281 8.7189 13.0815

Si 4.5771 7.4385 13.0046

Si 2.1614 9.046 17.7493

C 2.09809 10.02091 19.42535

H 1.35398 10.78737 19.36425

H 1.85263 9.34806 20.22029

H 3.05274 10.465 19.61593

C 0.35455 8.36292 17.56954

H -0.17455 8.50776 18.48822

H -0.14768 8.88558 16.78247

H 0.38934 7.31868 17.33879

C 2.39736 11.53563 16.31361

H 3.36361 11.96113 16.13976

H 1.74605 11.78638 15.50255

H 1.99586 11.92344 17.22646

C 3.7744 6.83334 18.70989

H 3.30604 5.8816 18.56937

H 4.83632 6.722 18.64037

H 3.51852 7.21774 19.67512

C 4.34124 4.94888 14.4404

H 5.04379 4.69941 15.20793

H 3.38682 4.52882 14.68026

H 4.6797 4.55435 13.50515

C 2.9642 9.65126 12.04401

H 1.90429 9.77454 12.12337

H 3.44425 10.59848 12.17524

H 3.20637 9.25943 11.07824

C 6.38393 8.12164 13.18435

H 6.87817 7.61599 13.98743

H 6.92056 7.95476 12.27381

H 6.3496 9.17094 13.39094

C 4.64052 6.46363 11.32853

H 4.87618 7.13831 10.53219

H 5.39154 5.70366 11.38615

H 3.68881 6.01122 11.14283

**^9^1^Me/Me^**

| Electronic Energy (UBP86) | −1409.274727 |
| --- | --- |
| Zero-point correction | 0.312160 |
| Thermal correction to Energy | 0.339858 |
| Thermal correction to Enthalpy | 0.340802 |
| Thermal correction to Gibbs Free Energy | 0.249829 |

Co 3.68981 7.2294 15.8872

Co 3.05216 9.25672 14.86815

N 4.59173 6.58131 14.48735

N 2.89875 7.52466 17.45957

N 2.14829 9.90395 16.26717

N 3.84388 8.96199 13.29598

Si 4.73602 7.47782 12.97895

Si 2.00197 9.00642 17.77479

C 2.77488 9.97316 19.21652

H 2.24994 10.92914 19.38925

H 2.72779 9.39905 20.1586

H 3.83355 10.19738 19.00435

C 0.18135 8.62706 18.1646

H -0.40091 9.55322 18.31467

H -0.27939 8.0635 17.33622

H 0.08267 8.0257 19.08545

C 1.51767 11.22345 16.13929

H 1.9129 11.9438 16.88486

H 1.69574 11.66279 15.13986

H 0.41823 11.17069 16.27869

C 3.00959 6.49386 18.49858

H 2.01508 6.1082 18.80355

H 3.59961 5.6258 18.1494

H 3.51008 6.8805 19.41007

C 5.22098 5.26107 14.61446

H 5.04317 4.82169 15.61392

H 4.82449 4.54128 13.86899

H 6.32038 5.31274 14.47432

C 3.7358 9.99426 12.2582

H 3.14701 10.86294 12.60792

H 4.7313 10.37851 11.95466

H 3.23546 9.60974 11.34571

C 6.55653 7.85201 12.58361

H 7.0213 8.41489 13.41021

H 7.1359 6.92424 12.43239

H 6.65403 8.45252 11.66208

C 3.9562 6.51265 11.53989

H 4.00187 7.08628 10.59745

H 4.47804 5.55519 11.36594

H 2.8976 6.29135 11.75551

**^5^1^Dipp/Ph^**

| Electronic Energy (UBP86) | −3886.740659 |
| --- | --- |
| Zero-point correction | 1.387516 |
| Thermal correction to Energy | 1.474049 |
| Thermal correction to Enthalpy | 1.474993 |
| Thermal correction to Gibbs Free Energy | 1.262411 |

Co 3.6957 7.303 15.891

Co 3.0428 9.1815 14.8629

N 4.2223 6.4107 14.3413

N 3.3105 7.7657 17.6724

H 1.9032 5.7363 17.2529

H 6.2098 6.1824 15.7891

N 2.5163 10.0738 16.4126

N 3.4281 8.7189 13.0815

H 4.8353 10.7483 13.501

H 0.5287 10.3021 14.9649

Si 4.5771 7.4385 13.0046

C 4.3368 5.0035 14.4367

Si 2.1614 9.046 17.7493

C 3.7584 6.8655 18.6741

C 2.0263 5.0791 17.9957

C 6.535 5.2383 15.7352

C 2.4018 11.4811 16.3173

C 2.9802 9.6191 12.0798

C 4.7123 11.4055 12.7583

C 0.2036 11.2463 15.0187

C 4.6389 6.4885 11.3713

C 6.3513 8.1093 13.1811

C 5.4232 4.4018 15.1017

C 3.3263 4.1688 13.8808

C 2.0997 9.9961 19.3827

C 0.3873 8.3753 17.5728

C 4.8854 7.2166 19.4841

C 3.154 5.5946 18.8737

C 0.6912 4.9792 18.743

C 2.3829 3.735 17.3621

C 7.7788 5.2267 14.8403

C 6.8796 4.7864 17.1515

C 1.3153 12.0827 15.6522

C 3.4123 12.3157 16.8731

C 1.8532 9.2679 11.2698

C 3.5846 10.89 11.8802

C 6.0474 11.5054 12.0109

C 4.3557 12.7496 13.3918

C -1.0403 11.2579 15.9136

C -0.1411 11.6982 13.6025

C 5.2403 5.2262 11.3113

C 4.1519 6.978 10.1626

C 7.3138 7.9582 12.1678

C 6.7586 8.7465 14.3498

C 5.5105 3.0165 15.174

C 3.4615 2.7864 13.973

C 2.0894 4.7515 13.2334

C 1.4983 11.2584 19.4426

C 2.5866 9.5066 20.5913

C -0.5753 8.5264 18.5861

C -0.02 7.7381 16.4041

C 5.3154 6.326 20.456

C 5.6337 8.5201 19.2535

C 3.6348 4.7632 19.8824

H 0.457 5.8558 19.112

H -0.0092 4.6871 18.1233

H 0.773 4.3297 19.4734

H 2.4762 3.0575 18.0648

H 1.6716 3.4671 16.744

H 3.2276 3.8189 16.8731

H 8.1125 4.3094 14.7573

H 8.4743 5.7918 15.2401

H 7.5473 5.5718 13.9531

H 6.0718 4.8068 17.7066

H 7.5522 5.3881 17.5313

H 7.2348 3.8738 17.1253

C 1.2281 13.4681 15.5799

C 3.2771 13.6982 16.7809

C 4.6492 11.7331 17.5205

C 1.4232 10.1586 10.2979

C 1.1048 7.9645 11.5004

C 3.1037 11.7213 10.8715

H 6.2816 10.6287 11.6419

H 6.7478 11.7975 12.6306

H 5.9656 12.1549 11.2805

H 4.2624 13.4271 12.6891

H 5.067 13.0175 14.0099

H 3.5109 12.6657 13.8808

H -1.3739 12.1752 15.9967

H -1.7357 10.6928 15.5138

H -0.8088 10.9127 16.8009

H 0.6668 11.6778 13.0473

H -0.8137 11.0965 13.2226

H -0.4963 12.6107 13.6286

H 5.5579 4.8329 12.1155

C 5.389 4.5396 10.1411

C 4.2657 6.2298 8.9801

H 3.7358 7.8316 10.138

H 7.0727 7.5355 11.3513

C 8.6116 8.4161 12.3431

C 8.0594 9.1989 14.5282

H 6.1263 8.8752 15.0479

H 6.2469 2.6204 15.6245

C 4.5438 2.1984 14.6004

H 2.7911 2.2302 13.5932

H 2.2681 5.7222 13.0766

C 1.7791 4.122 11.8802

C 0.8772 4.6571 14.1483

H 1.1807 11.6517 18.6384

C 1.3496 11.9449 20.6128

C 2.4729 10.2547 21.7738

H 3.0028 8.6529 20.6159

H -0.3341 8.9491 19.4026

C -1.8731 8.0684 18.4108

C -1.3208 7.2857 16.2258

H 0.6123 7.6093 15.706

H 6.0635 6.5649 20.9911

C 4.7066 5.115 20.6805

H 4.9902 9.1837 18.8721

C 6.2128 9.1051 20.5313

C 6.7498 8.3127 18.2478

H 3.2095 3.9248 20.027

H 0.4916 13.8642 15.1294

C 2.1948 14.2862 16.1535

H 3.9475 14.2544 17.1607

H 4.4705 10.7624 17.6774

C 4.9595 12.3626 18.8737

C 5.8614 11.8275 16.6056

H 0.6751 9.9196 9.7628

C 2.032 11.3696 10.0734

H 1.7484 7.3009 11.8818

C 0.5258 7.3795 10.2226

C -0.0112 8.1719 12.5061

H 3.5291 12.5598 10.727

H 5.834 3.7004 10.1334

C 4.8923 5.0684 8.9709

H 3.8854 6.5644 8.1759

H 9.2464 8.3101 11.645

C 8.9888 9.0233 13.5194

H 8.3088 9.6269 15.3401

H 4.6255 1.2522 14.6389

H 1.6759 3.1534 11.9863

H 2.5133 4.3071 11.2575

H 0.9476 4.5023 11.5266

H 0.7581 3.7287 14.439

H 0.0779 4.9521 13.6624

H 1.0129 5.2304 14.931

H 0.9045 12.7841 20.6205

C 1.8462 11.4162 21.783

H 2.8531 9.9202 22.578

H -2.5078 8.1744 19.1089

C -2.2502 7.4612 17.2345

H -1.5702 6.8577 15.4139

H 5.0139 4.5344 21.3663

H 6.9699 8.5581 20.8266

H 6.5177 10.0213 20.3622

H 5.5234 9.1139 21.2279

H 7.397 7.6697 18.6061

H 6.376 7.9685 17.4113

H 7.1987 9.1676 18.0772

H 2.1131 15.2323 16.1151

H 5.0627 13.3312 18.7676

H 4.2253 12.1774 19.4964

H 5.791 11.9823 19.2274

H 5.9805 12.7558 16.315

H 6.6607 11.5324 17.0915

H 5.7257 11.2541 15.8229

H 1.7246 11.9502 9.3876

H -0.2313 7.9264 9.9274

H 0.2209 6.4633 10.3917

H 1.2152 7.3707 9.526

H -0.6585 8.8149 12.1478

H 0.3626 8.5161 13.3426

H -0.4601 7.3169 12.6768

H 5.0019 4.5954 8.1544

H 9.8825 9.3209 13.6378

H 1.7367 11.8892 22.5995

H -3.144 7.1637 17.1161

**^2^2^Me/Me^**

| Electronic Energy (UBP86) | −1409.416862 |
| --- | --- |
| Zero-point correction | 0.310349 |
| Thermal correction to Energy | 0.338337 |
| Thermal correction to Enthalpy | 0.339281 |
| Thermal correction to Gibbs Free Energy | 0.249090 |

Co 0.34135 -1.0624 0.58135

Co -0.31919 1.0159 -0.55295

N 1.28522 -1.70093 -0.8725

N -0.51832 -0.68202 2.17154

N -1.2728 1.68598 0.88333

N 0.52911 0.66879 -2.15988

Si 1.4181 -0.81827 -2.36035

Si -1.40426 0.79574 2.37849

C -0.71783 1.8307 3.84546

H -1.26722 2.7811 3.98072

H -0.77761 1.27978 4.80261

H 0.34317 2.07512 3.6629

C -3.24764 0.45951 2.80658

H -3.3549 -0.117 3.74432

H -3.82613 1.39415 2.92974

H -3.71628 -0.12526 1.99589

C -1.92662 2.98809 0.75615

H -1.55678 3.73827 1.49354

H -1.75621 3.43382 -0.24569

H -3.03288 2.93779 0.88635

C -0.4461 -1.64612 3.26839

H -1.44421 -2.03492 3.58093

H 0.15257 -2.5353 2.98397

H 0.03362 -1.23398 4.18767

C 1.93827 -3.00441 -0.74228

H 3.04575 -2.95708 -0.86823

H 1.76526 -3.44631 0.26029

H 1.56776 -3.7583 -1.47643

C 0.45926 1.62963 -3.25883

H -0.13872 2.52218 -2.98185

H 1.45896 2.01296 -3.57072

H -0.01681 1.21235 -4.17689

C 3.25354 -0.45675 -2.80197

H 3.84152 -1.38618 -2.91881

H 3.35211 0.11281 -3.74504

H 3.71906 0.13755 -1.99642

C 0.72477 -1.8283 -3.84148

H 0.7962 -1.27334 -4.79563

H 1.26383 -2.78425 -3.97856

H -0.33924 -2.0637 -3.66468

**^4^2^Me/Me^**

| Electronic Energy (UBP86) | −1409.412770 |
| --- | --- |
| Zero-point correction | 0.310778 |
| Thermal correction to Energy | 0.338345 |
| Thermal correction to Enthalpy | 0.339289 |
| Thermal correction to Gibbs Free Energy | 0.250177 |

Co 3.6957 7.303 15.891

Co 3.0428 9.1815 14.8629

N 4.2223 6.4107 14.3413

N 3.3105 7.7657 17.6724

N 2.5163 10.0738 16.4126

N 3.4281 8.7189 13.0815

Si 4.5771 7.4385 13.0046

Si 2.1614 9.046 17.7493

C 2.09809 10.02091 19.42535

H 1.35398 10.78737 19.36425

H 1.85263 9.34806 20.22029

H 3.05274 10.465 19.61593

C 0.35456 8.36292 17.56954

H -0.17455 8.50776 18.48822

H -0.14768 8.88558 16.78247

H 0.38934 7.31868 17.33879

C 2.39736 11.53563 16.31361

H 3.36361 11.96113 16.13976

H 1.74605 11.78638 15.50255

H 1.99586 11.92344 17.22646

C 3.7744 6.83333 18.70989

H 3.30604 5.8816 18.56937

H 4.83632 6.722 18.64037

H 3.51852 7.21774 19.67512

C 4.34124 4.94888 14.4404

H 5.04379 4.69941 15.20793

H 3.38682 4.52882 14.68026

H 4.6797 4.55435 13.50515

C 2.9642 9.65127 12.04401

H 1.90429 9.77454 12.12337

H 3.44426 10.59848 12.17524

H 3.20637 9.25943 11.07824

C 6.38393 8.12163 13.18435

H 6.87817 7.61599 13.98743

H 6.92056 7.95476 12.27381

H 6.3496 9.17094 13.39094

C 4.64052 6.46363 11.32853

H 4.87618 7.13831 10.53219

H 5.39154 5.70366 11.38615

H 3.68881 6.01122 11.14283

**^6^2^Me/Me^**

| Electronic Energy (UBP86) | −1409.437604 |
| --- | --- |
| Zero-point correction | 0.310765 |
| Thermal correction to Energy | 0.338708 |
| Thermal correction to Enthalpy | 0.339652 |
| Thermal correction to Gibbs Free Energy | 0.248223 |

Co 0.32067 -1.0237 0.56754

Co -0.33558 1.04176 -0.58182

N 1.26096 -1.6854 -0.88001

N -0.51449 -0.68872 2.18547

N -1.27181 1.66992 0.88181

N 0.51629 0.67833 -2.18246

Si 1.40932 -0.79723 -2.37502

Si -1.40232 0.79896 2.37711

C -0.71319 1.82433 3.85003

H -1.27395 2.76361 3.99993

H -0.75408 1.26493 4.80118

H 0.33961 2.08845 3.65892

C -3.23697 0.43657 2.82004

H -3.33239 -0.17703 3.74034

H -3.81786 1.36756 2.98641

H -3.71725 -0.11836 1.99046

C -1.92079 2.97485 0.74447

H -1.52984 3.73912 1.45694

H -1.76817 3.39848 -0.26948

H -3.02529 2.93435 0.89473

C -0.44413 -1.64444 3.28828

H -1.44831 -1.94613 3.67137

H 0.06175 -2.58298 2.9806

H 0.12379 -1.25875 4.16812

C 1.90891 -2.99003 -0.74579

H 3.00731 -2.94614 -0.8591

H 1.72785 -3.43542 0.24298

H 1.55188 -3.73344 -1.48089

C 0.45581 1.63971 -3.28226

H -0.11663 2.54537 -2.99566

H 1.45969 2.00189 -3.60894

H -0.04407 1.23595 -4.19482

C 3.2566 -0.45249 -2.77945

H 3.84709 -1.38186 -2.87124

H 3.37614 0.10369 -3.7262

H 3.70364 0.1538 -1.97433

C 0.74664 -1.83878 -3.84843

H 0.81552 -1.2896 -4.80507

H 1.30181 -2.78573 -3.97558

H -0.31448 -2.08797 -3.67916

**^8^2^Me/Me^**

| Electronic Energy (UBP86) | −1409.358012 |
| --- | --- |
| Zero-point correction | 0.311710 |
| Thermal correction to Energy | 0.339575 |
| Thermal correction to Enthalpy | 0.340519 |
| Thermal correction to Gibbs Free Energy | 0.249172 |

Co 0.32067 -1.0237 0.56754

Co -0.33558 1.04176 -0.58182

N 1.26096 -1.6854 -0.88001

N -0.51449 -0.68872 2.18547

N -1.27181 1.66992 0.88181

N 0.51629 0.67833 -2.18246

Si 1.40932 -0.79723 -2.37502

Si -1.40232 0.79896 2.37711

C -0.71319 1.82433 3.85003

H -1.27395 2.76361 3.99993

H -0.75408 1.26493 4.80118

H 0.33961 2.08845 3.65892

C -3.23697 0.43657 2.82004

H -3.33239 -0.17703 3.74034

H -3.81786 1.36756 2.98641

H -3.71725 -0.11836 1.99046

C -1.92079 2.97485 0.74447

H -1.52984 3.73912 1.45694

H -1.76817 3.39848 -0.26948

H -3.02529 2.93435 0.89473

C -0.44413 -1.64444 3.28828

H -1.44831 -1.94613 3.67137

H 0.06175 -2.58298 2.9806

H 0.12379 -1.25875 4.16812

C 1.90891 -2.99003 -0.74579

H 3.00731 -2.94614 -0.8591

H 1.72785 -3.43542 0.24298

H 1.55188 -3.73344 -1.48089

C 0.45581 1.63971 -3.28226

H -0.11663 2.54537 -2.99566

H 1.45969 2.00189 -3.60894

H -0.04407 1.23595 -4.19482

C 3.2566 -0.45249 -2.77945

H 3.84709 -1.38186 -2.87124

H 3.37614 0.10369 -3.7262

H 3.70364 0.1538 -1.97433

C 0.74664 -1.83878 -3.84843

H 0.81552 -1.2896 -4.80507

H 1.30181 -2.78573 -3.97558

H -0.31448 -2.08797 -3.67916

**^10^2^Me/Me^**

| Electronic Energy (UBP86) | −1409.268106 |
| --- | --- |
| Zero-point correction | 0.311048 |
| Thermal correction to Energy | 0.338899 |
| Thermal correction to Enthalpy | 0.339844 |
| Thermal correction to Gibbs Free Energy | 0.248690 |

Co 0.32067 -1.0237 0.56754

Co -0.33558 1.04176 -0.58182

N 1.26096 -1.6854 -0.88001

N -0.51449 -0.68872 2.18547

N -1.27181 1.66992 0.88181

N 0.51629 0.67833 -2.18246

Si 1.40932 -0.79723 -2.37502

Si -1.40232 0.79896 2.37711

C -0.71319 1.82433 3.85003

H -1.27395 2.76361 3.99993

H -0.75408 1.26493 4.80118

H 0.33961 2.08845 3.65892

C -3.23697 0.43657 2.82004

H -3.33239 -0.17703 3.74034

H -3.81786 1.36756 2.98641

H -3.71725 -0.11836 1.99046

C -1.92079 2.97485 0.74447

H -1.52984 3.73912 1.45694

H -1.76817 3.39848 -0.26948

H -3.02529 2.93435 0.89473

C -0.44413 -1.64444 3.28828

H -1.44831 -1.94613 3.67137

H 0.06175 -2.58298 2.9806

H 0.12379 -1.25875 4.16812

C 1.90891 -2.99003 -0.74579

H 3.00731 -2.94614 -0.8591

H 1.72785 -3.43542 0.24298

H 1.55188 -3.73344 -1.48089

C 0.45581 1.63971 -3.28226

H -0.11663 2.54537 -2.99566

H 1.45969 2.00189 -3.60894

H -0.04407 1.23595 -4.19482

C 3.2566 -0.45249 -2.77945

H 3.84709 -1.38186 -2.87124

H 3.37614 0.10369 -3.7262

H 3.70364 0.1538 -1.97433

C 0.74664 -1.83878 -3.84843

H 0.81552 -1.2896 -4.80507

H 1.30181 -2.78573 -3.97558

H -0.31448 -2.08797 -3.67916

**^6^2^Dipp/Ph^**

| Electronic Energy (UBP86) | −3886.846072 |
| --- | --- |
| Zero-point correction | 1.384980 |
| Thermal correction to Energy | 1.471840 |
| Thermal correction to Enthalpy | 1.472784 |
| Thermal correction to Gibbs Free Energy | 1.258298 |

Co 3.63844 7.29864 15.84656

Co 3.10067 9.18092 14.90853

N 4.30397 6.40988 14.38171

N 3.1216 7.66424 17.5716

H 1.37499 5.91502 17.74567

H 6.62642 6.14804 15.20794

N 2.40441 10.03501 16.37902

N 3.66266 8.80773 13.19967

H 5.08348 10.79274 13.62138

H 0.39352 10.10876 14.93332

Si 4.8 7.45151 13.04517

C 4.33066 4.99043 14.56634

Si 1.95448 8.97891 17.73509

C 3.74778 6.7996 18.52546

C 1.65468 5.25981 18.58859

C 6.84218 5.06598 15.23273

C 2.24672 11.45239 16.28107

C 3.1698 9.66321 12.16598

C 4.91023 11.50027 12.79124

C 0.06408 11.16173 14.8855

C 4.80703 6.61594 11.33832

C 6.57085 8.12435 13.26603

C 5.522 4.32866 15.02576

C 3.15239 4.20522 14.31212

C 1.94265 9.83186 19.4335

C 0.15903 8.3926 17.47167

C 5.06421 7.10093 19.02142

C 3.08521 5.61022 18.98862

C 0.68262 5.55397 19.75644

C 1.50673 3.79331 18.12787

C 7.81992 4.7544 14.07417

C 7.50159 4.73738 16.58993

C 1.14924 12.01373 15.53994

C 3.18006 12.33873 16.92603

C 2.10855 9.22529 11.29713

C 3.73147 10.97362 11.97583

C 6.19655 11.53564 11.93339

C 4.62892 12.89242 13.39695

C -1.26453 11.26966 15.6696

C -0.15498 11.53006 13.40213

C 4.8872 5.21093 11.17774

C 4.86857 7.41217 10.16573

C 7.57685 7.9025 12.29481

C 6.92495 8.88656 14.40568

C 5.49799 2.93687 15.22886

C 3.18802 2.81863 14.55032

C 1.86162 4.79863 13.74961

C 1.29581 11.08508 19.58805

C 2.44225 9.20716 20.60249

C -0.8361 8.57599 18.46194

C -0.23484 7.77073 16.26205

C 5.67847 6.20962 19.9208

C 5.82268 8.37455 18.65091

C 3.74685 4.76781 19.90045

H 0.75679 6.59982 20.0969

H -0.36122 5.37354 19.44738

H 0.9038 4.90195 20.62087

H 1.70802 3.08445 18.95054

H 0.47434 3.60698 17.78492

H 2.19285 3.5545 17.29871

H 8.09082 3.683 14.07051

H 8.74698 5.34351 14.17988

H 7.37988 4.99286 13.09197

H 6.82353 4.94414 17.43418

H 8.41359 5.34434 16.72417

H 7.80576 3.67736 16.65015

C 1.02253 13.41395 15.4608

C 3.00606 13.72719 16.79717

C 4.38023 11.82264 17.71626

C 1.6359 10.09683 10.30132

C 1.44762 7.857 11.44504

C 3.22276 11.79366 10.9503

H 6.44276 10.54099 11.52802

H 7.05478 11.87158 12.54041

H 6.08441 12.2344 11.08469

H 4.49946 13.65885 12.6122

H 5.47853 13.21033 14.02487

H 3.7224 12.89253 14.02334

H -1.6475 12.30611 15.65457

H -2.03136 10.61644 15.21884

H -1.14204 10.96475 16.7214

H 0.77558 11.45115 12.81738

H -0.90104 10.85417 12.95072

H -0.53919 12.55967 13.29129

H 4.86238 4.55894 12.05696

C 5.00866 4.62605 9.90516

C 4.98818 6.83114 8.89248

H 4.82668 8.50431 10.2436

H 7.34435 7.31737 11.39894

C 8.87397 8.42103 12.45055

C 8.21697 9.40958 14.56692

H 6.17196 9.07401 15.18198

H 6.41146 2.43762 15.56935

C 4.34387 2.17796 15.0084

H 2.2852 2.22719 14.36072

H 2.01949 5.88532 13.63657

C 1.53604 4.20044 12.36119

C 0.66241 4.59311 14.69884

H 0.87972 11.5974 18.71344

C 1.16858 11.69206 20.8484

C 2.3153 9.811 21.86563

H 2.93242 8.23061 20.53248

H -0.57266 9.0524 19.41203

C -2.16328 8.16274 18.25341

C -1.55819 7.35733 16.04584

H 0.51163 7.60796 15.47392

H 6.68355 6.442 20.29046

C 5.0374 5.04736 20.36179

H 5.19529 8.93734 17.938

C 6.06651 9.25695 19.8973

C 7.16736 8.07183 17.95698

H 3.23277 3.8687 20.25671

H 0.17767 13.83603 14.90591

C 1.93987 14.27302 16.07224

H 3.72484 14.39844 17.27788

H 4.16741 10.77521 17.9887

C 4.6231 12.60799 19.02253

C 5.65489 11.82442 16.84237

H 0.82025 9.76587 9.65061

C 2.17999 11.37406 10.11974

H 2.14522 7.21552 12.00944

C 1.17152 7.17173 10.09001

C 0.14465 7.96386 12.26925

H 3.66278 12.7857 10.8018

H 5.06946 3.53621 9.81215

C 5.0565 5.43449 8.75899

H 5.03175 7.47136 8.00453

H 9.63106 8.23337 11.68111

C 9.19728 9.17836 13.58645

H 8.45958 9.99956 15.45735

H 4.34769 1.09693 15.18381

H 1.38864 3.10754 12.42349

H 2.33964 4.3931 11.63266

H 0.60089 4.63507 11.96669

H 0.45903 3.5209 14.8679

H -0.24994 5.0372 14.2635

H 0.83378 5.06284 15.67947

H 0.66535 12.66117 20.93688

C 1.68065 11.05633 21.99153

H 2.71082 9.30338 22.75219

H -2.91168 8.31919 19.03819

C -2.52874 7.55383 17.04344

H -1.8337 6.88289 15.09778

H 5.53527 4.37047 21.06418

H 6.67606 8.72405 20.64865

H 6.61802 10.1714 19.61687

H 5.12222 9.55784 20.37843

H 7.84139 7.49485 18.6145

H 7.02656 7.49629 17.02934

H 7.68432 9.01264 17.69868

H 1.82519 15.35898 15.98811

H 4.94464 13.6463 18.82697

H 3.71655 12.63938 19.64784

H 5.42482 12.12839 19.60791

H 5.91952 12.85299 16.53904

H 6.51107 11.40551 17.40086

H 5.51546 11.2275 15.92604

H 1.795 12.03407 9.33507

H 0.39862 7.70368 9.50745

H 0.80086 6.14649 10.25429

H 2.0846 7.10985 9.47657

H -0.59603 8.59666 11.7487

H 0.33091 8.40849 13.26077

H -0.30646 6.96625 12.41664

H 5.15307 4.97994 7.76706

H 10.2062 9.58713 13.70922

H 1.57938 11.52685 22.97552

H -3.56297 7.23368 16.87628

**^1^3^Me/Me^**

| Electronic Energy (UBP86) | −1409.335546 |
| --- | --- |
| Zero-point correction | 0.309421 |
| Thermal correction to Energy | 0.337334 |
| Thermal correction to Enthalpy | 0.338278 |
| Thermal correction to Gibbs Free Energy | 0.249284 |

Co 3.68981 7.2294 15.8872

Co 3.05216 9.25672 14.86815

N 4.59173 6.58131 14.48735

N 2.89875 7.52466 17.45957

N 2.14829 9.90395 16.26717

N 3.84388 8.96199 13.29598

Si 4.73602 7.47782 12.97895

Si 2.00197 9.00642 17.77479

C 2.77488 9.97316 19.21652

H 2.24994 10.92914 19.38925

H 2.72779 9.39905 20.1586

H 3.83355 10.19738 19.00435

C 0.18135 8.62706 18.1646

H -0.40091 9.55322 18.31467

H -0.27939 8.0635 17.33622

H 0.08267 8.0257 19.08545

C 1.51767 11.22345 16.13929

H 1.9129 11.9438 16.88486

H 1.69574 11.66279 15.13986

H 0.41823 11.17069 16.27869

C 3.00959 6.49386 18.49858

H 2.01508 6.1082 18.80355

H 3.59961 5.6258 18.1494

H 3.51008 6.8805 19.41007

C 5.22098 5.26107 14.61446

H 5.04317 4.82169 15.61392

H 4.82449 4.54128 13.86899

H 6.32038 5.31274 14.47432

C 3.7358 9.99426 12.2582

H 3.14701 10.86294 12.60792

H 4.7313 10.37851 11.95466

H 3.23546 9.60974 11.34571

C 6.55653 7.85201 12.58361

H 7.0213 8.41489 13.41021

H 7.1359 6.92424 12.43239

H 6.65403 8.45252 11.66208

C 3.9562 6.51265 11.53989

H 4.00187 7.08628 10.59745

H 4.47804 5.55519 11.36594

H 2.8976 6.29135 11.75551

**^3^3^Me/Me^**

| Electronic Energy (UBP86) | −1409.331444 |
| --- | --- |
| Zero-point correction | 0.310645 |
| Thermal correction to Energy | 0.338374 |
| Thermal correction to Enthalpy | 0.339318 |
| Thermal correction to Gibbs Free Energy | 0.249943 |

Co 3.68981 7.2294 15.8872

Co 3.05216 9.25672 14.86815

N 4.59173 6.58131 14.48735

N 2.89875 7.52466 17.45957

N 2.14829 9.90395 16.26717

N 3.84388 8.96199 13.29598

Si 4.73602 7.47782 12.97895

Si 2.00197 9.00642 17.77479

C 2.77488 9.97316 19.21652

H 2.24994 10.92914 19.38925

H 2.72779 9.39905 20.1586

H 3.83355 10.19738 19.00435

C 0.18135 8.62706 18.1646

H -0.40091 9.55322 18.31467

H -0.27939 8.0635 17.33622

H 0.08267 8.0257 19.08545

C 1.51767 11.22345 16.13929

H 1.9129 11.9438 16.88486

H 1.69574 11.66279 15.13986

H 0.41823 11.17069 16.27869

C 3.00959 6.49386 18.49858

H 2.01508 6.1082 18.80355

H 3.59961 5.6258 18.1494

H 3.51008 6.8805 19.41007

C 5.22098 5.26107 14.61446

H 5.04317 4.82169 15.61392

H 4.82449 4.54128 13.86899

H 6.32038 5.31274 14.47432

C 3.7358 9.99426 12.2582

H 3.14701 10.86294 12.60792

H 4.7313 10.37851 11.95466

H 3.23546 9.60974 11.34571

C 6.55653 7.85201 12.58361

H 7.0213 8.41489 13.41021

H 7.1359 6.92424 12.43239

H 6.65403 8.45252 11.66208

C 3.9562 6.51265 11.53989

H 4.00187 7.08628 10.59745

H 4.47804 5.55519 11.36594

H 2.8976 6.29135 11.75551

**^5^3^Me/Me^**

| Electronic Energy (UBP86) | −1409.354325 |
| --- | --- |
| Zero-point correction | 0.310518 |
| Thermal correction to Energy | 0.338376 |
| Thermal correction to Enthalpy | 0.339320 |
| Thermal correction to Gibbs Free Energy | 0.248775 |

Co 3.68981 7.2294 15.8872

Co 3.05216 9.25672 14.86815

N 4.59173 6.58131 14.48735

N 2.89875 7.52466 17.45957

N 2.14829 9.90395 16.26717

N 3.84388 8.96199 13.29598

Si 4.73602 7.47782 12.97895

Si 2.00197 9.00642 17.77479

C 2.77488 9.97316 19.21652

H 2.24994 10.92914 19.38925

H 2.72779 9.39905 20.1586

H 3.83355 10.19738 19.00435

C 0.18135 8.62706 18.1646

H -0.40091 9.55322 18.31467

H -0.27939 8.0635 17.33622

H 0.08267 8.0257 19.08545

C 1.51767 11.22345 16.13929

H 1.9129 11.9438 16.88486

H 1.69574 11.66279 15.13986

H 0.41823 11.17069 16.27869

C 3.00959 6.49386 18.49858

H 2.01508 6.1082 18.80355

H 3.59961 5.6258 18.1494

H 3.51008 6.8805 19.41007

C 5.22098 5.26107 14.61446

H 5.04317 4.82169 15.61392

H 4.82449 4.54128 13.86899

H 6.32038 5.31274 14.47432

C 3.7358 9.99426 12.2582

H 3.14701 10.86294 12.60792

H 4.7313 10.37851 11.95466

H 3.23546 9.60974 11.34571

C 6.55653 7.85201 12.58361

H 7.0213 8.41489 13.41021

H 7.1359 6.92424 12.43239

H 6.65403 8.45252 11.66208

C 3.9562 6.51265 11.53989

H 4.00187 7.08628 10.59745

H 4.47804 5.55519 11.36594

H 2.8976 6.29135 11.75551

**^7^3^Me/Me^**

| Electronic Energy (UBP86) | −1409.353220 |
| --- | --- |
| Zero-point correction | 0.310723 |
| Thermal correction to Energy | 0.338599 |
| Thermal correction to Enthalpy | 0.339543 |
| Thermal correction to Gibbs Free Energy | 0.248566 |

Co 3.68981 7.2294 15.8872

Co 3.05216 9.25672 14.86815

N 4.59173 6.58131 14.48735

N 2.89875 7.52466 17.45957

N 2.14829 9.90395 16.26717

N 3.84388 8.96199 13.29598

Si 4.73602 7.47782 12.97895

Si 2.00197 9.00642 17.77479

C 2.77488 9.97316 19.21652

H 2.24994 10.92914 19.38925

H 2.72779 9.39905 20.1586

H 3.83355 10.19738 19.00435

C 0.18135 8.62706 18.1646

H -0.40091 9.55322 18.31467

H -0.27939 8.0635 17.33622

H 0.08267 8.0257 19.08545

C 1.51767 11.22345 16.13929

H 1.9129 11.9438 16.88486

H 1.69574 11.66279 15.13986

H 0.41823 11.17069 16.27869

C 3.00959 6.49386 18.49858

H 2.01508 6.1082 18.80355

H 3.59961 5.6258 18.1494

H 3.51008 6.8805 19.41007

C 5.22098 5.26107 14.61446

H 5.04317 4.82169 15.61392

H 4.82449 4.54128 13.86899

H 6.32038 5.31274 14.47432

C 3.7358 9.99426 12.2582

H 3.14701 10.86294 12.60792

H 4.7313 10.37851 11.95466

H 3.23546 9.60974 11.34571

C 6.55653 7.85201 12.58361

H 7.0213 8.41489 13.41021

H 7.1359 6.92424 12.43239

H 6.65403 8.45252 11.66208

C 3.9562 6.51265 11.53989

H 4.00187 7.08628 10.59745

H 4.47804 5.55519 11.36594

H 2.8976 6.29135 11.75551

**^9^3^Me/Me^**

| Electronic Energy (UBP86) | −1409.285301 |
| --- | --- |
| Zero-point correction | 0.312453 |
| Thermal correction to Energy | 0.339925 |
| Thermal correction to Enthalpy | 0.340869 |
| Thermal correction to Gibbs Free Energy | 0.251909 |

Co 3.68981 7.2294 15.8872

Co 3.05216 9.25672 14.86815

N 4.59173 6.58131 14.48735

N 2.89875 7.52466 17.45957

N 2.14829 9.90395 16.26717

N 3.84388 8.96199 13.29598

Si 4.73602 7.47782 12.97895

Si 2.00197 9.00642 17.77479

C 2.77488 9.97316 19.21652

H 2.24994 10.92914 19.38925

H 2.72779 9.39905 20.1586

H 3.83355 10.19738 19.00435

C 0.18135 8.62706 18.1646

H -0.40091 9.55322 18.31467

H -0.27939 8.0635 17.33622

H 0.08267 8.0257 19.08545

C 1.51767 11.22345 16.13929

H 1.9129 11.9438 16.88486

H 1.69574 11.66279 15.13986

H 0.41823 11.17069 16.27869

C 3.00959 6.49386 18.49858

H 2.01508 6.1082 18.80355

H 3.59961 5.6258 18.1494

H 3.51008 6.8805 19.41007

C 5.22098 5.26107 14.61446

H 5.04317 4.82169 15.61392

H 4.82449 4.54128 13.86899

H 6.32038 5.31274 14.47432

C 3.7358 9.99426 12.2582

H 3.14701 10.86294 12.60792

H 4.7313 10.37851 11.95466

H 3.23546 9.60974 11.34571

C 6.55653 7.85201 12.58361

H 7.0213 8.41489 13.41021

H 7.1359 6.92424 12.43239

H 6.65403 8.45252 11.66208

C 3.9562 6.51265 11.53989

H 4.00187 7.08628 10.59745

H 4.47804 5.55519 11.36594

H 2.8976 6.29135 11.75551

**^5^3^Dipp/Ph^**

| Electronic Energy (UBP86) | −3886.813038 |
| --- | --- |
| Zero-point correction | 1.380226 |
| Thermal correction to Energy | 1.467707 |
| Thermal correction to Enthalpy | 1.468652 |
| Thermal correction to Gibbs Free Energy | 1.252368 |

Co 3.63844 7.29864 15.84656

Co 3.10067 9.18092 14.90853

N 4.30397 6.40988 14.38171

N 3.1216 7.66424 17.5716

H 1.37499 5.91502 17.74567

H 6.62642 6.14804 15.20794

N 2.40441 10.03501 16.37902

N 3.66266 8.80773 13.19967

H 5.08348 10.79274 13.62138

H 0.39352 10.10877 14.93332

Si 4.8 7.45151 13.04517

C 4.33066 4.99043 14.56634

Si 1.95448 8.97891 17.73509

C 3.74778 6.7996 18.52546

C 1.65468 5.25981 18.58859

C 6.84218 5.06598 15.23273

C 2.24672 11.45239 16.28107

C 3.1698 9.66321 12.16598

C 4.91023 11.50027 12.79124

C 0.06408 11.16173 14.88551

C 4.80703 6.61594 11.33832

C 6.57085 8.12436 13.26603

C 5.522 4.32866 15.02576

C 3.15239 4.20522 14.31212

C 1.94265 9.83187 19.4335

C 0.15903 8.3926 17.47167

C 5.06421 7.10093 19.02142

C 3.08521 5.61022 18.98862

C 0.68262 5.55397 19.75644

C 1.50673 3.79331 18.12787

C 7.81992 4.7544 14.07417

C 7.50159 4.73738 16.58993

C 1.14924 12.01373 15.53994

C 3.18006 12.33873 16.92603

C 2.10855 9.22529 11.29713

C 3.73147 10.97362 11.97583

C 6.19655 11.53564 11.93339

C 4.62892 12.89242 13.39695

C -1.26453 11.26966 15.6696

C -0.15498 11.53006 13.40213

C 4.8872 5.21093 11.17774

C 4.86857 7.41217 10.16573

C 7.57685 7.9025 12.29481

C 6.92495 8.88656 14.40569

C 5.49799 2.93687 15.22886

C 3.18802 2.81863 14.55032

C 1.86162 4.79863 13.74961

C 1.29581 11.08508 19.58805

C 2.44225 9.20716 20.60249

C -0.8361 8.57599 18.46195

C -0.23484 7.77073 16.26205

C 5.67847 6.20962 19.9208

C 5.82268 8.37455 18.65091

C 3.74685 4.76781 19.90045

H 0.75679 6.59982 20.0969

H -0.36122 5.37354 19.44738

H 0.9038 4.90195 20.62087

H 1.70802 3.08445 18.95054

H 0.47434 3.60698 17.78492

H 2.19285 3.5545 17.29871

H 8.09082 3.683 14.07051

H 8.74698 5.34351 14.17988

H 7.37988 4.99286 13.09197

H 6.82353 4.94414 17.43418

H 8.41359 5.34434 16.72417

H 7.80576 3.67736 16.65015

C 1.02253 13.41396 15.4608

C 3.00606 13.72719 16.79718

C 4.38023 11.82264 17.71626

C 1.6359 10.09683 10.30132

C 1.44762 7.857 11.44504

C 3.22276 11.79366 10.9503

H 6.44276 10.54099 11.52802

H 7.05478 11.87158 12.54041

H 6.08441 12.2344 11.08469

H 4.49946 13.65885 12.6122

H 5.47853 13.21033 14.02487

H 3.7224 12.89253 14.02334

H -1.64749 12.30611 15.65457

H -2.03136 10.61644 15.21885

H -1.14204 10.96475 16.7214

H 0.77558 11.45115 12.81738

H -0.90104 10.85418 12.95072

H -0.53919 12.55967 13.29129

H 4.86238 4.55894 12.05696

C 5.00866 4.62605 9.90516

C 4.98818 6.83114 8.89248

H 4.82668 8.50431 10.2436

H 7.34435 7.31737 11.39894

C 8.87397 8.42103 12.45055

C 8.21697 9.40958 14.56692

H 6.17196 9.07401 15.18198

H 6.41146 2.43762 15.56936

C 4.34387 2.17796 15.0084

H 2.2852 2.22719 14.36072

H 2.01949 5.88532 13.63657

C 1.53604 4.20044 12.36119

C 0.66241 4.59311 14.69884

H 0.87972 11.5974 18.71344

C 1.16858 11.69206 20.8484

C 2.3153 9.811 21.86563

H 2.93242 8.23061 20.53248

H -0.57266 9.0524 19.41203

C -2.16328 8.16274 18.25341

C -1.55819 7.35733 16.04584

H 0.51163 7.60796 15.47392

H 6.68355 6.442 20.29046

C 5.0374 5.04736 20.36179

H 5.19529 8.93734 17.938

C 6.06651 9.25695 19.8973

C 7.16736 8.07183 17.95698

H 3.23277 3.8687 20.25671

H 0.17767 13.83603 14.90591

C 1.93987 14.27302 16.07224

H 3.72484 14.39844 17.27788

H 4.16741 10.77521 17.9887

C 4.6231 12.60799 19.02253

C 5.65489 11.82442 16.84237

H 0.82025 9.76587 9.65061

C 2.18 11.37406 10.11974

H 2.14522 7.21552 12.00944

C 1.17152 7.17173 10.09001

C 0.14465 7.96386 12.26925

H 3.66278 12.7857 10.8018

H 5.06946 3.53621 9.81215

C 5.0565 5.43449 8.75899

H 5.03175 7.47136 8.00453

H 9.63106 8.23337 11.68111

C 9.19728 9.17836 13.58645

H 8.45958 9.99956 15.45735

H 4.34769 1.09693 15.18381

H 1.38864 3.10754 12.42349

H 2.33964 4.3931 11.63266

H 0.60089 4.63507 11.96669

H 0.45903 3.5209 14.86791

H -0.24994 5.0372 14.2635

H 0.83378 5.06284 15.67947

H 0.66535 12.66117 20.93688

C 1.68065 11.05633 21.99154

H 2.71082 9.30338 22.75219

H -2.91168 8.31919 19.03819

C -2.52874 7.55383 17.04345

H -1.8337 6.88289 15.09778

H 5.53527 4.37047 21.06418

H 6.67606 8.72405 20.64865

H 6.61802 10.1714 19.61687

H 5.12222 9.55784 20.37843

H 7.84139 7.49485 18.6145

H 7.02656 7.4963 17.02934

H 7.68432 9.01264 17.69868

H 1.82519 15.35898 15.98811

H 4.94464 13.6463 18.82697

H 3.71655 12.63938 19.64784

H 5.42482 12.12839 19.60791

H 5.91952 12.85299 16.53904

H 6.51107 11.40551 17.40086

H 5.51546 11.2275 15.92605

H 1.795 12.03407 9.33507

H 0.39862 7.70368 9.50745

H 0.80086 6.14649 10.25429

H 2.0846 7.10985 9.47657

H -0.59603 8.59666 11.7487

H 0.33091 8.40849 13.26077

H -0.30646 6.96625 12.41664

H 5.15307 4.97994 7.76706

H 10.2062 9.58713 13.70922

H 1.57938 11.52685 22.97552

H -3.56297 7.23368 16.87628

9. References

1. Chilton, N. F.; Anderson, R. P.; Turner, L. D.; Soncini, A.; Murray, K. S. PHI: A Powerful New Program for the Analysis of Anisotropic Monomeric and Exchange-Coupled Spin Systems. *J. Comput. Chem.* **2013**, *34*, 1164–1175.
2. Murugavel, R.; Palanisami, N.; Butcher, R. J. Synthesis, Characterization and Structures of Diphenyldiaminosilanes Bearing Bulky Substituents on Nitrogen. *J. Organomet. Chem.* **2003**, *675*, 65–71.
3. Ottmers, D. M.; Rase, H. F. Potassium Graphites Prepared by a Mixed-Reaction Technique. *Carbon* **1966**, *4*, 125–127.
4. Yang, D.; Guo, J. P.; Wu, H.; Ding, Y.; Zheng, W. Synthesis and Structural Characterization of Two-Coordinate Low-Valent 14-Group Metal Complexes Bearing Bulky Bis(amido)silane Ligands. *Dalton Trans.* **2012**, *41*, 2187–2194.
5. Evans, D. F. The Determination of the Paramagnetic Susceptibility of Substances in Solution by Nuclear Magnetic Resonance. *J. Chem. Soc.* **1959**, 2003–2005.
6. Sur, S. K. Measurement of Magnetic Susceptibility and Magnetic Moment of Paramagnetic Molecules in Solution by High-Field Fourier Transform NMR Spectroscopy. *J. Magn. Reson.* **1989**, *82*, 169–173.
7. Schubert, E. M. Utilizing the Evans Method with a Superconducting NMR Spectrometer in the Undergraduate Laboratory. *J. Chem. Educ.* **1992**, *69*, 62–64.
8. Hoye, T. R.; Eklov, B. M.; Ryba, T. D.; Voloshin, M.; Yao, L. J. No-D NMR (No-Deuterium Proton NMR) Spectroscopy: A Simple Yet Powerful Method for Analyzing Reaction and Reagent Solutions. *Org. Lett.* **2004**, *6*, 953–956.
9. Hoye, T. R.; Eklov, B. M.; Voloshin, M. No-D NMR Spectroscopy as a Convenient Method for Titering Organolithium (RLi), RMgX, and LDA Solutions. *Org. Lett.* **2004**, *6*, 2567–2570.
10. Bain, G. A.; Berry, J. F. Diamagnetic Corrections and Pascal’s Constants. *J. Chem. Educ.* **2008**, *85*, 532–536.
11. Sheldrick, G. M. SHELXT – Integrated Space-Group and Crystal-Structure Determination. *Acta Crystallogr. A* **2015**, *71*, 3–8.
12. Sheldrick, G. M. Crystal Structure Refinement with SHELXL. *Acta Crystallogr. C* **2015**, *71*, 3–8.
13. Dolomanov, O. V.; Bourhis, L. J.; Gildea, R. J.; Howard, J. A. K.; Puschmann, H. OLEX2: A Complete Structure Solution, Refinement and Analysis Program. *J. Appl. Crystallogr.* **2009**, *42*, 339–341.
14. Frisch, M. J.; Trucks, G. W.; Schlegel, H. B.; Scuseria, G. E.; Robb, M. A.; Cheeseman, J. R.; Scalmani, G.; Barone, V.; Petersson, G. A.; Nakatsuji, H.; Li, X.; Caricato, M.; Marenich, A. V.; Bloino, J.; Janesko, B. G.; Gomperts, R.; Mennucci, B.; Hratchian, H. P.; Ortiz, J. V.; Izmaylov, A. F.; Sonnenberg, J. L.; Williams-Young, D.; Ding, F.; Lipparini, F.; Egidi, F.; Goings, J.; Peng, B.; Petrone, A.; Henderson, T.; Ranasinghe, D.; Zakrzewski, V. G.; Gao, J.; Rega, N.; Zheng, G.; Liang, W.; Hada, M.; Ehara, M.; Toyota, K.; Fukuda, R.; Hasegawa, J.; Ishida, M.; Nakajima, T.; Honda, Y.; Kitao, O.; Nakai, H.; Vreven, T.; Throssell, K.; Montgomery, J. A., Jr.; Peralta, J. E.; Ogliaro, F.; Bearpark, M. J.; Heyd, J. J.; Brothers, E. N.; Kudin, K. N.; Staroverov, V. N.; Keith, T. A.; Kobayashi, R.; Normand, J.; Raghavachari, K.; Rendell, A. P.; Burant, J. C.; Iyengar, S. S.; Tomasi, J.; Cossi, M.; Millam, J. M.; Klene, M.; Adamo, C.; Cammi, R.; Ochterski, J. W.; Martin, R. L.; Morokuma, K.; Farkas, O.; Foresman, J. B.; Fox, D. J. Gaussian 16, Revision C.01; Gaussian, Inc.: Wallingford, CT, 2016.
15. Perdew, J. P. Density-Functional Approximation for the Correlation Energy of the Inhomogeneous Electron Gas. *Phys. Rev. B* **1986**, *33*, 8822–8824.
16. Becke, A. D. Density-Functional Exchange-Energy Approximation with Correct Asymptotic Behavior. *Phys. Rev. A* **1988**, *38*, 3098–3100.
17. Dolg, M.; Wedig, U.; Stoll, H.; Preuss, H. Energy-Adjusted *Ab Initio* Pseudopotentials for the First Row Transition Elements. *J. Chem. Phys.* **1987**, *86*, 866–872.
18. Martin, J. M. L.; Sundermann, A. Correlation Consistent Valence Basis Sets for Use with the Stuttgart–Dresden–Bonn Relativistic ECPs: The Atoms Ga–Kr and In–Xe. *J. Chem. Phys.* **2001**, *114*, 3408–3420.
19. Ditchfield, R.; Hehre, W. J.; Pople, J. A. Self-Consistent Molecular-Orbital Methods. IX. An Extended Gaussian-Type Basis for Molecular-Orbital Studies of Organic Molecules. *J. Chem. Phys.* **1971**, *54*, 724–728.
20. Hehre, W. J.; Ditchfield, R.; Pople, J. A. Self-Consistent Molecular-Orbital Methods. XII. Further Extensions of Gaussian-Type Basis Sets for Use in Molecular Orbital Studies of Organic Molecules. *J. Chem. Phys.* **1972**, *56*, 2257–2261.
21. Hariharan, P. C.; Pople, J. A. The Influence of Polarization Functions on Molecular Orbital Hydrogenation Energies. *Theor. Chim. Acta* **1973**, *28*, 213–222.
22. Francl, M. M.; Pietro, W. J.; Hehre, W. J.; *et al.* Self-Consistent Molecular Orbital Methods. XXIII. A Polarization-Type Basis Set for Second-Row Elements. *J. Chem. Phys.* **1982**, *77*, 3654–3665.
23. Gordon, M. S.; Binkley, J. S.; Pople, J. A.; *et al.* Self-Consistent Molecular Orbital Methods. XXV. Valence Basis Sets for Boron through Neon. *J. Am. Chem. Soc.* **1982**, *104*, 2797–2803.
24. Hay, P. J.; Wadt, W. R. Ab Initio Effective Core Potentials for Molecular Calculations. Potentials for the Transition-Metal Atoms Sc to Hg. *J. Chem. Phys.* **1985**, *82*, 299–310.
25. Hujon, F.; Lyngdoh, R. H. D.; Schaefer, H. F.; King, R. B. Binuclear Cobalt Paddlewheel-Type Complexes: Relating Metal–Metal Bond Lengths to Formal Bond Orders. *Inorg. Chem.* **2021**, *60*, 584–596.
26. Lu, T.; Chen, F. Multiwfn: A Multifunctional Wavefunction Analyzer. *J. Comput. Chem.* **2012**, *33*, 580–592.
27. Reed, A. E.; Curtiss, L. A.; Weinhold, F. Natural Population Analysis. *Chem. Rev.* **1988**, *88*, 899–926.
